# Titanium *cis*‐DACH *Salan* Catalyst for the Efficient Epoxidation of Nonactivated Olefins with Hydrogen Peroxide‐Terminal‐Selective Epoxidation of Multiply Unsaturated Terpenes

**DOI:** 10.1002/chem.202501688

**Published:** 2025-06-22

**Authors:** Christina Wartmann, Jörg.‐M. Neudörfl, Albrecht Berkessel

**Affiliations:** ^1^ Department of Chemistry, Organic Chemistry University of Cologne Greinstraße 4 D‐50939 Cologne Germany; ^2^ University of Cologne X‐Ray crystallography

**Keywords:** catalytic epoxidation, hydrogen peroxide, regioselectivity, salan ligands, titanium

## Abstract

We report a new generation of highly active and readily available homogeneous titanium catalysts for the epoxidation of nonactivated olefins with aqueous hydrogen peroxide. Key feature is the introduction of pentafluorophenyl substituents into a *salan* ligand derived from *cis*‐1,2‐diaminocyclohexane (*cis*‐DACH). Our novel *salan* ligand is accessible in one single step by reductive alkylation of *cis*‐DACH with 3‐(pentafluorophenyl)salicylic aldehyde. In situ complexation with Ti(O*i*Pr)_4_ of the *cis*‐DACH salan provides the titanium catalyst, which, in the presence of aqueous hydrogen peroxide, smoothly epoxidizes a broad spectrum of olefins with up to 95% yield at a catalyst loading of 0.5 mol‐% only. The achiral *cis*‐DACH salan catalyst showed *syn*‐selectivity (4.7:1) in the epoxidation of a chiral, racemic terminal allylic alcohol. This catalyst furthermore allows the regioselective (up to 49:1) epoxidation of the terminal double bond in multiply unsaturated terpenes such as myrcene, (*S*)‐citronellene, and (*R*)‐linalool. For the latter two substrates, *syn/anti*‐selectivity of up to 9:1 was observed. Augmented *syn/anti* selectivity (up to 50:1 *syn* or 25:1 *anti*) can be induced in the epoxidation of the chiral substrates (*S*)‐citronellene and (*R*)‐linalool when the “matched” enantiomer of the chiral “Berkessel *salalen* ligand” is employed.

## Graphical abstract

Terminal‐selective epoxidation–catalytic, simple, and with H_2_O_2_ as oxidant. The titanium complex of a new type of salan ligand, accessible in one step from *cis*‐DACH and 3‐(pentafluorophenyl)salicylic aldehyde, is an efficient catalyst for the epoxidation of nonconjugated olefins with H_2_O_2_. Most importantly, the novel catalyst is highly regioselective for terminal double bond epoxidation when multiply unsaturated substrates are employed.

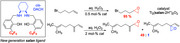

## 1 Introduction

**Figure 1:**
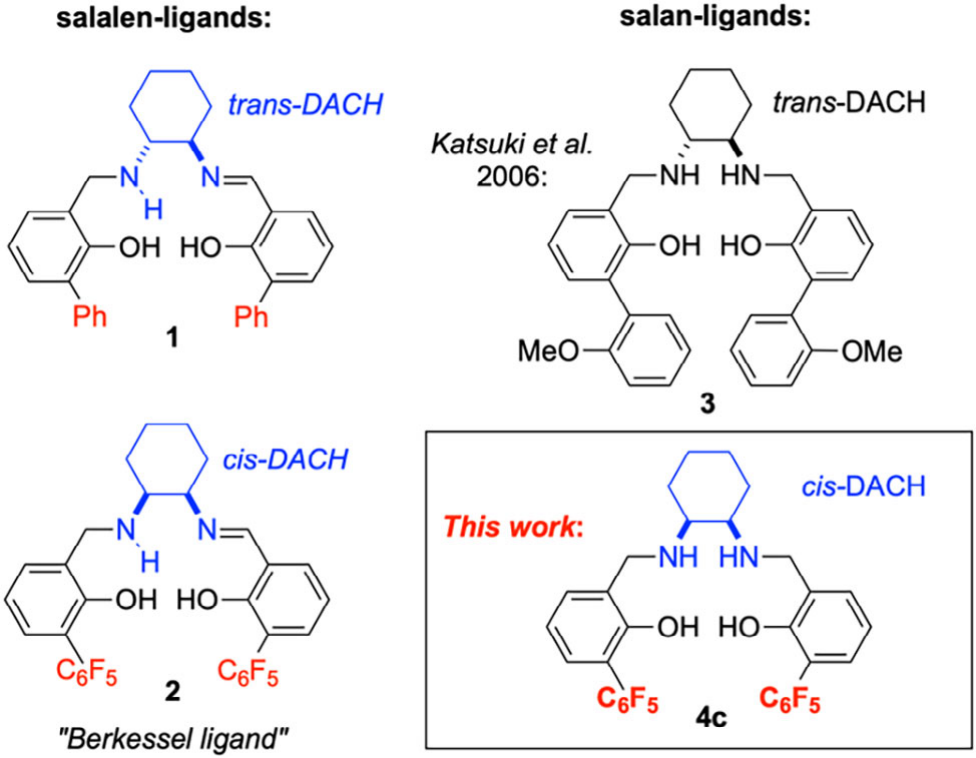
Salalen (left) and salan (right) ligands used in titanium‐catalyzed epoxidations of olefins with hydrogen peroxide.

Epoxides are highly valuable building blocks in organic synthesis, both in the academic laboratory and in the chemical industries.^[^ ^1^, ^2^ ^]^ They are typically prepared by oxygen transfer to olefins, with hydrogen peroxide being one of the most favorable terminal oxidants.^[^ ^3^ ^]^ The latter oxygen transfer typically requires hydrogen peroxide activation by a catalyst, and among the countless metal‐based and metal‐free systems investigated, titanium‐based catalysts clearly stand out. For example, the industrial epoxidation of propylene with hydrogen peroxide to propylene oxide (the HPPO process) is effected by substrate‐size‐limited titanium silicalite catalysts, such as TS‐1, at a scale exceeding 10^6^ t/a.^[^ ^4^ ^]^ With regard to *homogeneous* olefin epoxidation with hydrogen peroxide, the discovery by Katsuki et al. in 2005 of the titanium salalen motif^[^ ^5^ ^]^ has served as the basis of our own investigation and development of this catalyst class. An early example is represented by formula **1** (Figure 1).^[^ ^6^ ^]^ The Ti‐catalyst obtained from the in situ combination of the *trans*‐1,2‐diaminocyclohexane (*trans*‐DACH) derived ligand **1** (Figure 1) with Ti(O*i*Pr)_4_ readily epoxidizes electron‐rich olefins such as indene or 1,2‐dihydronaphthalene in high yield (90%) and ee (up to 98%).^[^ ^6^ ^]^ In contrast, low yields and enantioselectivities resulted for electron‐poor substrates, such as the “difficult” terminal olefin 1‐octene (6%, 60% ee). Several stages of development finally afforded the “Berkessel ligand” **2** (Figure 1): as crucial features, this ligand is derived from *cis*‐DACH and carries two pentafluorophenyl substituents *ortho* to the phenolic hydroxy groups.^[^ ^7^, ^8^, ^9^ ^]^ The titanium catalyst derived from ligand **2** (“Berkessel‐Katsuki catalyst”, BKC) allows the asymmetric epoxidation of terminal olefins (and many other types of olefins) in high yield and enantiopurity (typically >90% epoxide yield, > 95% ee) at catalyst loadings ≤1 mol‐%.^[^ ^9^, ^10^ ^]^

In 2006, one year after disclosing the first titanium *salalen* complex for epoxidation catalysis, Katsuki et al. reported that the related *salan* complexes show catalytic activity as well.^[^ ^11^ ^]^ As the most efficient ligand, the *trans*‐DACH‐derived salan **3** (Figure 1), carrying two (2‐methoxyphenyl)‐substituents *ortho* to the phenolic hydroxy groups, emerged from these studies.^[^ ^11^ ^]^ As in the case of our “early” salalen ligands (e.g., **1**, Figure 1), the titanium catalyst derived from Katsuki's salan **3** efficiently epoxidizes electron‐rich olefins, such as indene: 91% yield and 98% ee were reported at 2 mol‐% catalyst loading.^[^ ^11^ ^]^ Again in parallel to the “early” salalen ligand **1**, low yields and enantioselectivities resulted for electron‐poor substrates, such as terminal olefins (e.g., 25% yield and 50% ee for 1‐octene).

After the advent of the chiral BKC, the need for a simple, yet universally applicable homogeneous titanium catalyst for the non‐asymmetric epoxidation of a broad spectrum of olefins with hydrogen peroxide persisted.^[^ ^12^ ^]^ In contrast to their salalen counterparts, salan ligands can be accessed by simultaneous one‐step reductive alkylation of both N‐atoms of the diamine building block employed–thus making salan ligand synthesis extremely facile. Our extensive mechanistic studies on the “Berkessel‐Katsuki‐catalyst” (BKC), that is, the dimeric titanium complex derived from the *cis*‐DACH salalen ligand **2**, had revealed that the pentafluorophenyl substituents of ligand **2** enhance the reactivity of the oxygen transferring Ti‐OOH intermediate by virtue of their π‐acidity.^[^ ^13^, ^14^ ^]^ With this in mind, we set out to investigate the effect of pentafluorophenyl‐substitution on the epoxidation activity of titanium *cis*‐DACH *salan* complexes–hoping to arrive at a catalyst that, despite its simplicity, may be suitable for the epoxidation of low‐reactivity olefins.

## 2 Results and Discussion

**Scheme 1:**
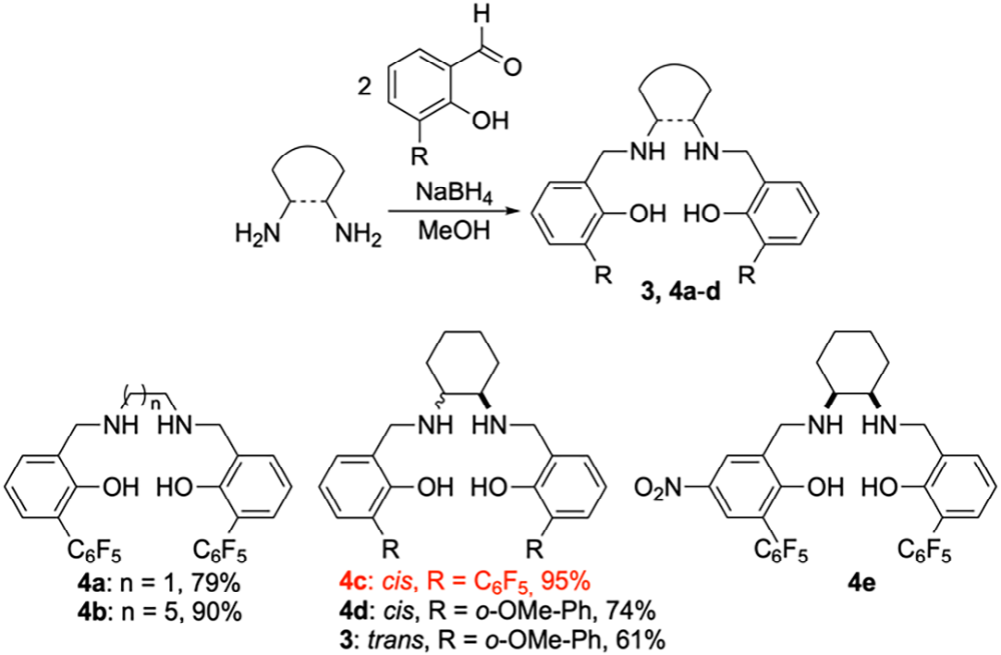
Top: one‐step synthesis of salan ligands such as **3**,**4a**‐**d** by reductive alkylation of diamines; bottom: structures of the ligands **3**, **4a**‐**e**.

The one‐step synthesis of the salan ligands **3** and **4a**‐**d** is summarized in Scheme 1. Note that for comparison, we also included simplified non‐DACH derived salans, namely the salans **4a** and **4b**, derived from ethylenediamine and hexamethylenediamine, respectively. Compound **4d** is the *cis*‐DACH analogue of Katsuki's salan **3**, while the ligand **4c** combines both new features, namely *cis*‐DACH backbone and pentafluorophenyl substituents. Additionally, the (chiral) ligand **4e** was accessed by NaBH_4_ reduction of a nitro‐derivative of the salalen **2**, described by us earlier.^[^ ^10^ ^]^

**Figure 2:**
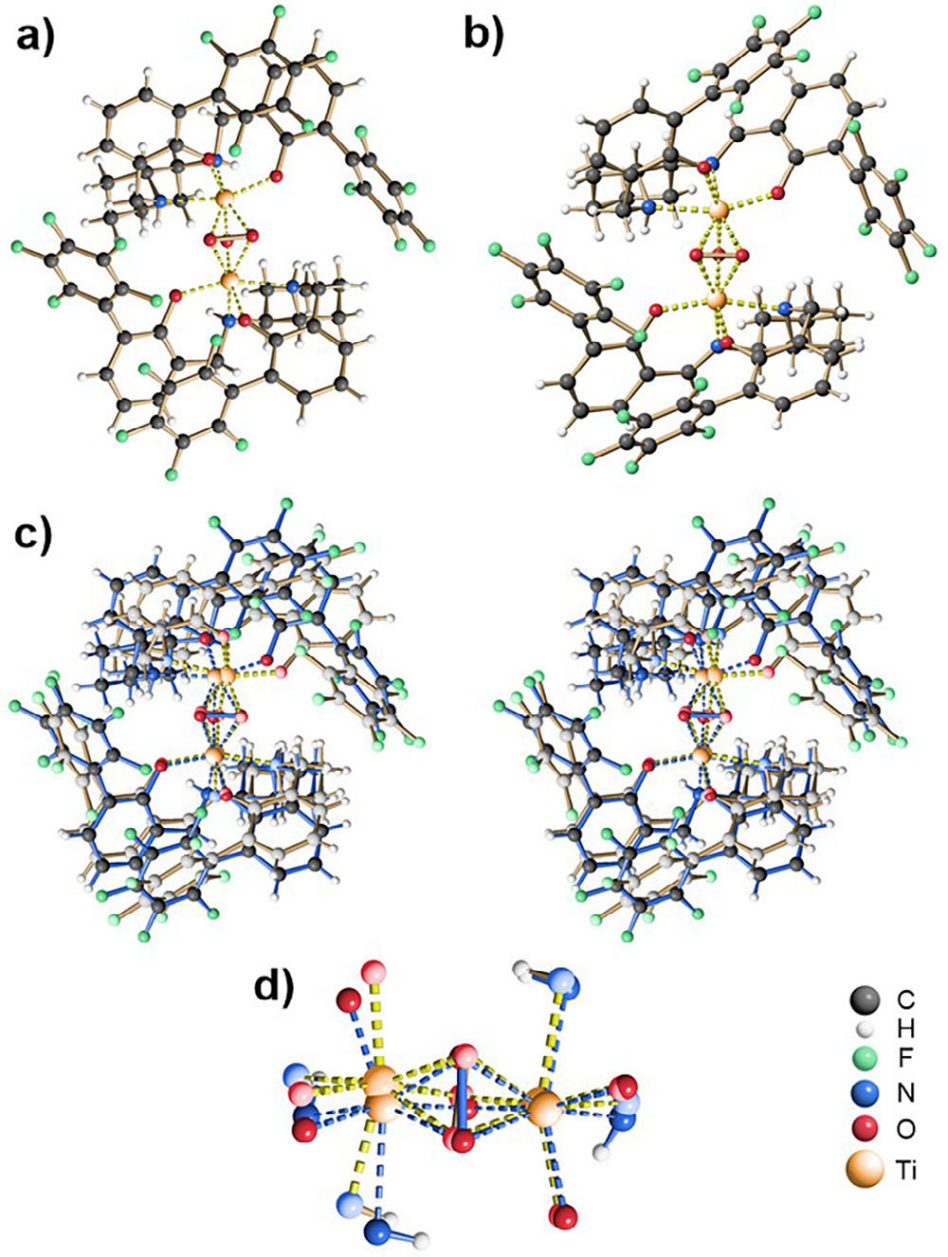
X‐ray crystal structures of dimeric μ‐oxo‐μ‐peroxo titanium complexes. a) Λ,Λ‐dimeric complex **5**, derived from the *salan* ligand **4c**; b) Λ,Λ‐dimeric complex ***6***, derived from the salalen ligand *rac*‐**2**; c) overlay of the X‐ray crystal structures of complexes **5** and **6**, in stereoscopic view; d) comparison of the titanium primary coordination spheres in complexes **5** and **6**; dark colors/blue bonds refer to complex **5**, while light colors/yellow bonds refer to complex **6**.

It was already shown by Katsuki et al. in 2006 that titanium complexes of salan ligands form dimeric di‐μ‐oxo bridged dimers.^[^ ^11^ ^]^ Also, the salan ligands shown in Scheme 1 readily form titanium complexes upon reaction with Ti(O*i*Pr)_4_. Upon exposure to hydrogen peroxide, we were able to crystallize the μ‐oxo‐μ‐peroxo dimer **5**, derived from the novel *cis*‐DACH salan ligand **4c**, its X‐ray crystal structure is shown in Figure 2a (see Supporting Information for crystallization conditions).^[^ ^15^ ^]^ For comparison, we also prepared and crystallized the μ‐oxo‐μ‐peroxo dimeric titanium complex derived from the *racemic* “Berkessel ligand” (*rac*‐**2**, Figure 1), its X‐ray crystal structure is shown in Figure 2b.^[^ ^15^ ^]^ Although derived from a salan (as in **5**) and a salalen (as in **6**) ligand, these two complexes show surprising structural similarity. First, the titanium centers in both complexes are coordinated in *cis*‐β mode. Second, both Ti‐complexes form racemic crystals composed of homochiral Λ,Λ‐, and Δ,Δ‐dimers. Figure 2a exemplarily shows the Λ,Λ‐dimer found in the crystals of **5**, while Figure 2b shows the Λ,Λ‐dimer of **6**. In the dimers, the homochiral subunits are oriented relative to one another in “trans” fashion.^[^ ^16^, ^17^ ^]^ To further demonstrate the structural similarity of **5** and **6**, Figure 2c provides a stereoscopic overlay of their crystal structures, and Figure 2d shows an overlay of the Ti centers of **5** and **6**, together with the O‐ and N‐atoms of their first coordination sphere.

**Figure 3:**
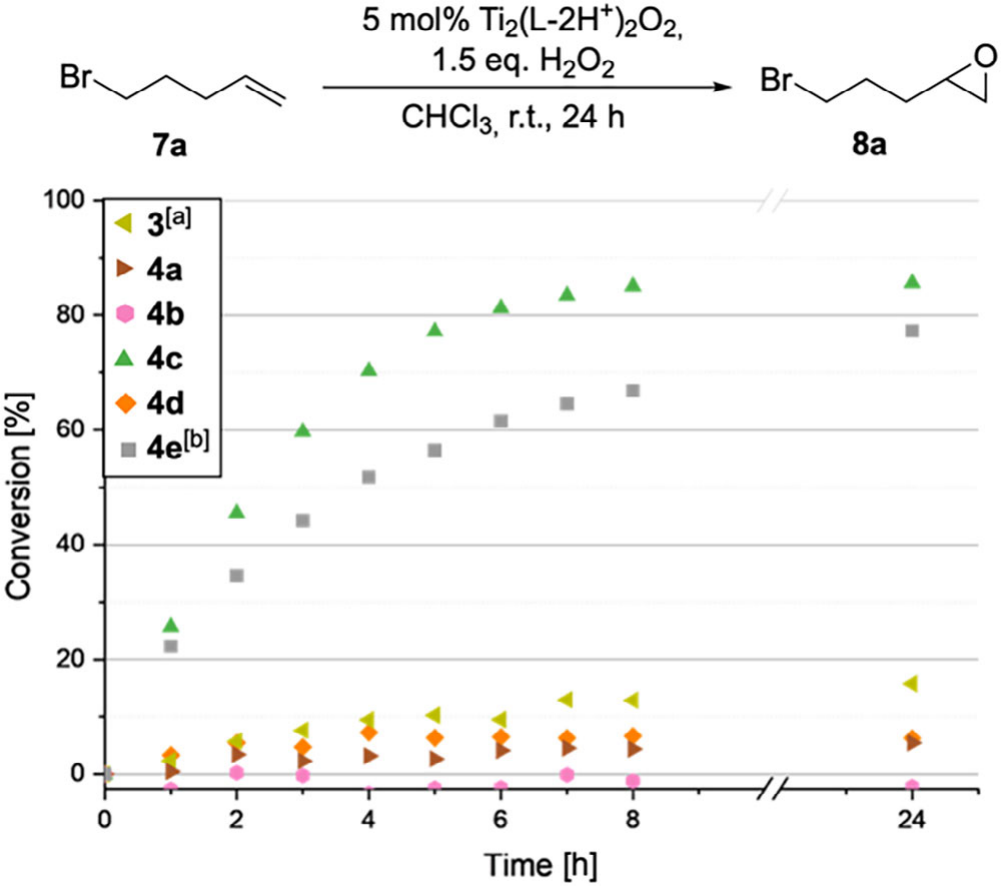
Comparison of the salan ligands **3**, **4a**‐**e** in the epoxidation of 5‐bromo‐1‐pentene (**7a**). a) epoxide (*R*)‐**8a**: 74% ee; b) epoxide (*R*)‐**8a**: 47% ee.

Figure 3 summarizes our assessment of the catalytic activity of the salan ligands **3**, **4a**‐**e**.^[^ ^18^ ^]^ The titanium complexes were generated in situ, simply by adding Ti(O*i*Pr)_4_ to a solution of the ligand. As a demanding test substrate, we chose the terminal olefin 5‐bromo‐1‐pentene (**7a**). We were delighted to see that both pentafluorophenyl‐substituted *cis*‐DACH salan ligands **4c** and **4e** indeed provided catalytic activity, with ligand **4c** giving the best result: at a catalyst loading of 5 mol‐%, 85% of the terminal olefin **7a** was converted to the racemic epoxide *rac*‐**8a** within 8 hours. We had observed before that the introduction of a nitro group into the salalen ligand **2** resulted in a substantial increase in reactivity.^[^ ^10^ ^]^ The analogous introduction of a nitro group into the salan ligand **4c**, however, had an adverse effect on its reactivity (ligand **4e**, 78% conversion, “second best”). Interestingly, the latter ligand induced some enantioselectivity [47% ee of the epoxide (*R*)‐**8a**]. All other ligands afforded less than 20% conversion of the terminal olefin **7a**, even after prolonged reaction times (up to 24 hours). Note that the failure of our “minimalistic” pentafluorophenyl‐substituted salan ligands **4a**,**b** underpins the importance of the active catalysts' *cis*‐DACH subunit.

**Scheme 2:**
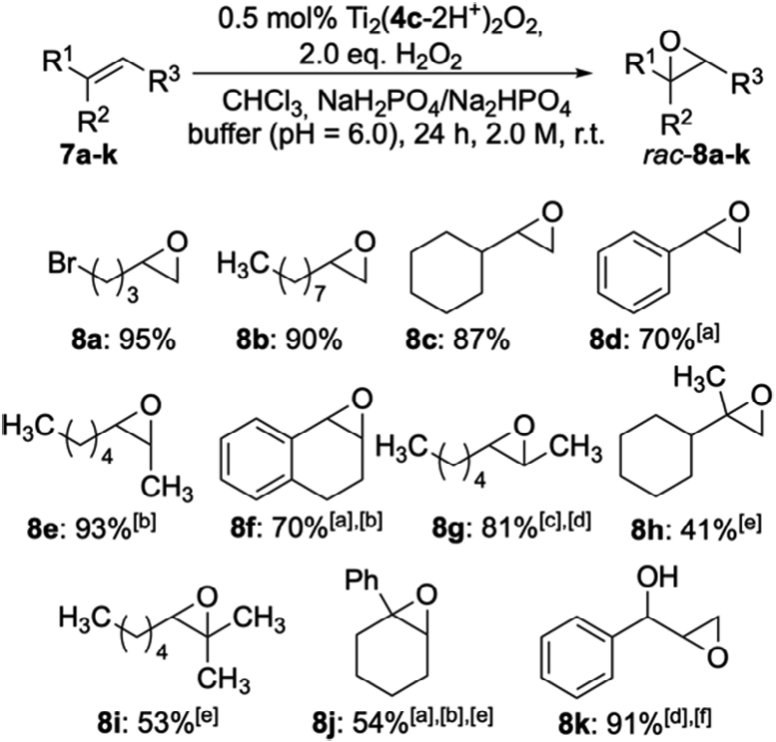
Epoxidation of various olefins using hydrogen peroxide and the Ti‐salan catalyst derived from ligand **4c**. a) 67 mM phosphate buffer of pH 6.7 was added; b) relative configuration: *cis*; c) relative configuration: *trans*; d) 1 mol% Ti_2_(**4c**‐2H^+^)_2_O_2_ was used; e) 2 mol% Ti_2_(**4c**‐2H^+^)_2_O_2_ were used; f) *syn/anti* = 4.7:1.

As the *cis*‐DACH salan ligand **4c** showed the highest reactivity in the preceding assessment, we continued on with this ligand to the optimization of reaction conditions (solvent, substrate concentration, catalyst loading, addition of buffer, etc.; see Supporting Information for details). Scheme 2 summarizes the isolated yields of (racemic) epoxides (*rac*‐**8a**‐**j**) obtained from a variety of olefinic structural motifs (**7a**‐**j**) under optimized conditions. At a catalyst loading as low as 0.5 mol‐%, nonconjugated terminal (**7a**‐**c**) and *cis*‐1,2‐disbstituted (**7e**) olefins gave the best results, with epoxide yields in the range of 87–95%. *cis*‐2‐Octene (**7e**) gave exclusively *cis*‐configurated epoxide *rac*‐**8e**, thus confirming the stereospecificity of the epoxidation. The epoxidation of *trans*‐2‐octene (**7** **g**) required slightly higher catalyst loading (1 mol‐%), only *trans*‐epoxide (*rac*‐**8 g**) was formed. For conjugated olefins such as styrene (**7d**) or 1,2‐dihydronaphthalene (**7f**), yields of racemic epoxide reached 70%. 2,2‐Disubstituted olefins (**7h**) and trisubstituted ones (**7i**,**j**) could be transformed as well, albeit at 41–54% yield. The epoxidation of the terminal allylic alcohol *rac*‐**7k** revealed a pronounced *syn*‐selectivity of the Ti‐catalyst derived from salan ligand **4c**: the racemic product epoxides *syn*‐ and *anti*‐*rac*‐**8k** were obtained in a ratio of 4.7:1 in a combined yield of 91%.^[^ ^19^ ^]^

Intrigued by the high reactivity–in particular toward terminal C ═ C double bonds–of the new titanium catalyst derived from salan **4c**, we decided to evaluate this system in the selective epoxidation of multiply unsaturated terpenes. We chose myrcene (**9**), (*S*)‐citronellene (**11**), and (*R*)‐linalool (**13**) as substrates (Table 1). Note that for these three monoterpenes, a terminal‐selective epoxidation has only been reported for (*R*)‐linalool (**13**): the Sharpless method using *tert*.‐butyl hydroperoxide (TBHP) as oxidant in the presence of catalytic VO(acac)_2_ gives almost exclusively the epoxide **14a**.^[^ ^20^, ^21^ ^]^ For obtaining the terminal epoxides of myrcene (**9**) and citronellene (**11**), alternative synthetic approaches had to be developed.^[^ ^22^ ^]^

**Table 1:** Titanium‐catalyzed epoxidation of myrcene (**9**), (*S*)‐citronellene (**11**), and (*R*)‐linalool (**13**) employing titanium complexes derived from the salan ligand **4c** or the salalen ligands **2** and *ent*‐**2**; yields stated are of isolated products.

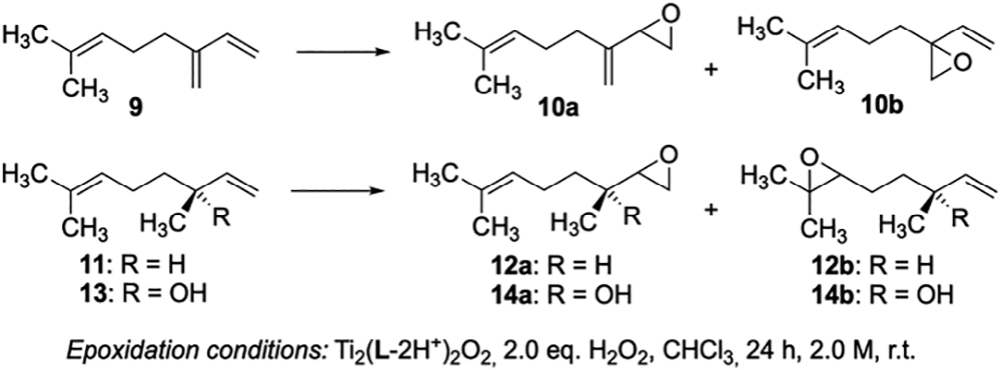						
Entry	Sub‐strate	Ligand L	Catalyst Loading [mol‐%]	Yield [%] 10,12,14	Regioselectivity^[a]^ 10,12,14a/10,12,14b	*syn*/*anti* ^[a]^ 12a, 14a
1	**9**	**4c** ^[d]^	2.0	48	49:1	‐
2	**9**	**2**	0.5	79	24:1^[b]^	‐
3	**11**	**4c** ^[d]^	1.5	51	6:1	3:2
4	**11**	**2**	0.5	54	3:1	1:25
5	**11**	*ent*‐**2**	0.5	50	6:1	50:1
6	**13**	**4c** ^[d]^	2	23^[c]^	3:1	9:1
7	**13**	**2**	1.5	42^[c]^	19:1	24:1
8	**13**	*ent*‐**2**	1.5	22^[c]^	3:1	1:4

^[a]^ Determined by ^1^H‐NMR;

^[b]^ The enantiomeric purity of **10a** was determined to be 30% ee by chiral GC;

^[c]^ The yields given are after column chromatography; however, the products **14a** and **14b** appear not to be stable on silica gel. Higher yields of approx. 80% could be obtained by bulb‐to‐bulb distillation. Under these conditions, however, a separation from the starting material (**13**) and some diepoxide could not be achieved;

^[d]^ Aqueous NaH_2_PO_4_/Na_2_HPO_4_ buffer (pH = 6.0, 67 mM) was added.

Our results obtained from the epoxidation of the terpenes **9**, **11,** and **13** are summarized in Table 1. For the epoxidation of the chiral substrates **11** and **13**, we also included the two enantiomers of the BKC [Ti_2_(**2**–2H^+^)_2_O_2_ and Ti_2_(*ent*‐**2**–2H^+^)_2_O_2_] in this study. Inspection of Table 1, entry 1 reveals that in the epoxidation of (achiral) myrcene (**9**), the new titanium salan catalyst derived from ligand **4c** indeed achieved unprecedented selectivity for the epoxidation of myrcene's (**9**) terminal C ═ C bond (49:1, vs. epoxidation of the 2,2‐disubstituted double bond). The salalen **2** provides a regioselectivity of 24:1, but at higher yield (entry 2). Note that other electrophilic oxidants (both catalytic and stoichiometric) exclusively epoxidize the triply substituted double bond of both myrcene (**9**) and citronellene (**10**)–the opposite of what is observed for our catalytic process(es).^[^ ^23^ ^]^ In the chiral monoterpenes (*S*)‐citronellene (**11**) and (*R*)‐linalool (**13**), the terminal double bond is sterically more hindered, due to the allylic mono/disubstitution. Nevertheless, in the presence of all three catalysts, epoxidation occurs predominantly at the terminal double bond (Table 1, entries 3–8), reaching a maximum regioselectivity of 19:1 in the epoxidation of (*R*)‐linalool (**13**, entry 7) using the salalen ligand **2**. For (*R*)‐linalool (**13**), this substrate‐catalyst combination represents the “matched pair” (compare entries 7 and 8), as it also achieved the highest *syn/anti*‐ratio of the product epoxide (24:1, terminal epoxide). For (*S*)‐citronellene (**11**), the enantiomeric catalyst, derived from the salalen ligand *ent*‐**2** appears to be the “matched” catalyst (entry 5), affording a 6:1 regioselectivity and a 50:1 *syn/anti*‐ratio. Still, the “mismatched” catalyst derived from ligand **2** effects a *syn/anti*‐ratio of 1:25, at a regioselectivity of 3:1 favoring terminal double bond epoxidation. For both (*S*)‐citronellene (**11**, entry 3) and (*R*)‐linalool (**13**, entry 6), our new Ti‐salan catalyst derived from ligand **4c** achieved regioselectivities of 3:1 to 6:1, with *syn/anti*‐ratios of up to 9:1. It needs to be emphasized at this point that fully terminal‐selective epoxidation of 1,4‐ and 1,6‐dienes with hydrogen peroxide had been achieved before by Strukul et al. using pentafluorophenyl Pt(II) diphosphine catalysts, even with high enantioselectivity (up to 98%).^[^ ^24a^ ^]^ However, this type of catalyst does not tolerate substituents in the terminal double bond's allylic position, and it is retarded by homoallylic substitution.^[^ ^24b^ ^]^ High terminal selectivity in the epoxidation of a 1,4‐ and a 1,6‐diene had also been observed by Mizuno et al., using hydrogen peroxide and polyoxovanadometallates as catalyst.^[^ ^24c,d^ ^]^

## 3 Conclusion

In summary, our mechanism‐based design of the novel *cis*‐DACH salan ligands incorporating pentafluorophenyl substituents has afforded the most readily available ligand **4c**. Upon in situ complexation with Ti(O*i*Pr)_4_, the ligand **4c** provides a titanium salan catalyst of high activity in the epoxidation of a variety of olefins with hydrogen peroxide: less than 1 mol‐% catalyst loading is typically needed for > 90% conversion of nonconjugated, in particular terminal olefins at ambient temperature and atmosphere. The novel titanium catalyst derived from salan ligand **4c**, together with the established BKC derived from ligand **2** (or *ent*‐**2**), proved to be highly regioselective in favor of terminal olefin epoxidation. Together with their structural similarity (in the solid state) and the remarkable effect of pentafluorophenyl substitution, this is another indication that the catalytic mechanism of the novel *cis*‐DACH salan catalyst may in fact parallel that of the BKC.^[^ ^13^ ^]^

## Supporting Information

The authors have cited additional references within the Supporting Information.^[^ ^25^, ^26^, ^27^, ^28^, ^29^, ^30^, ^31^, ^32^, ^33^, ^34^, ^35^, ^36^, ^37^, ^38^, ^39^, ^40^ ^]^

## Conflict of Interests

The authors declare no conflict of interest.

## Supporting information



Supporting information

## Data Availability

The data that support the findings of this study are available in the supplementary material of this article.
