# Supplementary material for: Titanium cis‐DACH Salan Catalyst for the Efficient Epoxidation of Nonactivated Olefins with Hydrogen Peroxide‐Terminal‐Selective Epoxidation of Multiply Unsaturated Terpenes
Source: Chemistry. 2025 Jun 22;31(39):e202501688. doi: 10.1002/chem.202501688 (PMC12258684; doi:10.1002/chem.202501688)
Supplement: Supplementary file 1 — Supporting information [file CHEM-31-e202501688-s001.pdf]

Supporting Information
©Wiley-VCH 2021
69451 Weinheim, Germany

Titanium *cis*-DACH *Salan* Catalyst for the Efficient Epoxidation of Non-Activated Olefins with Hydrogen Peroxide - Terminal-Selective Epoxidation of Multiply Unsaturated Terpenes

Christina Wartmann, Jörg-M. Neudörfl, and Albrecht Berkessel*

Abstract: We report a new generation of highly active and readily available homogeneous titanium catalysts for the epoxidation of non-activated olefins with aqueous hydrogen peroxide. Key feature is the introduction of pentafluorophenyl substituents into a *salan* ligand derived from *cis*-1,2-diaminocyclohexane (*cis*-DACH). Our novel *salan* ligand is accessible in one single step by reductive alkylation of *cis*-DACH with 3-(pentafluorophenyl)salicylic aldehyde. *In situ* complexation with Ti(O*i*Pr)$_4$ of the *cis*-DACH salan provides the titanium catalyst which, in the presence of aqueous hydrogen peroxide, smoothly epoxidizes a broad spectrum of olefins with up to 95 % yield, at a catalyst loading of 0.5 mol-% only. The achiral *cis*-DACH salan catalyst showed *syn*-selectivity (4.7:1) in the epoxidation of a chiral, racemic terminal allylic alcohol. This catalyst furthermore allows the regioselective (up to 49:1) epoxidation of the terminal double bond in multiply unsaturated terpenes such as myrcene, (*S*)-citronellene, and (*R*)-linalool. For the latter two substrates, *syn/anti*-selectivity of up to 9:1 was observed. Augmented *syn/anti*-selectivity (up to 50:1 *syn*, or 25:1 *anti*) can be induced in the epoxidation of the chiral substrates (*S*)-citronellene and (*R*)-linalool when the "matched" enantiomer of the chiral “Berkessel *salalen* ligand” is employed.

DOI: 10.1002/anie.2021XXXXX

Table of Contents

Table of Contents 2
1. Experimental Procedures 4
1.1 Materials/ Instrumentation 4
1.2 Ligand Synthesis 5
General Procedure A: Synthesis of Salan Ligands **3**, **4a-d** Starting from the Free Amines **S1-S3** 5
Synthesis of the Ethylendiamine-Salan **4a** 5
Synthesis of the 1,6-Diaminohexane-Salan **4b** 6
Synthesis of the *cis*-DACH-Salan **4c** 7
Synthesis of the *cis*-DACH-Salan **4d** 8
Synthesis of the *cis*-DACH-Salan **4e** 9
1.3 Optimization of the Epoxidation of 5-Bromo-1-pentene (**7a**) Using Ti-Salan Complexes 10
General Procedure B: Asymmetric Epoxidation of 5-Bromo-1-pentene (**7a**) Using Ti-Salan Complexes of a Substrate Concentration of 0.14 mol/L 10
Screening of the Salan Ligands **3,4a-e** in the Ti-catalyzed Epoxidation of 5-Bromo-1-pentene **7a** 10
Variation of the Amount of H_2O_2 in the Ti-catalyzed Epoxidation of 5-Bromo-1-pentene (**7a**) using the Salan Ligand **4c** 11
Solvent Screening of the Ti-catalyzed Epoxidation of 5-Bromo-1-pentene (**7a**) using the Salan Ligand **4c** 12
Additive Screening of the Ti-catalyzed Epoxidation of 5-Bromo-1-pentene (**7a**) using the Salan Ligand **4c** 13
Variation of the Substrate Concentration 14
Effect of Sodium Phosphate Buffers at a Substrate Concentration of 2.0 M 15
1.4 Crystallisation and X-ray Crystal Structure of the oxo-peroxo-Ti Complex of Salan **4c** 16
1.5 Crystallization and X-ray Crystal Structure of the Racemic Ti-Complex of Salalen **2** 18
1.6 General Procedure C: Epoxidation with the Ti-Complexes of Salalen **2** or Salan **4c** on Preparative Scale 19
1.7 Epoxidation with the Ti-Complex of Salan **4c** on Preparative Scale 20
Epoxidation of 5-Bromo-1-pentene (**7a**) 20
Epoxidation of 1-Decene (**7b**) 21
Epoxidation of Vinylcyclohexane (**7c**) 22
Epoxidation of Styrene (**7d**) 22
Epoxidation of *cis*-2-Octene (**7e**) 23
Epoxidation of 1,2-Dihydronaphthalene (**7f**) 24

Epoxidation of *trans*-2-Octene (**7g**)......24
Epoxidation of (1-Methylvinyl)-cyclohexane (**7h**)......25
Epoxidation of 2-Methyl-2-octene (**7i**)......26
Epoxidation of 1-Phenylcyclohexene (**7j**)......26
Epoxidation of α-Vinylbenzyl Alcohol (*rac*-**7k**)......27
1.8 Epoxidation of Terpenes with the Ti-Complexes of Salan **4c** or Salalen **2**......28
Epoxidation of Myrcene (**9**)......28
Epoxidation of (*S*)-Citronellene (**11**)......29
Epoxidation of (*R*)-Linalool (**13**)......30
2 NMR-Spectra......32
5-Bromo-1-pentene Oxide (*rac*-**8a**)......32
1-Decene Oxide (*rac*-**8b**)......33
2-Cyclohexyloxirane (*rac*-**8c**)......34
Styrene Oxide (*rac*-**8d**)......35
Cis-2-Octene Oxide (*rac*-**8e**)......36
1,2-Dihydronaphthalene Oxide (*rac*-**8f**)......37
Trans-2-Octene Oxide (*rac*-**8g**)......38
2-Methyl-2-cyclohexyloxirane (*rac*-**8h**)......39
2-Methyl-2-Octene Oxide (*rac*-**8i**)......40
1-Phenylcyclohexene Oxide (*rac*-**8j**)......41
1-(Oxiran-2-yl)-phenyl methanol (*rac*-**8k,** syn/anti-mixture)......42
Myrcene Oxides *rac*-**10a** and *rac*-**10b**......43
(S)-Citronellene Oxides **12a** and **12b**......44
(R)-Linalool Oxides **14a** and **14b**......47
3 GC......50
References......51

1. Experimental Procedures

1.1 Materials/ Instrumentation

Materials: Commercial reagents were purchased from Merck Sigma-Aldrich, Alfa Aesar, TCI, Carbolution, Arcos or BLD Pharm and used as received. Absolute solvents were purchased from Arcos. Salicylic aldehydes **S4,5** were prepared according to literature procedures.[1] The Berkessel ligand **2**, its enantiomer and the NO_2-salalen **S7** were prepared according to a procedure published previously by our workgroup;[2] **2** is also available at Merck Sigma-Aldrich (CAS: 2055467-90-6). The racemic mixture of the Berkessel-Katsuki catalyst **6** was obtained by mixing the two enantiomers, and crystallization following the already published procedure.[2] Salan **3** was prepared according to a literature procedure.[3]

Instruments: Nuclear magnetic resonance (^{1}H, ^{13}C, ^{19}F NMR) spectra were recorded on a Bruker Avance I 300, a Bruker Avance 400 or a Bruker Avance III 500 instrument. Chemical shifts are given in parts per million (ppm) downfield from tetramethylsilane (TMS) and are referenced to the solvent signal. Infrared (IR) spectra were recorded on a Shimadzu IRAffinity-1 FT-IR spectrometer with ATR technique. The data are reported in wave numbers (cm^{-1}) and intensity of absorption (s = strong, m = medium, w = weak, b = broad). GC-MS data were recorded on an Agilent Technologies 7890A instrument with an Agilent Technologies 5975C Triple-Axis detector (injector: 180 °C (split ratio: 50:1), oven: 50 °C (5 min), 20 °C/min, 280 °C (10 min). HRMS data were recorded either on a Thermo Scientific Exactive GC HR-GC-EI/MS with orbitrap analyzer (DIP-MS-HR-EI) or on a Thermo Scientific LTQ Orbitrap XL with an FTMS analyzer (ESI). For thin-layer chromatography Macherey-Nagel POLYGRAM® Sil G/UV 254 foils were used. They were coated with silica gel (layer thickness 250 µm) and fluorescence indicator. For detection UV light (λ = 254 nm) was used. Additionally, a cerium ammoniummolbydate stain (2.5 g ammoniummolbydate tetrahydrate, 1.0 g cerium(IV) sulfate, 10 ml sulfuric acid, 90 ml water) was used, to make non-UV active spots visible. Solvent mixtures are given in volume fraction at the appropriate positions. For column chromatography, Silica Gel 60 (0.035-0.07 mm) from Acros was used as stationary phase. Solvent mixtures are given in volume fraction at the appropriate positions. The flow rate was increased by over pressure through a hand pump.

1.2 Ligand Synthesis

*General Procedure A: Synthesis of Salan Ligands **3**, **4a-d** Starting from the Free Amines **S1-S3***

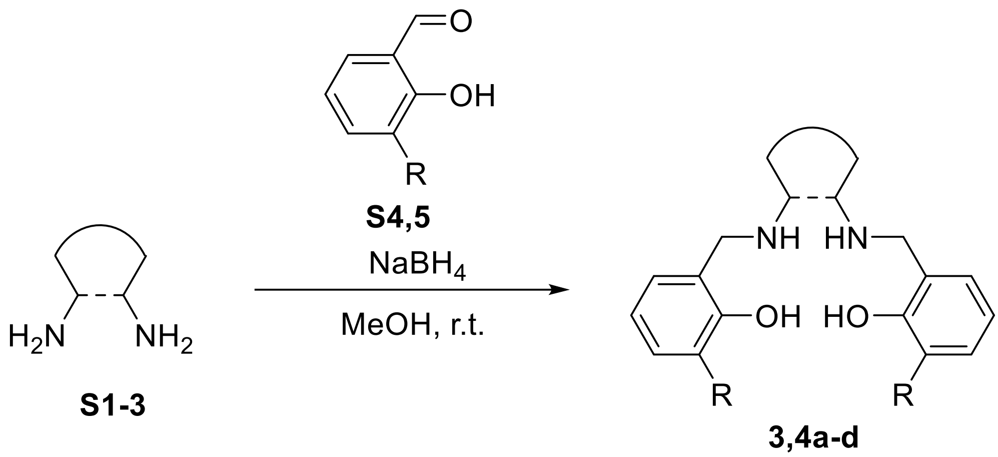

To a solution of salicylic aldehyde **S4,5** (6.94 mmol, 2.0 eq.) in 35 ml MeOH diamine **S1**-**S3** (3.47 mmol, 1.0 eq.) was added. The mixture was stirred at room temperature for 2 h, then $NaBH_4$ (0.34 g, 8.7 mmol, 2.5 eq.) was added. After 30 min. 50 ml water were added and the reaction was stirred for another 15 min. The aqueous phase was extracted with EtOAc or DCM, the combined organic phases were dried over Na_2SO_4 and the solvent was removed under reduced pressure. The residue was purified by column chromatography.

*Synthesis of the Ethylendiamine-Salan **4a***

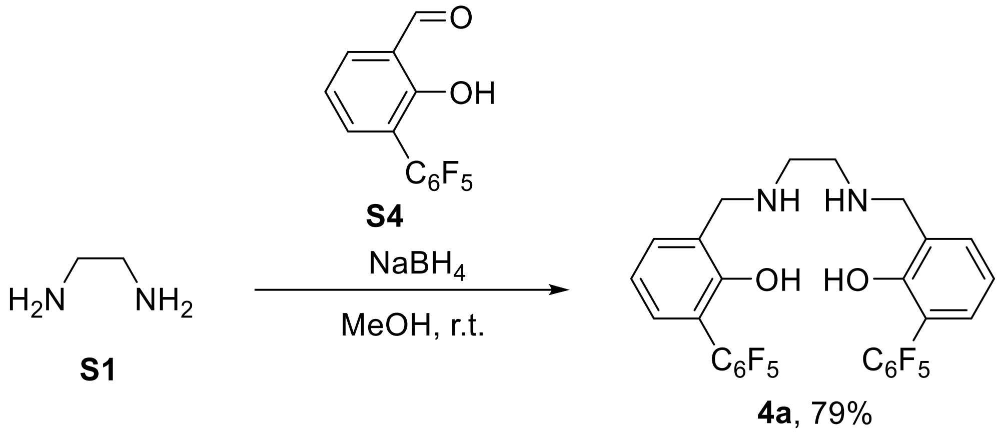

The ethylendiamine-salan **4a** was prepared according to *General Procedure A* on a 0.87 mmol scale. Before addition of $NaBH_4$ 50 ml DCM were added. The reaction mixture was extracted with DCM (2x50 mL). The crude product was purified by column chromatography (DCM:MeOH(NH_3) = 60:1) to give salan **4a** as a colourless solid in a yield of 414 mg (0.685 mmol, 79%).

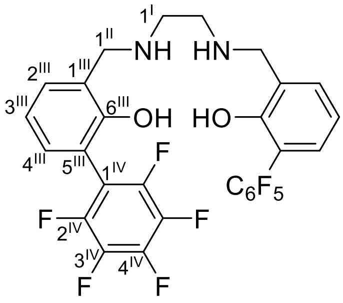

**4a**	M[$C_{32}H_{26}F_{10}N_2O_2$]: 604.4 g/mol.
**R$_f$**	0.37 (DCM:NH_3 sat. MeOH = 60:1).
**Mp.**	142 °C.
**^{1}H-NMR**	(400 MHz, $CDCl_3$) δ (ppm) = 7.14 (d, ^3J = 7.7 Hz, 2H, H-4^{III}), 7.09 (d, ^3J = 7.7 Hz, 2H, H-2^{III}), 6.88 (Ψ-t, ^3J = 7.7 Hz, 2H, H-3^{III}), 4.06 (s, 4H, H-1^{II}), 2.85 (s, 4H, H-1^{I}). The OH- and NH-signals were not detected.

$^{13}C\{^{19}F\}$-NMR (100 MHz, $CDCl_3$) δ (ppm) = 156.0 (2C, C-6^{III}), 144.5 (4C, C-2^{IV}), 140.5 (2C, C-4^{IV}), 137.6 (4C, C-3^{IV}), 130.9 (2C, C-4^{III}), 129.9 (2C, C-2^{III}), 122.6 (2C, C-1^{III}), 119.1 (2C, C-3^{III}), 114.0 (2C, C5^{III}), 112.7 (2C, C-1^{IV}), 52.5 (2C, C-1^{II}), 47.7 (2C, C-1^{I}).

^{19}F-NMR (376 MHz, $CDCl_3$) δ (ppm) = -140.02 – -140.15 (m, 4F, F-2^{IV}), -156.27 (t, ^3J = 20.8 Hz, 2F, F-4^{IV}), -162.87 – -163.25 (m, 4F, F-3^{IV}).

HR-MS (ESI): calculated for $[M+H]^+$: 605.12814, measured: 605.12783.

FT-IR (ATR): $\tilde{\nu}$ [cm^{-1}] = 2872 (w), 1518 (m), 1492 (s), 1427 (w), 1377 (w), 1219 (w), 1078 (m), 1049 (w), 987 (s), 980 (s), 858 (m), 752 (s).

Synthesis of the 1,6-Diaminohexane-Salan ***4b***

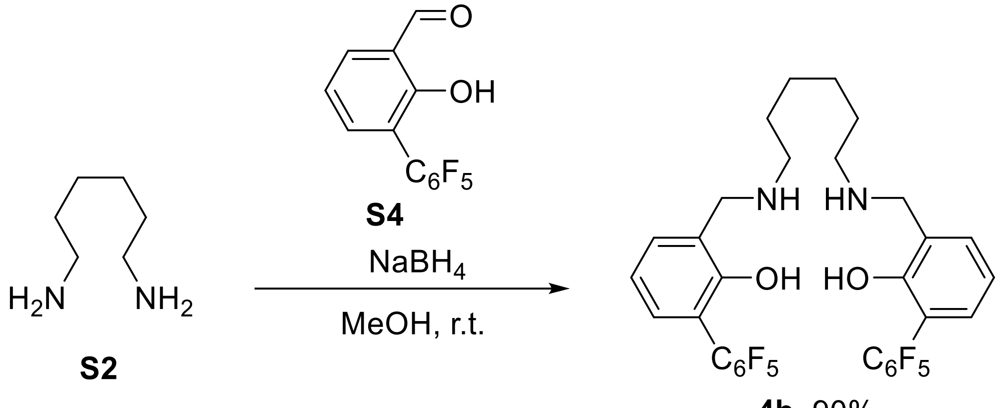

The 1,6-diaminohexane-salan **4b** was prepared according to *General Procedure A* on a 0.87 mmol scale. Before addition of $NaBH_4$ 40 mL DCM were added. The reaction mixture was extracted with DCM (2x50 mL). The crude product was purified by column chromatography (DCM:MeOH(NH_3) = 40:1) to give salan **4b** as a colourless solid in a yield of 414 mg (0.685 mmol, 79%).

4b M[$C_{32}H_{26}F_{10}N_2O_2$]: 660.54 g/mol.

R$_f$ 0.14 (DCM:NH_3 sat. MeOH = 40:1).

Mp. 104 °C.

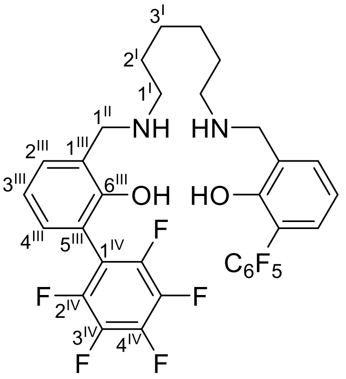

^1H-NMR (400 MHz, $CDCl_3$) δ (ppm) = 7.18 – 7.10 (m, 4H, H-2^{III}, H-4^{III}), 6.89 (Ψ-t, ^3J = 7.5 Hz, 2H, H-3^{III}), 4.08 (s, 4H, H-1^{II}), 2.70 (t, ^3J = 7.1 Hz, 4H, H-1^{I}), 1.59 – 1.49 (m, 4H, H-2^{I}), 1.41 – 1.32 (m, 4H, H-3^{I}).
The OH- and NH-signals were not deteced.

$^{13}C\{^{19}F\}$-NMR (100 MHz, $CDCl_3$) δ (ppm) = 156.5 (2C, C-6^{III}), 144.5 (4C, C-2^{IV}), 140.5 (2C, C-4^{IV}), 137.6 (4C, C-3^{IV}), 130.5 (2C, C-4^{III}), 129.7 (2C, C-2^{III}), 123.1 (2C, C-1^{III}), 118.7 (2C, C-3^{III}), 113.9 (2C, C-5^{III}), 112.9 (2C, C-1^{IV}), 52.7 (2C, C-1^{II}), 48.5 (2C, C-1^{I}), 29.3 (2C, C-2^{I}), 26.8 (2C, C-3^{I}).

^{19}F-NMR (376 MHz, $CDCl_3$) δ (ppm) = -139.91 – -140.10 (m, 4F, F-2^{IV}), -156.51 (t, ^3J = 21.0 Hz, 2F, F-4^{IV}), -163.01 – -163.44 (m, 4F, F-3^{IV}).

HR-MS (ESI): calculated for $[M+H]^+$: 661.19074, measured: 661.19074.

**FT-IR**	(ATR): $\tilde{\nu}$ [cm^{-1}] = 2978 (w), 2901 (w), 1519 (m), 1489 (s), 1402 (m), 1242 (m), 1080 (s), 1055 (m), 982 (s), 953 (m), 860 (s), 768 (s), 748 (s).

*Synthesis of the cis-DACH-Salan **4c***

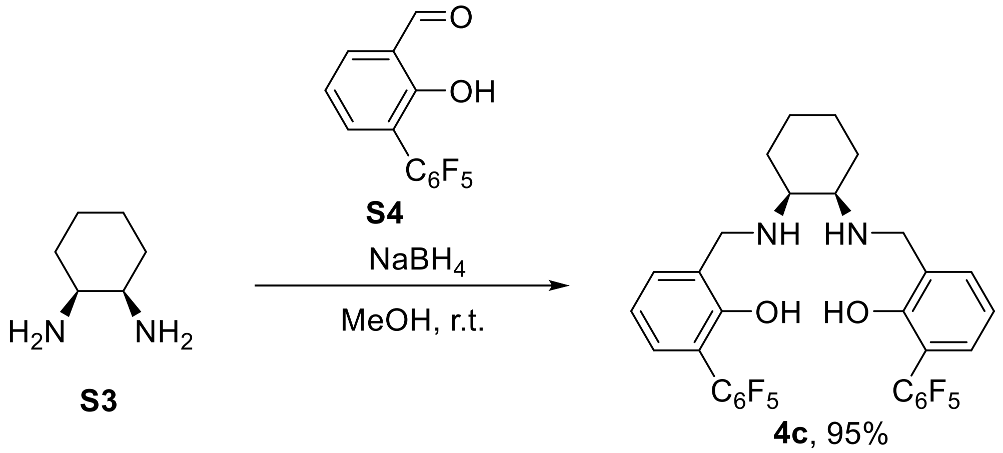

The *cis*-DACH-salan **4c** was prepared according to *General Procedure A* on a 3.47 mmol scale. The reaction mixture was extracted with EtOAc (1x200 mL, 2x50 mL). The crude product was purified by column chromatography (*c*-Hex:EtOAc = 3:1) to give salan **4c** as a colourless solid in a yield of 2.17 g (3.30 mmol, 95%).

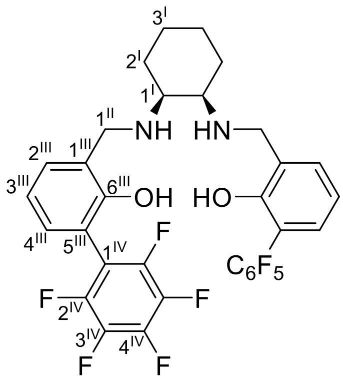

**4c**	M[$C_{32}H_{24}F_{10}N_2O_2$]: 658.5 g/mol.
**R_f**	0.28 (*c*-Hex:EtOAc = 3:1).
**Mp.**	180 C.
**^{1}H-NMR**	(400 MHz, $CDCl_3$) δ (ppm) = 7.14 (d, ^3J = 7.7 Hz, 2H, H-4^{III}), 7.08 (dd, ^3J = 7.7 Hz, ^4J = 1.7 Hz, 2H, H-2^{III}), 6.88 (Ψ-t, ^3J = 7.7 Hz, 2H, H-3^{III}), 4.08 (d, ^2J = 14.1 Hz, 2H, H-1^{II}a), 3.98 (d, ^2J = 14.1 Hz, 2H, H-1^{II}b), 2.92 – 2.85 (m, 2H, H-1^{I}), 1.74 – 1.34 (m, 8H, H-2^{I}, H-3^{I}). The OH- and NH-signals were not deteced.
**^{13}C{^{19}F}-NMR**	(100 MHz, $CDCl_3$) δ (ppm) = 156.1 (2C, C-6^{III}), 144.4 (4C, C-2^{IV}), 140.5 (2C, C-4^{IV}), 137.6 (4C, C-3^{IV}), 130.9 (2C, C-4^{III}), 129.9 (2C, C-2^{III}), 123.1 (2C, C-1^{III}), 119.0 (2C, C-3^{III}), 114.0 (2C, C-5^{III}), 112.7 (2C, C-1^{IV}), 55.6 (2C, C-1^{I}), 50.0 (2C, C-1^{II}), 27.2 (2C, C-2^{I}), 22.0 (2C, C-3^{I}).
**^{19}F-NMR**	(376 MHz, $CDCl_3$) δ (ppm) = -140.0 (ddd, ^3J = 55.5 Hz, ^4J = 23.5 Hz, ^4J = 8.1 Hz, 4F, F-2^{IV}), -156.30 (t, ^3J = 20.9 Hz, 2F, F-4^{IV}), -162.76 – -163.54 (m, 4F, F-3^{IV}).
**HR-MS**	(ESI): calculated for $[M+H]^+$: 659.17509, measured: 659.17480.
**FT-IR**	(ATR): $\tilde{\nu}$ [cm^{-1}] = 2978 (w), 2862 (w), 1518 (m), 1493 (s), 1456 (m), 1441 (m), 1425 (m), 1258 (w), 1207 (w), 1179 (w), 1157 (w), 1078 (m), 1049 (m), 982 (s), 964 (w), 854 (m), 750 (s).

*Synthesis of the cis-DACH-Salan **4d***

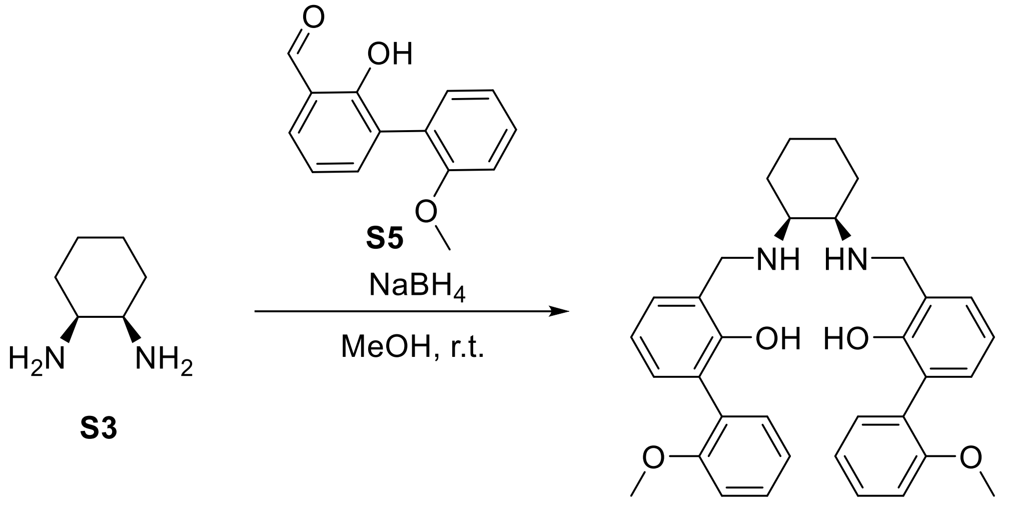

4d, 74%

The *cis*-DACH-salan **4d** was prepared according to *General Procedure A* on a 0.33 mmol scale. The reaction mixture was extracted with DCM (3x20 mL). The crude product was purified by column chromatography (*c*-Hex:EtOAc = 1:1) to give salan **4d** as a colourless solid in a yield of 135 mg (0.245 mmol, 74%).

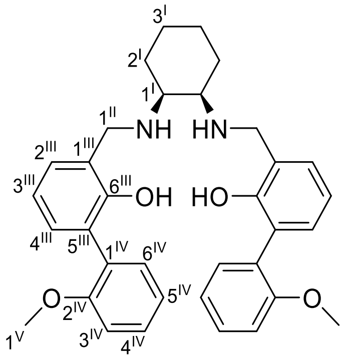

4d M[$C_{34}H_{38}N_2O_4$]: 538.68 g/mol.

R$_f$ 0.15 (*c*-Hex:EtOAc = 3:1).

Mp. 67 – 73 °C.

^{1}H-NMR (400 MHz, CDCl$_3$) δ (ppm) = 7.38 – 7.29 (m, 4H, H-4^{IV}, H-6^{IV}), 7.20 (dd, ^3J = 7.6 Hz, ^4J = 1.7 Hz, 2H, H-4^{III}), 7.07 – 6.95 (m, 6H, H-2^{III}, H-3^{IV}, H-5^{IV}), 6.85 (Ψ-t, ^3J = 7.6 Hz, 2H, H-3^{III}), 4.02 (d, ^2J = 13.6 Hz, 2H, H-1^{II}a), 3.92 (d, ^2J = 13.6 Hz, 2H, H-1^{II}b), 3.79 (s, 6H, H-1^{V}), 2.95 – 2.85 (m, 2H, H-1^{I}), 1.71 – 1.62 (m, 2H, H-2^{I}a), 1.60 – 1.47 (m, 4H, H-2^{I}b, H-3^{I}a), 1.43 – 1.34 (m, 2H, H-3^{I}b).
The OH- and NH-signals were not deteced.

^{13}C-NMR (100 MHz, CDCl$_3$) δ (ppm) = 156.9 (2C, C-2^{IV}), 155.2 (2C, C-6^{III}), 131.8 (2C, C-4^{IV}), 130.8 (2C, C-4^{III}), 128.7 (2C, C-6^{IV}), 128.1 (2C, C-2^{III}), 127.6 (2C, C-1^{IV}), 126.3 (2C, C-5^{IV}), 123.4 (2C, C-1^{III}), 120.6 (2C, C-5^{III}), 118.72C, C-3^{III}), 111.3 (2C, C-3^{IV}), 55.8 (2C, C-1^{V}), 55.5 (2C, C-1^{I}) 50.0 (2C, C-1^{II}), 27.4 (2C, C-2^{I}), 22.2 (2C, C-3^{I}).

HR-MS (ESI): calculated for [M+H]$^+$: 539.29043, measured: 539.28985.

FT-IR (ATR): $\tilde{\nu}$ [cm^{-1}] = 2924 (w), 2854 (w), 1589 (w), 1496 (w), 1445 (m), 1429 (m), 1265 (m), 1234 (m), 1179 (w), 1122 (w), 1080 (w), 1067 (w), 1024 (w), 831 (w), 748 (s).

Synthesis of the cis-DACH-Salan ***4e***

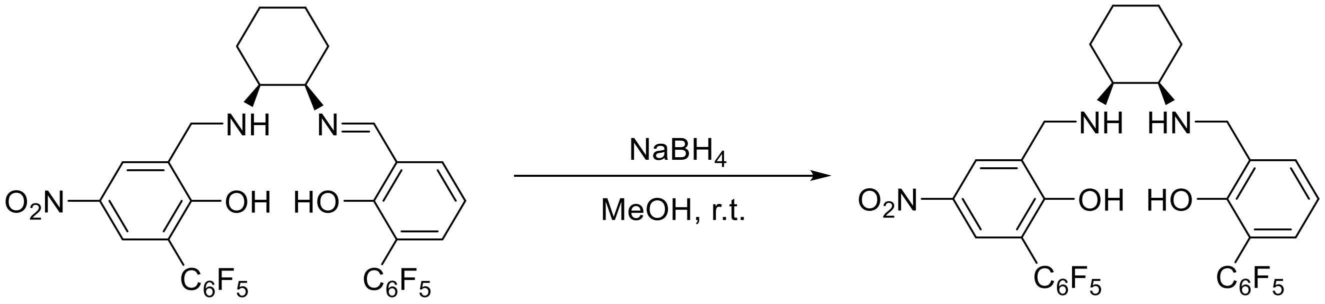

S7 → **4e**, 90%

301 mg (0.428 mmol, 1.0 eq.) of the salalen-ligand **S7** were dissolved in 10 mL MeOH. 25 mg (0.64 mmol, 1.5 eq.) $NaBH_4$ were added, and the mixture was stirred at room temperature overnight. 30 mL water were added, and the aqueous phase was extracted (4x15 mL DCM). The combined organic phases were dried over Na_2SO_4, and the solvent was removed. The residue was purified by column chromatography (c-Hex:EtOAc = 1:1) to give **4e** in a yield of 270 mg (0.384 mmol, 90%) as a pale-yellow solid.

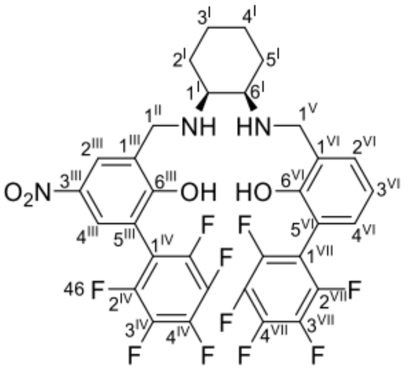

4e M[$C_{32}H_{23}F_{10}N_3O_4$]: 703.5 g/mol

R_f 0.31 (*c*-Hex:EtOAc = 1:1).

Mp. 91°C.

^{1}H-NMR (400 MHz, DMSO-d_6) δ (ppm) = 8.14 (d, ^4J = 3.1 Hz, 1H, H-2^{III}), 8.03 (d, ^4J = 3.1 Hz, 1H, H-4^{III}), 7.35 (dd, ^3J = 7.6 Hz, ^4J = 1.7 Hz, 1H, H-2^{VI}), 7.14 (dd, ^3J = 7.6 Hz, ^4J = 1.7 Hz, 1H, H-4^{VI}), 6.88 (t, ^3J = 7.6 Hz, 1H, H-3^{VI}), 4.11 (s, 2H, H-1^{II}), 3.92 (d, ^2J = 12.1 Hz, 1H, H-1^{Va}), 3.70 (d, ^2J = 12.1 Hz, 1H, H-1b^{V}), 3.22 – 3.10 (m, 1H, H-1^{I}), 3.05 – 2.97 (m, 1H, H-6^{I}), 1.85 – 1.69 (m, 2H, H-2^{Ia}, H-5^{Ia}), 1.66 – 1.45 (m, 4H, H-2^{Ib}, H-3^{I}/H-4^{I}, H-5^{Ib}), 1.41 – 1.28 (m, 2H, H-4^{I}/H-3^{I}).
The OH- and NH-signals were not detected.

^{13}C{^{19}F}-NMR (100 MHz, DMSO-d_6) δ (ppm) = 174.3 (1C, C-6^{III}), 154.7 (1C, C-6^{VI}), 144.7 (2C, C-2^{IV}), 144.3 (2C, C-2^{VII}), 140.1 (1C, C-4^{IV}), 139.5 (1C, C-4^{VII}), 137.4 – 137.2 (4C, C-3^{IV}, C-3^{VII}), 133.5 (1C, C-2^{VI}), 131.2 (1C, C-4^{VI}), 130.8 (1C, C-3^{III}), 129.3 (1C, C-4^{III}), 128.2 (1C, C-2^{III}), 125.9 (1C, C-1^{VI}), 121.7 (1C, C-1^{III}), 119.4 (1C, C-3^{VI}), 114.9 (1C, C-5^{III}), 114.2 (1C, C-5^{VI}), 114.1 (1C, C-1^{IV}), 113.9 (1C, C-1^{VII}), 55.8 (1C, C-1^{I}), 54.8 (1C, C-6^{I}), 47.8 (1C, C-1^{II}/C-1^{V}), 47.7 (1C, C-1^{V}/C-1^{II}), 26.1 (1C, C-5^{I}), 24.4 (1C, C-2^{I}), 21.7 (1C, C-3^{I}/C-4^{I}), 20.92 (1C, C-4^{I}/C-3^{I})

^{19}F-NMR (376 MHz, $CDCl_3$) δ (ppm) = -139.7 – -140.4 (m, 4F, F-2^{IV}), -156.41 (t, ^3J = 20.9 Hz, 2F, F-4^{IV}), -163.0 – -163.3 (m, 4F, F-3^{IV}).

HR-MS (ESI): calculated for $[M+H]^+$: 659.17509, measured: 659.17517.

FT-IR (ATR): $\tilde{\nu}$ [cm^{-1}] = 2947 (w), 1520 (m), 1491 (s), 1440 (m), 1316 (w), 1223 (w), 1188 (m), 1078 (m), 982 (s), 899 (w), 854 (m), 768 (s), 744 (s), 648 (m).

1.3 Optimization of the Epoxidation of 5-Bromo-1-pentene (7a) Using Ti-Salan Complexes

*General Procedure B: Asymmetric Epoxidation of 5-Bromo-1-pentene (**7a**) Using Ti-Salan Complexes, at a Substrate Concentration of 0.14 mol/L*

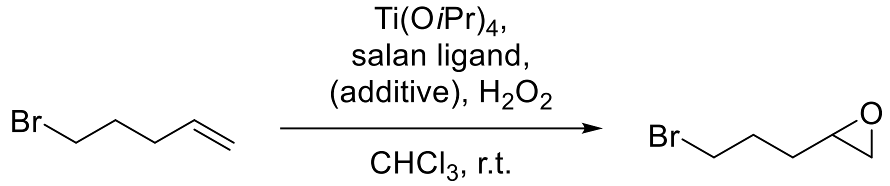

7a **8a**

In a glovebox, a solution of Ti(OiPr)$_4$ in 1 mL absolute DCM was added to the salan ligand. The solution was taken from the glovebox and stirred for 1 h. The solvent was removed under reduced pressure. Afterwards, a solution of 5-bromo-1-pentene (**7a**, 14.9 mg, 100 µmol, 1.0 eq.), bromobenzene (15.7 mg, 100 µmol, 1.0 eq., internal standard) and additive (where applicable) in 0.70 mL $CHCl_3$ was added. The mixture was stirred for 15 min, then H_2O_2 (50 w%) was added. The reaction mixture was stirred at room temperature. The reaction progress was monitored by GC: A 10 µL aliquot was withdrawn and diluted with EtOAc (approx. 0.4 ml). The solution was filtered through a mixture of $MgSO_4$ and MnO_2 (10:1) into a GC-vial with inlay (100 µL volume) and submitted to GC-analysis. A sample was taken before H_2O_2 was added and submitted to GC-analysis as sample for t = 0. Conversion of 5-bromo-1-pentene (**7a**) was determined from the ratio of the integrals between 5-bromo-1-pentene (**7a**) and bromobenzene over time.

*Screening of the Salan Ligands **3,4a-e** in the Ti-catalyzed Epoxidation of 5-Bromo-1-pentene **7a***

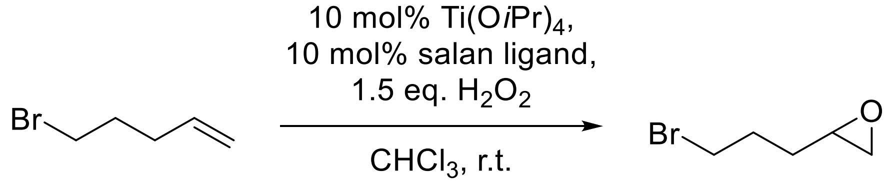

7a **8a**

The salan ligands **3,4a-e** were tested in the epoxidation of 5-bromo-1-pentene (**7a**) according to *General Procedure B*. 10 µmol (10 mol%) Ti(O*i*Pr)$_4$, 10 µmol (10 mol%) of the corresponding salan ligand and 8.5 µL (50 w%, 150 µmol 1.5 eq.) of H_2O_2 were used. $CHCl_3$ was used as solvent The time-conversion-profiles are shown in **Figure S1**.

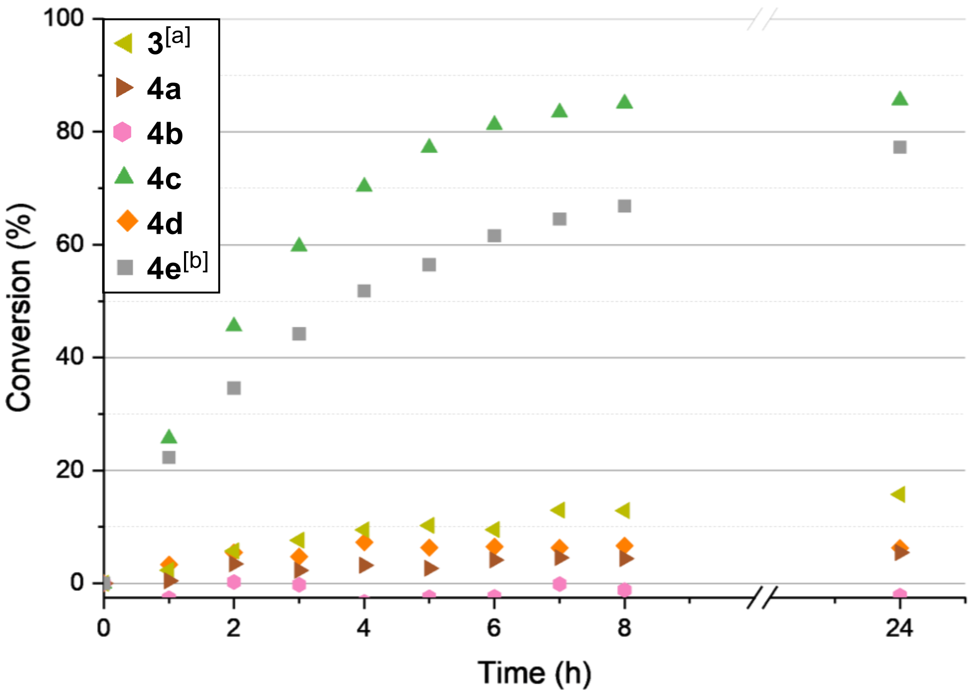

Figure S1: Comparison of the salan ligands **3,4a-e** in the epoxidation of 5-bromo-1-pentene (**7a**). The following enantioselectivities were observed: ligand **3**: 74%*ee*; ligand **4e**: 47%*ee*.

*Variation of the Amount of H_2O_2 in the Ti-catalyzed Epoxidation of 5-Bromo-1-pentene (**7a**) using the Salan Ligand **4c***

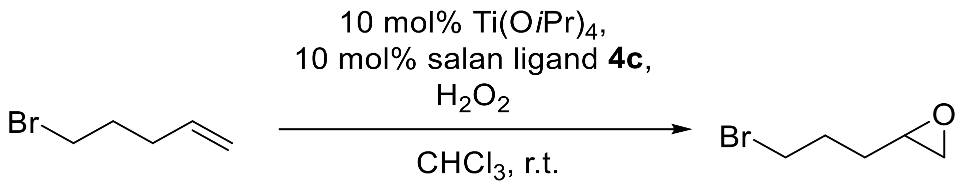

7a **8a**

The reactions were performed according to *General Procedure B*. 10 µmol (10 mol%) Ti(O*i*Pr)$_4$ and 10 µmol (10 mol%) salan ligand **4c** were used. $CHCl_3$ was used as solvent. The amount of H_2O_2 was varied between 8.5 µL (50 w%, 150 µmol, 1.5 eq.) and 17 µL (50 w%, 300 µmol, 3.0 eq.). The conversion-time-profiles are shown in **Figure S2**.

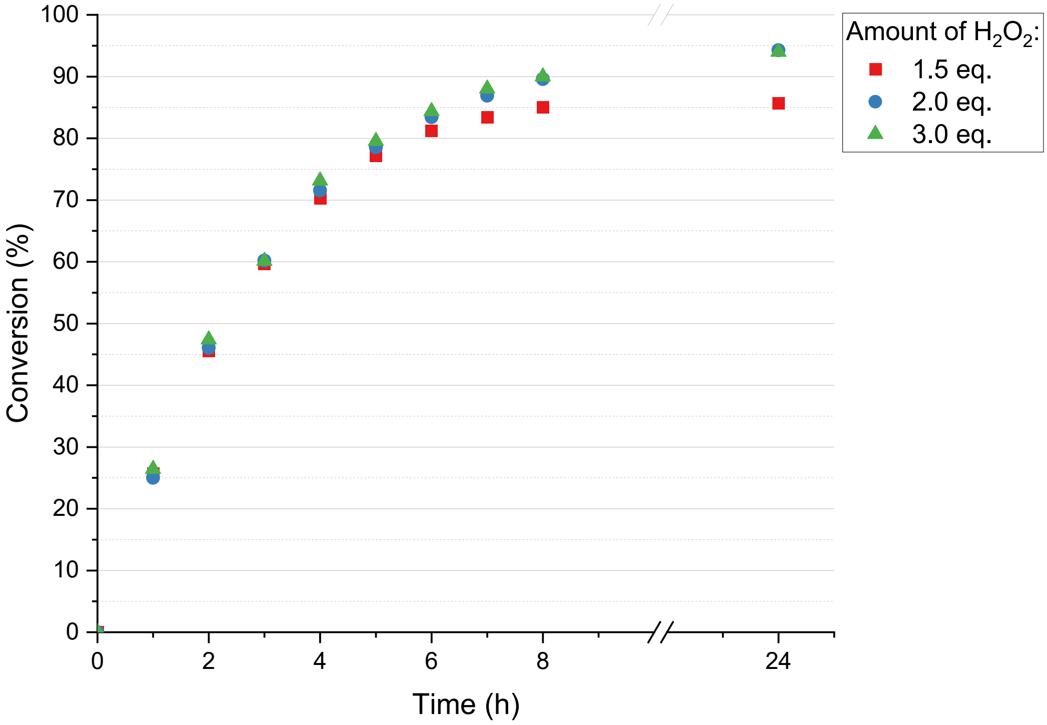

Figure S2: Epoxidation of 5-bromo-1-pentene (**7a**) with the Ti-complex of the salan ligand **4c** as catalyst with varying amounts of H_2O_2 added to the reactions.

*Solvent Screening of the Ti-catalyzed Epoxidation of 5-Bromo-1-pentene (**7a**) using the Salan Ligand **4c***

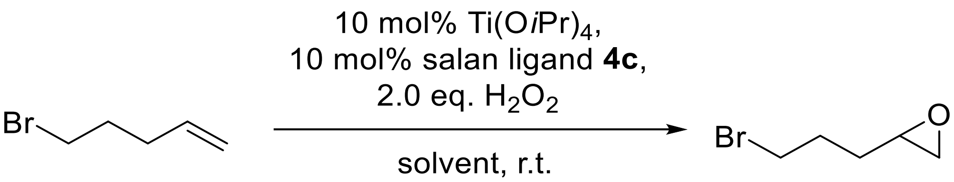

7a **8a**

The reactions were performed according to *General Procedure B*. 10 µmol (10 mol%) Ti(O*i*Pr)$_4$,10 µmol (10 mol%) salan ligand **4c** and 11.3 µL (50 w%, 200 µmol, 2.0 eq.) H_2O_2 were used. The solvent was varied. The conversion-time-profiles are shown in **Figure S3**.

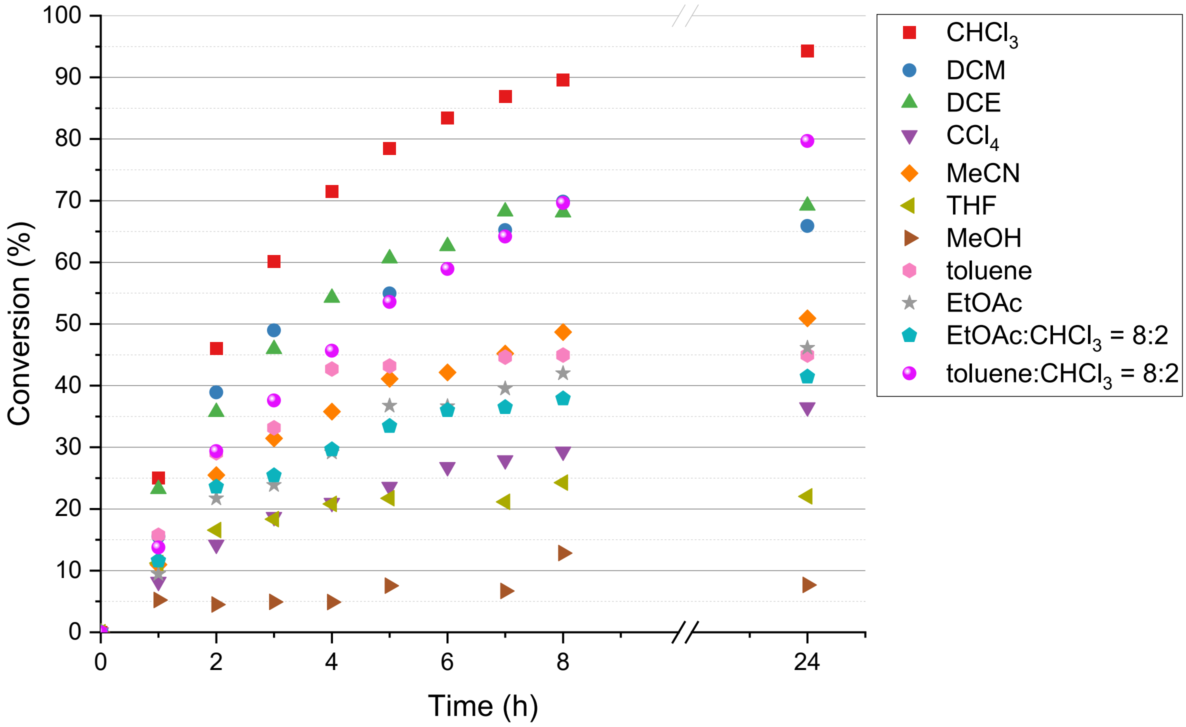

Figure S3: Solvent screening of the epoxidation of 5-bromo-1-pentene (**7a**) with the Ti-complex of the salan ligand **4c** as catalyst.

*Additive Screening of the Ti-catalyzed Epoxidation of 5-Bromo-1-pentene (**7a**) using the Salan Ligand **4c***

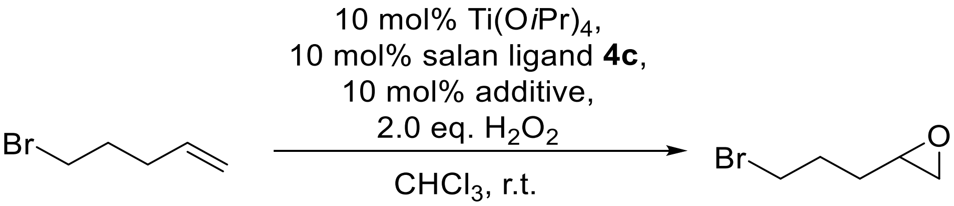

The reactions were performed according to *General Procedure B*. 10 µmol (10 mol%) Ti(O*i*Pr)$_4$,10 µmol (10 mol%) salan ligand **4c**, 11.3 µL (50 w%, 200 µmol, 2.0 eq.) H_2O_2 and 10 µmol (10 mol%) of the corresponding additive were used. The reactions were performed in $CHCl_3$. The conversion-time-profiles are shown in **Figure S4**.

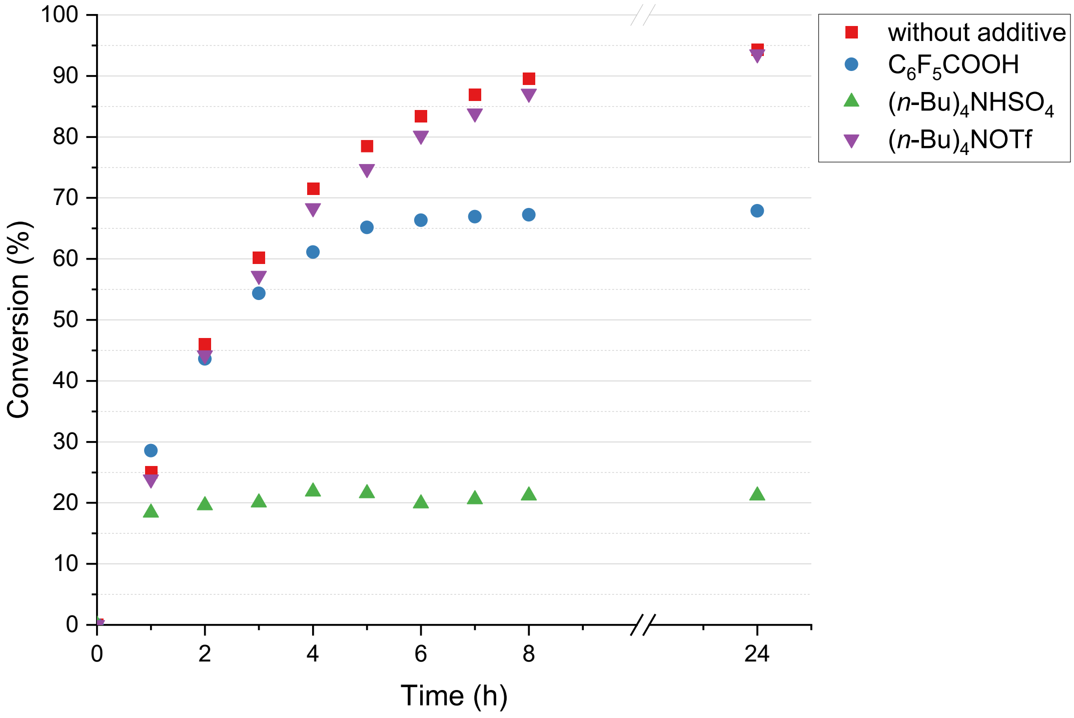

Figure S4: Additive screening of the epoxidation of 5-bromo-1-pentene (**7a**) with the Ti-complex of the salan ligand **4c** as catalyst.

Variation of the Substrate Concentration

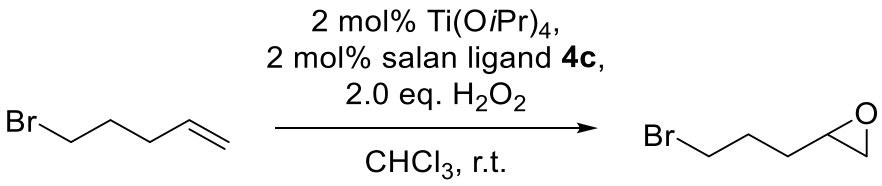

7a **8a**

In a glovebox, a solution of 2 mol% Ti(OiPr)$_4$ in 1 mL absolute DCM was added to 2 mol% of the ligand **4c**. The solution was taken from the glovebox and stirred for 1 h. The solvent was removed under reduced pressure. Afterwards, 5-bromo-1-pentene (**7a**, 1.0 eq.) and bromobenzene (1.0 eq., internal standard) were added. The appropriate amount of $CHCl_3$ was added and the mixture was stirred for 15 min, then H_2O_2 (50 w%, 2.0 eq.) was added. The reaction mixture was stirred at room temperature. The weighted samples and the reaction scale are given in **Table S1**. The reaction progress and enantioselectivity were monitored by chiral GC: A 10 µL aliquot was withdrawn and diluted with *c*-hexane (approx. 0.4 mL). The solution was filtered through a mixture of $MgSO_4$ and MnO_2 (10:1) into a GC-vial with inlay (100 µL volume) and submitted to GC-analysis. A sample was taken before H_2O_2 was added and submitted to GC-analysis as sample for t = 0. The conversion of 5-bromo-1-pentene (**7a**) was determined from the ratio of the integrals between 5-bromo-1-pentene (**7a**) and bromobenzene over time. The conversion-time-profiles are shown in **Figure S5**.

Table S1: Weighted samples and scale for the reactions with varied substrate concentrations.

Entry	Substrate conc.	Ti(O*i*Pr)$_4$	**4c**	**7a**	Bromo-benzene	$CHCl_3$
1	1 mol/L	4.6 mg (16 µmol)	10.6 mg (16 µmol)	126 mg (800 µmol)	125 mg (800 µmol)	550 µL
2	2 mol/L	9.1 mg (32 µmol)	21 mg (32 µmol)	245 mg (1.60 mmol)	238 mg (1.51 mmol)	350 µL
3	3 mol/L	14 mg (48 µmol)	32 mg (48 µmol)	359 mg (2.40 mmol)	373 mg (2.40 µmol)	150 µL

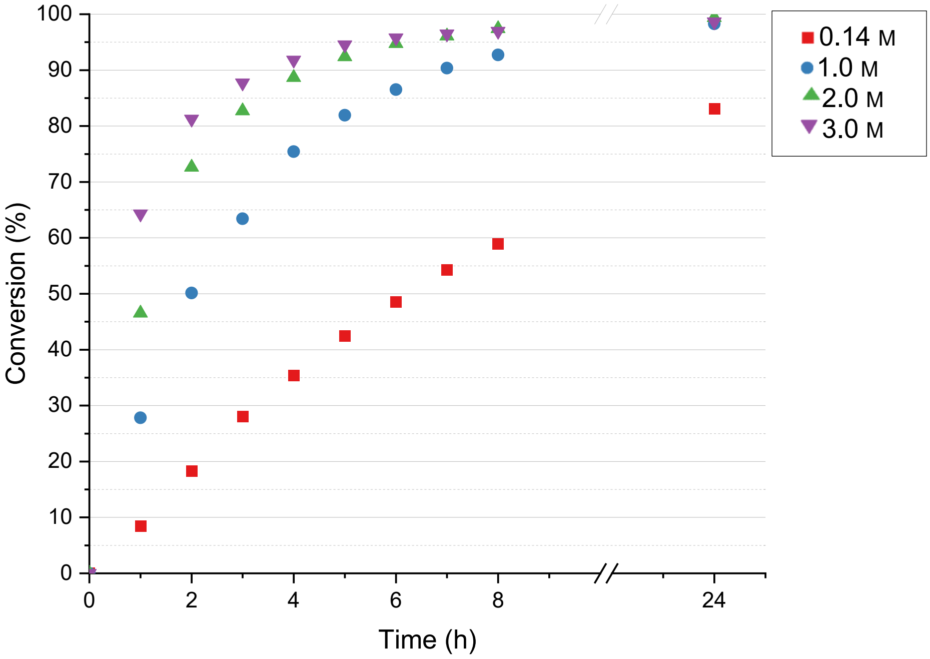

Figure S5: Variation of the substrate concentration in the epoxidation of 5-bromo-1-pentene (**7a**) with the Ti-complex of the salan ligand **4c** as catalyst.

Effect of Sodium Phosphate Buffers at a Substrate Concentration of 2.0 M

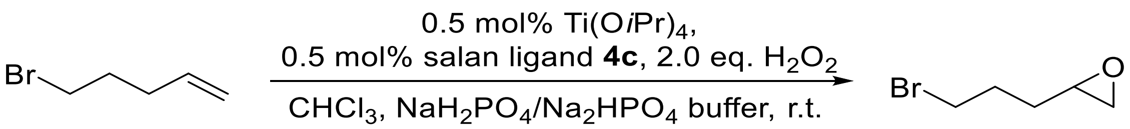

7a **8a**

In a glovebox, a solution of $Ti(O\textit{i}Pr)_4$ (2.3 mg, 8.0 µmol, 0.5 mol%) in 1 mL absolute DCM was added to the salan ligand **4c** (5.3 mg, 8.0 µmol, 0.5 mol%). The solution was taken from the glovebox and stirred for 1 h. The solvent was removed under reduced pressure. Afterwards, 5-bromo-1-pentene (**7a**, 238 mg, 1.60 mmol, 1.0 eq.), bromobenzene (25.1 mg, 160 µmol, 0.1 eq., internal standard) and 500 µL $CHCl_3$ were added. The mixture was stirred for 15 min, then first H_2O_2 (181 µL, 50 w%, 3.20 mmol, 2.0 eq.) and then NaH_2PO_4/Na_2HPO_4 buffer (350 µL, 67 mM) were added. The reaction mixture was stirred at room temperature. The reaction progress and the enantioselectivity were monitored by chiral GC: A 10 µL aliquot was withdrawn and diluted with *c*-hexane (approx. 1.5 mL). The solution was filtered through a mixture of $MgSO_4$ and MnO_2 (10:1) into a GC-vial and submitted to GC-analysis. A sample was taken before H_2O_2 was added and submitted to GC-analysis as sample for t = 0. The conversion of 5-bromo-1-pentene (**7a**) was determined from the ratio of the integrals between 5-bromo-1-pentene (**7a**) and bromobenzene over time. The conversion-time-profile is shown in **Figure S6**.

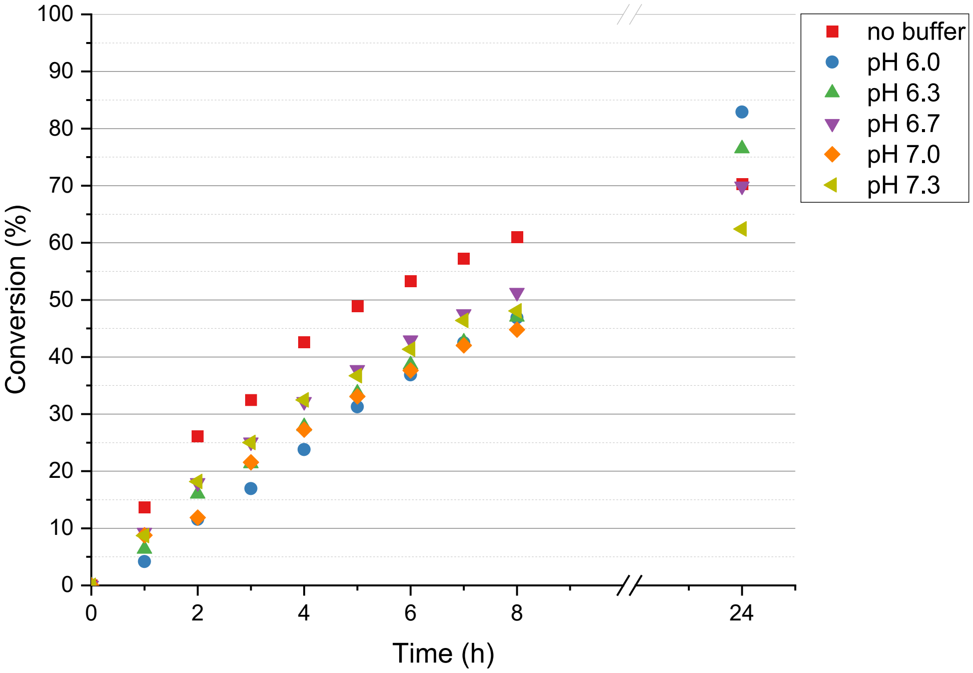

Figure S6: Screening of the addition of NaH_2PO_4/Na_2HPO_4 buffers with different pH-values in the epoxidation of 5-bromo-1-pentene (**7a**) with the Ti-complex of the salan ligand **4c** as catalyst at a substrate concentration of 2.0 M.

1.4 Crystallisation and X-ray Crystal Structure of the oxo-peroxo-Ti Complex of Salan 4c

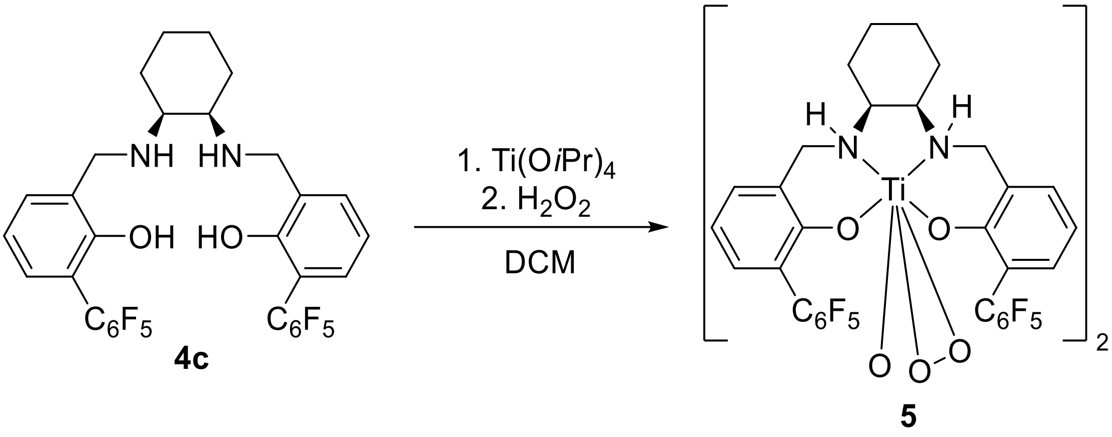

In a glovebox, a solution of 13.1 mg (46.0 µmol, 1.0 eq.) Ti(O*i*Pr)$_4$ in 1 mL abs. DCM was added to 29.5 mg (46.0 µmol, 1.0 eq.) of the salan ligand 4**c**. The reaction was stirred for 1 h, then 10 µL water were added and the mixture stirred for 1 h. Afterwards, 10 µL (50 w%) H_2O_2 were added. The mixture was stirred overnight, then crystallization from different solvents was attempted. Slow evaporation of a solution of the complex **5** in DCM afforded orange crystals suitable for X-ray crystallography (**Figure S7** and **Table S2**).

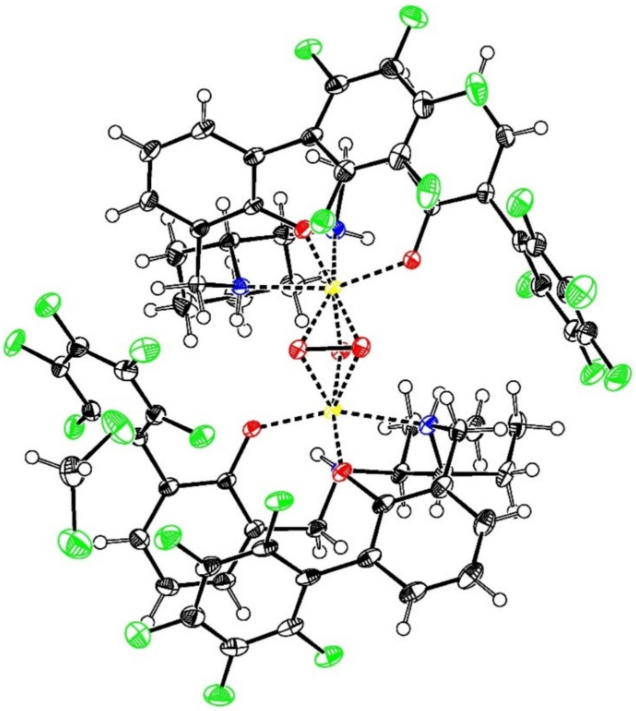

Figure S7: ORTEP diagram of the Ti-complex **ΛΛ-5** at 50% probability (DCM was omitted). The two enantiomers **ΛΛ-5** and **ΔΔ-5** co-crystallized as a racemate (CCDC 2384477).

Table S2: Crystal data and structure refinement for the Ti-complex **5**.

CCDC	2384477	
Empirical formula	C65 H46 Cl2 F20 N4 O7 Ti2	
Moiety formula	C64 H44 F20 N4 O7 Ti2, C H2 Cl2	
Formula weight	1541.76	
Temperature	100(2) K	
Wavelength	1.54178 Å	
Crystal system	Monoclinic	
Space group	$P2_{1/n}$	
Unit cell dimensions	a = 14.7287(13) Å	α = 90°.
	b = 17.8553(15) Å	β = 96.920(4)°.
	c = 24.044(2) Å	γ = 90°.
Volume	6277.2(9) $Å^3$	
Z	4	
Density (calculated)	1.631 Mg/m^3	
Absorption coefficient	4.010 mm^{-1}	
F(000)	3112	
Crystal size	0.300 x 0.150 x 0.050 mm^3	
Theta range for data collection	3.091 to 72.352°.	
Index ranges	-18 ≤ h ≤ 18, -22 ≤ k ≤ 21, -29 ≤ l ≤ 29	
Reflections collected	155046	
Independent reflections	12381 [R(int) = 0.0538]	
Completeness to theta = 67.679°	100.0 %	
Absorption correction	Semi-empirical from equivalents	
Max. and min. transmission	0.7536 and 0.470	
Refinement method	Full-matrix least-squares on F^2	
Data / restraints / parameters	12381 / 0 / 917	
Goodness-of-fit on F2	1.031	
Final R indices [I>2sigma(I)]	R1 = 0.0357, wR2 = 0.0952	
R indices (all data)	R1 = 0.0378, wR2 = 0.0968	
Extinction coefficient	n/a	
Largest diff. peak and hole	0.796 and -0.703 e.$Å^{-3}$	

1.5 Crystallization and X-ray Crystal Structure of the Racemic Ti-Complex of Salalen 2

The crystal structure was obtained by mixing solutions of the Ti-complexes **ΛΛ-6** and **ΔΔ-6** and slow evaporation of this mixture in DCM, following previously reported methods.[2]

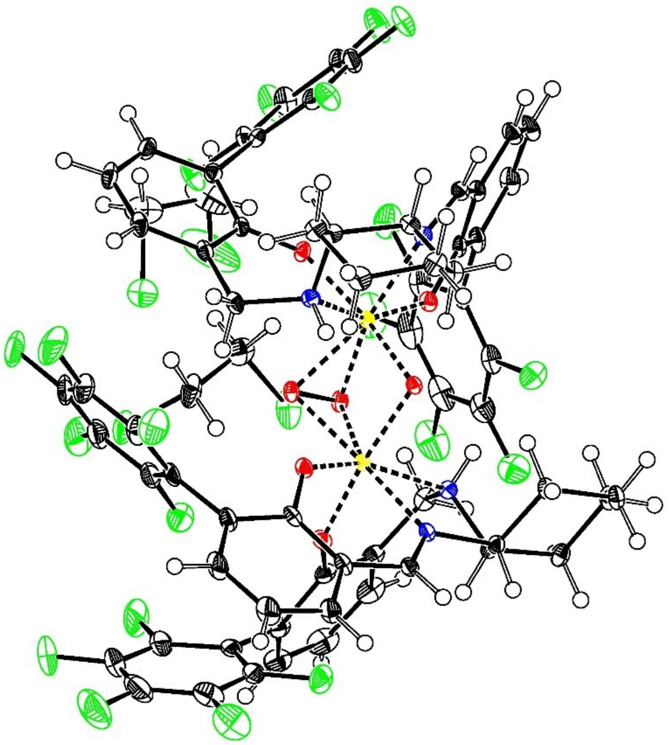

Figure S8: ORTEP diagram of the Ti-complex **ΔΔ-6** at 50% probability (DCM was omitted). The two enantiomers **ΛΛ-6** and **ΔΔ-6** co-crystallized as a racemate (CCDC 2384476). Note that in the enantiomorphic Ti-salalen dimers **ΛΛ-6** and **ΔΔ-6**, the Λ- and Δ-configurated monomeric complexes contain the enantiomeric ligands **2** and ent-**2**. The configuration of the monomeric complexes is dictated by the *configuration* of the *cis*-DACH component. In contrast, in **ΛΛ-5** and **ΔΔ-5**, **Λ-** or **Δ-**configuration of the monomeric complexes corresponds to a specific *conformation* of the *cis*-DACH component.

Table S3: Crystal data and structure refinement for the Ti-complex **6**.

CCDC	2384476
Empirical formula	C68 H48 Cl4 F20 N4 O7 Ti2
Moiety formula	C64 H40 F20 N4 O7 Ti2, 2(C2 H4 Cl2)
Formula weight	1650.70
Temperature	100(2) K
Wavelength	1.54178 Å
Crystal system	Orthorhombic
Space group	Pbca
Unit cell dimensions	a = 25.372(6) Å α = 90°.
	b = 19.445(2) Å β = 90°.
	c = 27.163(4) Å γ = 90°.
Volume	13401(4) $Å^3$
Z	8
Density (calculated)	1.636 Mg/m^3
Absorption coefficient	4.518 mm^{-1}
F(000)	6656
Crystal size	0.400 x 0.200 x 0.200 mm^3
Theta range for data collection	3.254 to 72.284°.
Index ranges	-31 ≤ h ≤ 31, -16 ≤ k ≤ 22, -33 ≤ l ≤ 32
Reflections collected	95292
Independent reflections	13080 [R(int) = 0.0380]
Completeness to theta = 67.679°	99.4 %
Absorption correction	Semi-empirical from equivalents
Max. and min. transmission	.7536 and .4845
Refinement method	Full-matrix least-squares on F^2
Data / restraints / parameters	13080 / 0 / 955
Goodness-of-fit on F2	1.050
Final R indices [I>2sigma(I)]	R1 = 0.0390, wR2 = 0.0995
R indices (all data)	R1 = 0.0407, wR2 = 0.1007
Extinction coefficient	0.000163(11)
Largest diff. peak and hole	0.892 and -1.091 e.$Å^{-3}$

1.6 General Procedure C: Epoxidation with the Ti-Complexes of Salalen 2 or Salan 4c on Preparative Scale

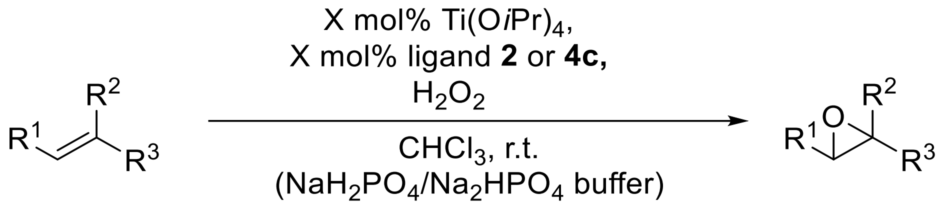

In a glovebox, a solution of Ti(O*i*Pr)$_4$ (X mol%) in 1 mL absolute DCM was added to the ligand (X mol%). The solution was taken from the glovebox and stirred for 1 h. The solvent was removed under reduced pressure. Afterwards, the corresponding olefin (1.60 mmol, 1.0 eq.) and 500 µL $CHCl_3$ were added. The mixture was stirred for 15 min, then first H_2O_2 (50 w%) and then NaH_2PO_4/Na_2HPO_4 buffer (67 mM, if the salan ligand **4c** was used) were added. The reaction mixture was stirred at room temperature for 24 h. The solution was then filtered through a mixture of $MgSO_4$ and MnO_2 (10:1) and the filter cake was washed with DCM. The solvent of the organic phase was removed under reduced pressure, and the residue was purified by column chromatography.

1.7 Epoxidation with the Ti-Complex of Salan 4c on Preparative Scale

*Epoxidation of 5-Bromo-1-pentene (**7a**)*

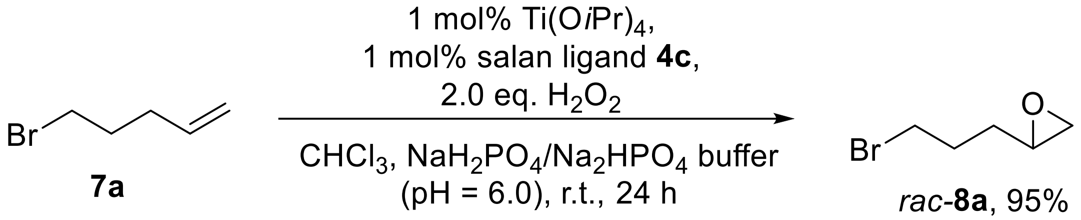

7a *rac*-**8a**, 95%

The reaction was performed according to *General Procedure C*. 4.6 mg (16 µmol, 1 mol%) Ti(O*i*Pr)$_4$ and 10.6 mg (16.0 µmol, 1 mol%) of the salan-ligand **4c**, 250 mg (1.68 mmol, 1.0 eq.) of 5-bromo-1-pentene (**7a**) and 190 µL (50 w%, 3.25 mmol, 2.0 eq.) H_2O_2 were used. 350 µL of NaH_2PO_4/Na_2HPO_4 buffer (67 mm, pH = 6.0) were added. The conversion-time-profile is shown in **Figure S9**. The reaction mixture was submitted directly to column chromatography (pentane:Et_2O = 20:1) with a layer of $MgSO_4$ and MnO_2 (10:1) on top of the silica instead of being filtered through a mixture of $MgSO_4$ and MnO_2 beforehand. 260 mg (1.59 mmol, 95%, containing 9 w% of Et_2O) of the epoxide *rac*-**8a** were obtained as a colourless oil.

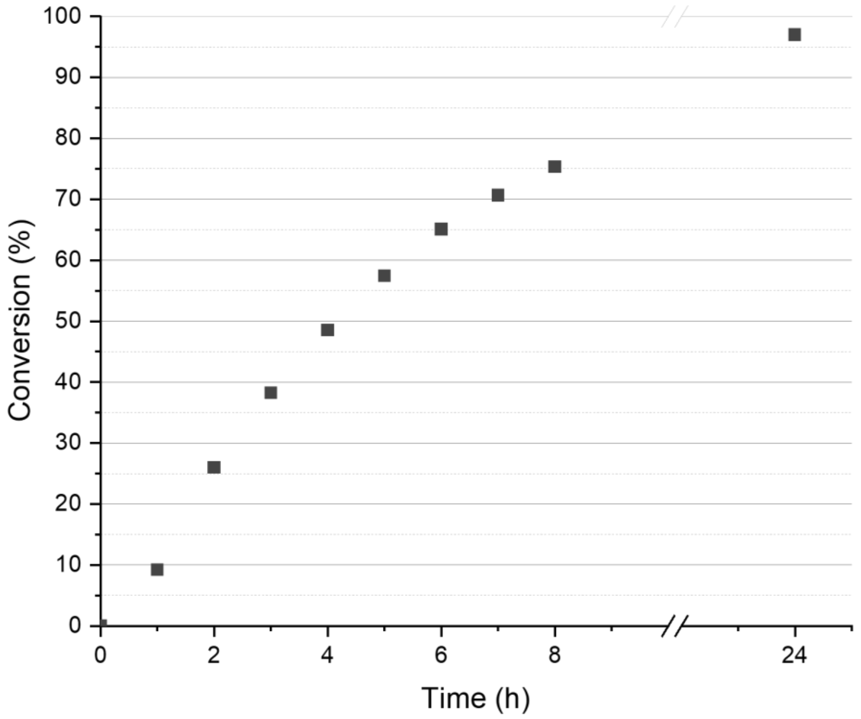

Figure S9: Epoxidation of 5-bromo-1-pentene (**7a**) with the Ti-complex of salan-ligand **4c** as catalyst under optimized conditions: 1 mol% Ti(O*i*Pr)$_4$, 1 mol% of the salan ligand **4c** and 2.0 eq. H_2O_2 at a substrate concentration of 2.0 M with NaH_2PO_4/Na_2HPO_4 buffer (67 mM, pH = 6.0) added.

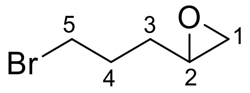

rac-**8a**	M[C_5H_9BrO]: 165.03 g/mol.
**R$_f$**	0.32 (pentane:Et_2O = 4:1).
**^{1}H-NMR**	(500 MHz, $CDCl_3$) δ (ppm) = 3.54 – 3.41 (m, 2H, H-5), 2.97 – 2.92 (m, 1H, H-2), 2.77 (dd, ^2J = 5.0 Hz, ^3J = 4.0 Hz, 1H, H-1a), 2.51 (dd, ^2J = 5.0 Hz, ^3J = 2.7 Hz, 1H, H-1b), 2.12 – 1.97 (m, 2H, H-4), 1.86 – 1.77 (m, 1H, H-3a), 1.64 – 1.55 (m, 1H, H-3b).

**^{13}C-NMR**	(75 MHz, $CDCl_3$) δ (ppm) = 51.4 (1C, C-2), 46.9 (1C, C-1), 33.1 (1C, C-5), 31.0 (1C, C-3), 29.3 (1C, C-4).
**GC-MS**	[injector: 180 °C (split ratio 50:1), oven: 50 °C (5 min), 20 °C/min, 280 °C (10 min)] τ_R = 10.0 min m/z (%) = 137.9 (4) , 106.1 (9), 95.0 (15), 93 (18), 85.1 (100) $[M-Br]^+$, 81.0 (15) $[Br]^+$, 79.0 (19) $[Br]^+$, 67.0 (18), 55.1 (92).
**FT-IR**	(ATR): $\tilde{\nu}$ [cm^{-1}] = 2965 (w), 2924 (w), 1499 (w), 1481 (w), 1437 (m), 1409 (w), 1300 (w), 1254 (s), 1207 (m), 1132 (w), 1045 (w), 991 (m), 916 (s), 858 (vs), 843 (s), 829 (s), 787 (m), 760 (m), 739 (w), 640 (m).

The analytical data are in agreement with the literature.[4]

*Epoxidation of 1-Decene (**7b**)*

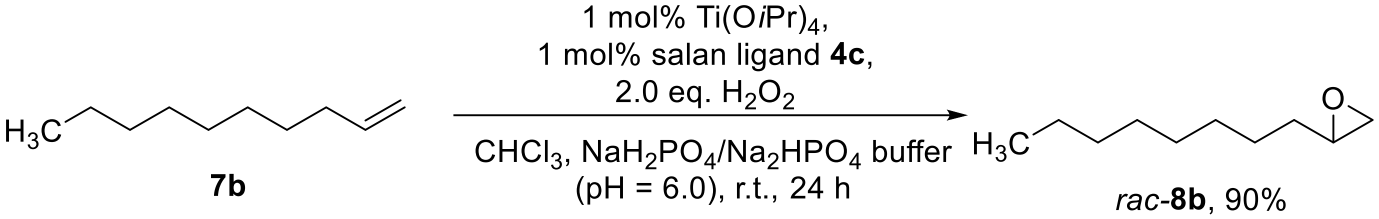

The reaction was performed according to *General Procedure C*. 4.6 mg (16 µmol, 1 mol%) Ti(O*i*Pr)$_4$, 10.7 mg (16.0 µmol, 1 mol%) of the salan ligand **4c**, 228 mg (1.60 mmol, 1.0 eq.) 1-decene (**7b**) and 190 µL (50 w%, 3.25 mmol, 2.0 eq.) H_2O_2 were used. 350 µL of NaH_2PO_4/Na_2HPO_4 buffer (67 mM, pH = 6.0) were added. The reaction mixture was submitted directly to column chromatography (pentane:Et_2O = 20:1) with a layer of $MgSO_4$ and MnO_2 (10:1) on top of the silica instead of being filtered through a mixture of $MgSO_4$ and MnO_2 beforehand. 227 mg (1.45 mmol, 90%) of the epoxide *rac*-**8b** were obtained as a colourless oil.

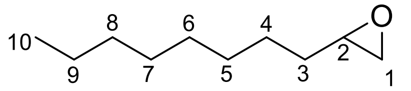

***rac*-8b**	M[$C_{10}H_{20}O$]: 156.27 g/mol.
**R_f**	0.55 (pentane:Et_2O = 10:1).
**^{1}H-NMR**	(500 MHz, $CDCl_3$) δ (ppm) = 2.93 – 2.88 (m, 1H, H-2), 2.76 (dd, ^2J = 5.0, ^3J = 4.0 Hz, 1H, H-1a), 2.47 (dd, ^2J = 5.0, ^3J = 2.7 Hz, 1H, H-1b), 1.57 – 1.12 (m, 14H, H-3, H-4, H-5, H-6, H-7, H-8, H-9), 0.97 – 0.83 (m, 3H, H-10).
**^{13}C-NMR**	(75 MHz, $CDCl_3$) δ (ppm) = 52.4 (1C, C-2), 47.1 (1C, C-1), 32.5 (1C, *CH_2*), 31.8 (1C, *CH_2*), 29.5 (1C, *CH_2*), 29.4 (1C, *CH_2*), 29.2 (1C, *CH_2*), 26.0 (1C, *CH_2*), 22.7 (1C, *CH_2*), 14.1 (1C, C-10).
**GC-MS**	[injector: 180 °C (split ratio 50:1), oven: 50 °C (5 min), 20 °C/min, 280 °C (10 min)] τ_R = 9.4 min m/z (%) = 138.1 (4), 109.2 (6), 95.1 (24), 81.1 (37), 71.1 (78), 55.1 (100)
**FT-IR**	(ATR): $\tilde{\nu}$ [cm^{-1}] = 2955 (m), 2922 (s), 2855 (m), 1458 (m), 1410 (w), 1259 (w), 916 (w), 904 (w), 833 (m), 723 (w).

The analytical data are in agreement with the literature.[2]

*Epoxidation of Vinylcyclohexane (**7c**)*

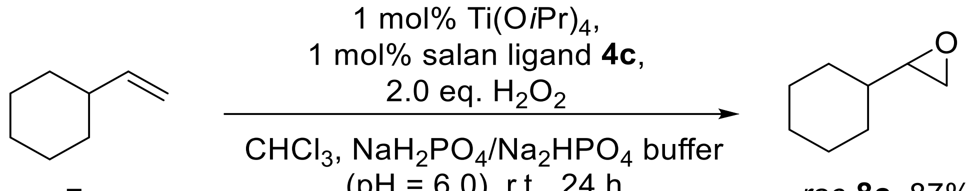

7c *rac*-**8c**, 87%

The reaction was performed according to *General Procedure C*. 4.6 mg (16 µmol, 1 mol%) Ti(O*i*Pr)$_4$, 10.4 mg (16.0 µmol, 1 mol%) of the salan ligand **4c**, 176 mg (1.60 mmol, 1.0 eq.) vinylcyclohexane (**7c**) and 182 µL (50 w%, 3.20 mmol, 2.0 eq.) H_2O_2 were used. 350 µL of NaH_2PO_4/Na_2HPO_4 buffer (67 mM, pH = 6.0) were added. The reaction mixture was submitted directly to column chromatography (pentane:Et_2O = 20:1) with a layer of $MgSO_4$ and MnO_2 (10:1) on top of the silica instead of being filtered through a mixture of $MgSO_4$ and MnO_2 beforehand. 200 mg (1.39 mmol, 87%, containing 12 w% of Et_2O) of the epoxide *rac*-**8c** were obtained as a colourless oil.

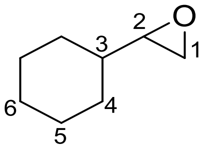

rac-**8c**	M[$C_8H_{14}O$]: 126.20 g/mol.
**R_f**	0.33 (pentane:Et_2O = 20:1).
**^{1}H-NMR**	(500 MHz, $CDCl_3$) δ (ppm) = 2.74 – 2.68 (m, 2H, H-1a, H-2), 2.52 (dd, ^2J = 4.5 Hz, ^3J = 3.3 Hz, 1H, H-1b), 1.90 – 1.85 (m, 1H, H-4a), 1.78 – 1.72 (m, 2H, H-5a, H-5b), 1.70 – 1.64 (m, 2H, H-4b, H-5c), 1.33 – 1.06 (m, 6H, H-3, H-4c, H-4d, H-5d, H-6).
**^{13}C-NMR**	(75 MHz, $CDCl_3$) δ (ppm) = 56.7 (1C, C-2), 46.0 (1C, C-1), 40.4 (1C, C-3), 29.7 (1C, C-4a), 28.8 (1C, C-4b), 26.3 (1C, C-5a), 25.7 (1C, C-5b/6), 25.5 (1C, C-6/5b).
**GC-MS**	[injector: 180 °C (split ratio 50:1), oven: 50 °C (5 min), 20 °C/min, 280 °C (10 min)] τ_R = 7.8 min m/z (%) = 111.2 (5) [M-CH_3]$^+$, 96.0 (19) [M-CH_2O]$^+$, 93.0 (13), 90.9 (13), 81.1 (100), 79.1 (30), 77.1 (13), 67.1 (60), 65.2 (16), 55.1 (36), 54.1 (32), 53.1 (32), 51.1 (21).
**FT-IR**	(ATR): $\tilde{\nu}$ [cm^{-1}] = 2992 (w), 2922 (vs), 2850 (s), 1483 (w), 1448 (m), 1418 (w), 1254 (w), 1231 (w), 1130 (w), 980 (w), 943 (m), 880 (m), 858 (vs), 837 (s), 800 (m), 785 (w).

The analytical data are in agreement with the literature.[5]

*Epoxidation of Styrene (**7d**)*

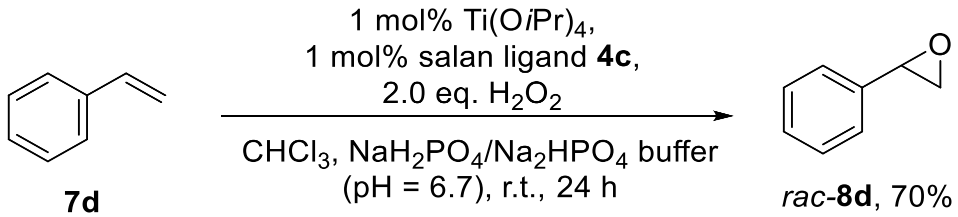

7d *rac*-**8d**, 70%

The reaction was performed according to *General Procedure C*. 4.6 mg (16 µmol, 1 mol%) Ti(O*i*Pr)$_4$, 10.6 mg (16.0 µmol, 1 mol%) of the salan ligand **4c**, 174 mg (1.66 mmol, 1.0 eq.) styrene (**7d**) and 182 µL (50 w%, 3.20 mmol, 2.0 eq.) H_2O_2 were used. 350 µL of the NaH_2PO_4/Na_2HPO_4 buffer (67 mM, pH = 6.7 instead of pH = 6.0) were added. The crude product was purified by column chromatography (pentane:Et_2O = 20:1). 139 mg (1.16 mmol, 70%) of the epoxide *rac*-**8d** were obtained as a colourless oil.

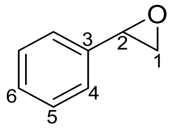

rac-**8d**	M[C_8H_8O]: 120.2 g/mol.
**R_f**	0.36 (pentane:Et_2O = 20:1).

**^{1}H-NMR**	(500 MHz, $CDCl_3$) δ (ppm) = 7.37 – 7.24 (m, 5H, H-4, H-5, H-6), 3.85 (dd, ^3J = 4.1 Hz, ^3J = 2.5 Hz, 1H, H-2), 3.13 (dd, ^2J = 5.5 Hz, ^3J = 4.1 Hz, 1H, H-1a), 2.79 (dd, ^2J = 5.5 Hz, ^3J = 2.6 Hz, 1H, H-1b).
**^{13}C-NMR**	(75 MHz, $CDCl_3$) δ (ppm) = 137.6 (1C, C-3), 128.5 (2C, C-5), 128.2 (1C, C-6), 125.5 (2C, C-4), 52.4 (1C, C-2), 51.2 (1C, C-1).
**GC-MS**	[STAND40] τ_R = 8.7 min, m/z (%) = 119.1 (25) $[M-H]^+$, 104.1 (25) $[M-O]^+$, 91.1 (100) $[M-COH]^+$, 78.0 (17), 63.1 (33), 51.0 (27).
**FT-IR**	(ATR): $\tilde{\nu}$ [cm^{-1}] = 3034 (w), 2988 (w), 1496 (w), 1475 (w), 1389 (w), 1251 (w), 1201 (w), 1127 (w), 1072 (w), 1026 (w), 984 (w), 873 (s), 756 (s), 812 (w), 756 (s), 696 (s).

The analytical data are in agreement with the literature.[6]

*Epoxidation of cis-2-Octene (**7e**)*

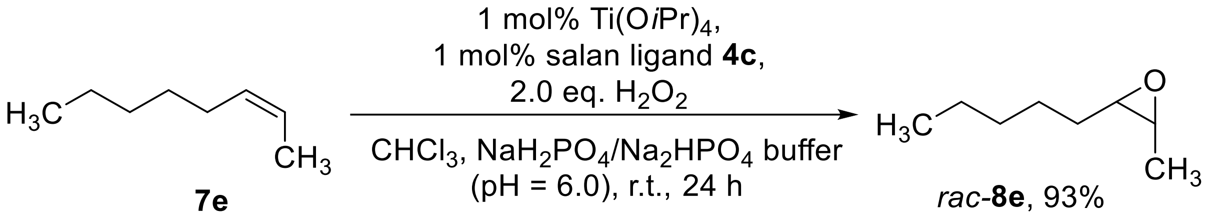

The reaction was performed according to *General Procedure C*. 4.6 mg (16 µmol, 1 mol%) Ti(O*i*Pr)$_4$, 10.6 mg (16.0 µmol, 1 mol%) of the salan ligand **4c**, 185 mg (1.62 mmol, 1.0 eq.) *cis*-2-octene (**7e**) and 182 µL (50 w%, 3.20 mmol, 2.0 eq.) H_2O_2 were used. 350 µL of NaH_2PO_4/Na_2HPO_4 buffer (67 mM, pH = 6.0) were added. The reaction mixture was submitted directly to column chromatography (pentane:Et_2O = 20:1) with a layer of $MgSO_4$ and MnO_2 (10:1) on top of the silica instead of being filtered through a mixture of $MgSO_4$ and MnO_2 beforehand. 215 mg (1.52 mmol, 93%, containing 9 w% of Et_2O) of the epoxide *rac*-**8e** were obtained as a colourless oil.

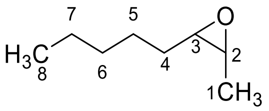

***rac*-8e**	M[$C_8H_{16}O$]: 128.2 g/mol.
**R_f**	0.25 (pentane:Et_2O = 20:1).
**^{1}H-NMR**	(500 MHz, $CDCl_3$) δ (ppm) = 3.04 (qd, ^3J = 5.5 Hz, ^3J = 4.2 Hz, 1H, H-2), 2.93 – 2.86 (m, 1H, H-3), 1.60 – 1.40 (m, 4H, H-4, H-5), 1.38 – 1.30 (m, 4H, H-6, H-7), 1.26 (d, ^3J = 5.5 Hz, 3H, H-1), 0.97 – 0.83 (m, 3H, H-8).
**^{13}C-NMR**	(75 MHz, $CDCl_3$) δ (ppm) = 57.2 (1C, C-3), 52.6 (1C, C-2), 31.7 (1C, C-6), 27.5 (1C, C-4), 26.1 (1C, C-5), 22.6 (1C, C-7), 14.0 (1C, C-8), 13.2 (1C, C-1).
**GC-MS**	[STAND40] τ_R = 7.7 min, m/z (%) = 128.1 (2) $[M]^+$, 112.2 (6) $[M-O]^+$, 99.0 (7), 85.1 (41), 71.1 (22), 56.1 (100).
**FT-IR**	(ATR): $\tilde{\nu}$ [cm^{-1}] = 2995 (w), 2957 (s), 2926 (s), 2858 (m), 1456 (m), 1391 (m), 1271 (m), 1150 (w), 1117 (w), 1062 (w), 984 (w), 903 (w), 891 (w), 841 (w), 816 (m), 787 (m), 760 (m), 733 (m).

The analytical data are in agreement with the literature.[7]

*Epoxidation of 1,2-Dihydronaphthalene (**7f**)*

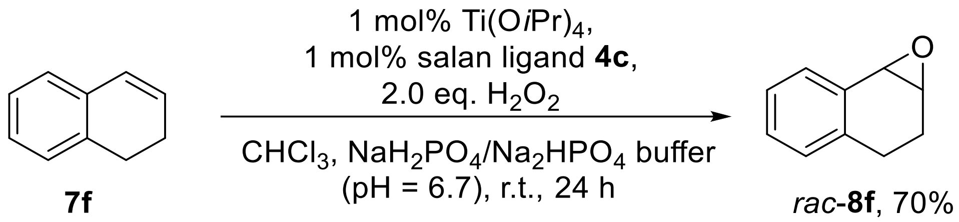

The reaction was performed according to *General Procedure C*. 4.6 mg (16 µmol, 1 mol%) Ti(O*i*Pr)$_4$, 10.7 mg (16.0 µmol, 1 mol%) of the salan ligand **4c**, 214 mg (1.66 mmol, 1.0 eq.) 1,2-dihyhdronaphthalene (**7f**) and 182 µL (50 w%, 3.20 mmol, 2.0 eq.) H_2O_2 were used. 350 µL of the NaH_2PO_4/Na_2HPO_4 buffer (67 mM, pH = 6.7) were added. The crude product was purified by column chromatography (pentane:Et_2O = 20:1). 170 mg (1.16 mmol, 70%) of the epoxide *rac*-**8f** were obtained as a pale-yellow oil.

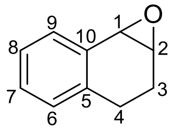

rac-**8f**	M[$C_{10}H_{10}O$]: 146.2 g/mol.
**R$_f$**	0.23 (pentane:Et_2O = 20:1).
**^{1}H-NMR**	(500 MHz, $CDCl_3$) δ (ppm) = 7.38 (dd, ^3J = 7.4 Hz, ^4J = 1.5 Hz, 1H, H-9), 7.25 (Ψ-td, ^3J = 7.4 Hz, ^4J = 1.5 Hz, 1H, H-7), 7.22 – 7.17 (m, 1H, H-8), 7.10 – 7.06 (m, 1H, H-6), 3.83 (d, ^3J = 4.1 Hz, 1H, H-1), 3.74 – 3.70 (m, 1H, H-2), 2.84 – 2.72 (m, 1H, H-4a), 2.54 (dd, ^2J = 15.5 Hz, ^3J = 5.6 Hz, 1H, H-4b), 2.43 – 2.37 (m, 1H, H-3a), 1.83 – 1.68 (m, 1H, H-3b).
**^{13}C-NMR**	(75 MHz, $CDCl_3$) δ (ppm) = 136.7 (1C, C-5), 132.6 (1C, C-10), 130.0 (1C, C-9), 128.4 (1C, C-7), 128.4 (1C, C-8), 126.1 (1C, C-6), 55.1 (1C, C-2), 52.8 (1C, C-1), 24.4 (1C, C-4), 21.8 (1C, C-3).
**GC-MS**	[STAND50] τ_R = 10.5 min, m/z (%) = 146.1 (85) [M]$^+$, 128.1 (68), 115.1 (100), 104.1 (80) [M-C_2H_2O]$^+$, 91.1 (53), 78.0 (29), 63.1 (58), 51.0 (46).
**FT-IR**	(ATR): $\tilde{\nu}$ [cm^{-1}] = 2997 (w), 2933 (w), 2846 (w), 1491 (w), 1466 (w), 1433 (w), 1277 (w), 1248 (w), 1200 (w), 1175 (w), 1059 (w), 1034 (w), 980 (w), 935 (m), 893 (m), 851 (m), 795 (s), 758 (s), 744 (s), 727 (s), 642 (m), 606 (m).

The analytical data are in agreement with the literature.[8]

*Epoxidation of trans-2-Octene (**7g**)*

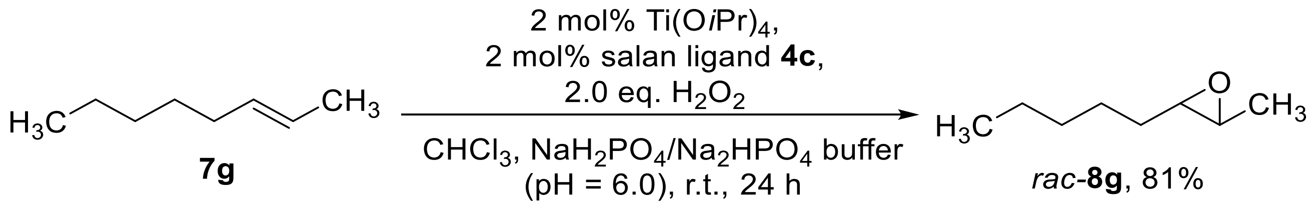

The reaction was performed according to *General Procedure C*. 9.1 mg (32 µmol, 2 mol%) Ti(O*i*Pr)$_4$, 21.1 mg (32.0 µmol, 2 mol%) of the salan ligand **4c**, 179 mg (1.60 mmol, 1.0 eq.) *trans*-2-octene (**7g**) and 182 µL (50 w%, 3.20 mmol, 2.0 eq.) H_2O_2 were used. 350 µL of NaH_2PO_4/Na_2HPO_4 buffer (67 mm, pH = 6.0) were added. The reaction mixture was submitted directly to column chromatography (pentane:Et_2O = 20:1) with a layer of $MgSO_4$ and MnO_2 (10:1) on top of the silica instead of being filtered through a mixture of $MgSO_4$ and MnO_2 beforehand. 180 mg (1.30 mmol, 81%, containing 9 w% of Et_2O) of the epoxide *rac*-**8g** were obtained as a colourless oil.

rac-**8g**	M[$C_8H_{16}O$]: 128.2 g/mol.	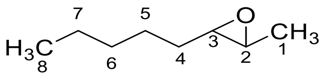
**R$_f$**	0.51 (pentane:Et_2O = 20:1).	

^{1}H-NMR (500 MHz, $CDCl_3$) δ (ppm) = 2.74 (qd, ^3J = 5.2 Hz, ^3J = 2.3 Hz, 1H, H-2), 2.62 (td, ^3J = 5.6 Hz, ^3J = 2.3 Hz, 1H, H-3), 1.57 – 1.38 (m, 4H, H-4, H-7), 1.36 – 1.26 (m, 7H, H-1, H-5, H-6), 0.93 – 0.84 (m, 3H, H-8).

^{13}C-NMR (75 MHz, $CDCl_3$) δ (ppm) = 59.8 (1C, C-3), 54.6 (1C, C-2), 32.0 (1C, C-4), 31.6 (1C, C-5), 25.6 (1C, C-6) 22.6 (1C, C-7), 17.7 (1C, C-1), 14.0 (1C, C-8).

GC-MS [STAND40] $τ_R$ = 7.3 min, m/z (%) = 112.1 (8) [M-O]$^+$, 99.0 (7), 85.1 (35), 71.1 (37), 55.1 (100).

FT-IR (ATR): $\tilde{\nu}$ [cm^{-1}] = 2959 (s), 2928 (s), 2859 (m), 1456 (m), 1379 (m), 1120 (m), 1010 (w), 908 (w), 856 (s), 808 (w), 754 (w), 729 (m), 717 (m).

The analytical data are in agreement with the literature.[9]

*Epoxidation of (1-Methylvinyl)-cyclohexane (**7h**)*

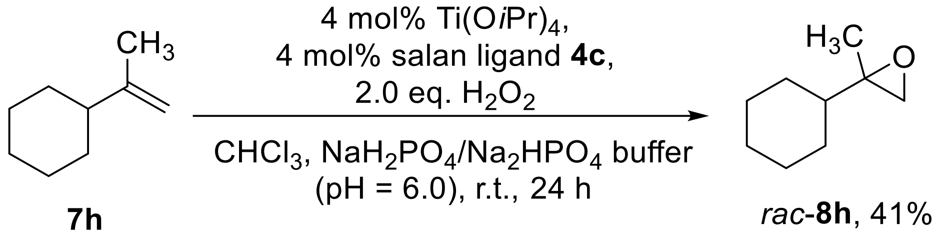

7h *rac*-**8h**, 41%

The reaction was performed according to *General Procedure C*. 18.3 mg (64 µmol, 4 mol%) Ti(O*i*Pr)$_4$, 42.3 mg (64.0 µmol, 4 mol%) of the salan ligand **4c**, 200 mg (1.60 mmol, 1.0 eq.) of olefin **7h** and 182 µL (50 w%, 3.20 mmol, 2.0 eq.) H_2O_2 were used. 350 µL of NaH_2PO_4/Na_2HPO_4 buffer (67 mm, pH = 6.0) were added. The reaction mixture was submitted directly to column chromatography (pentane:Et_2O = 20:1) with a layer of $MgSO_4$ and MnO_2 (10:1) on top of the silica instead of being filtered through a mixture of $MgSO_4$ and MnO_2 beforehand. 120 mg (0.66 mmol, 41%, containing 23 w% of Et_2O) of the epoxide *rac*-**8h** were obtained as a colourless oil.

rac-8h	M[$C_9H_{16}O$]: 140.23 g/mol.	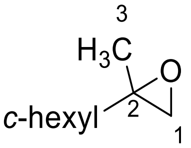
**R$_f$**	0.34 (pentane:Et_2O = 20:1).	

^{1}H-NMR (500 MHz, $CDCl_3$) δ (ppm) = 2.62 (d, ^2J = 4.6 Hz, 1H, H-1a), 2.54 (d, ^2J = 4.6 Hz, 1H, H-1b), 1.85 – 1.65 (m, 5H, *c*-hexyl), 1.27 – 1.00 (m, 9H, H-3, *c*-hexyl).

^{13}C-NMR (75 MHz, $CDCl_3$) δ (ppm) = 59.9 (1C, C-2), 53.6 (1C, C-1), 44.5 (1C, CH-*c*-hexyl), 29.0 (1C, *c*-hexyl), 28.5 (1C, *c*-Hex), 26.4 (1C, *c*-hexyl), 26.3 (1C, *c*-hexyl), 26.1 (1C, *c*-hexyl), 18.0 (1C, C-3).

GC-MS [injector: 180 °C (split ratio 50:1), oven: 50 °C (5 min), 20 °C/min, 280 °C (10 min)] $τ_R$ = 8.4 min m/z (%) = 140.1 (6) [M]$^+$, 125.1 (100) [M-CH_3]$^+$, 111.1 (36), 93.1 (29), 85.1 (21), 81.1 (71), 67.1 (83), 55.1 (45).

FT-IR (ATR): $\tilde{\nu}$ [cm^{-1}] = 2924.1 (s), 2852.7 (m), 1448.5 (m), 1379.1 (w), 1344.4 (w), 1261.5 (w), 1107.1 (w), 1066.6 (w), 904.6 (m), 881.5 (m), 844.8 (m), 819.8 (s), 783.1 (m), 729.9 (w).

The analytical data are in agreement with the literature.[10]

*Epoxidation of 2-Methyl-2-octene (**7i**)*

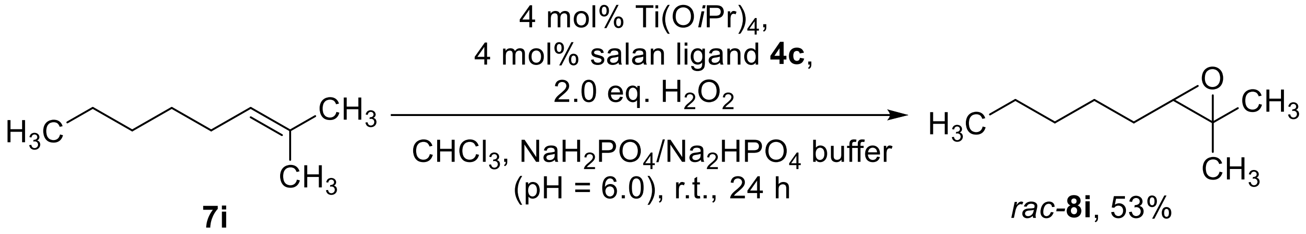

The reaction was performed according to *General Procedure C*. 18.3 mg (64 µmol, 4 mol%) Ti(O*i*Pr)$_4$, 42.3 mg (64.0 µmol, 4 mol%) of the salan ligand **4c**, 201 mg (1.60 mmol, 1.0 eq.) of 2-methyl-2-octene (**7i**) and 182 µL (50 w%, 3.20 mmol, 2.0 eq.) H_2O_2 were used. 350 µL of NaH_2PO_4/Na_2HPO_4 buffer (67 mM, pH = 6.0) were added. The reaction mixture was submitted directly to column chromatography (pentane:Et_2O = 20:1) with a layer of $MgSO_4$ and MnO_2 (10:1) on top of the silica instead of being filtered through a mixture of $MgSO_4$ and MnO_2 beforehand. 135 mg (0.844 mmol, 53%, containing 11 w% of Et_2O) of the epoxide *rac*-**8i** were obtained as a colourless oil.

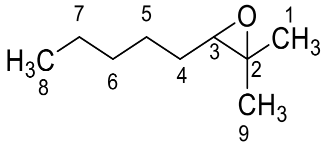

rac-**8i**	M[$C_9H_{18}O$]: 142.2 g/mol.
**R$_f$**	0.42 (pentane:Et_2O = 20:1).
**^{1}H-NMR**	(500 MHz, $CDCl_3$) δ (ppm) = 2.74 – 2.68 (m, 1H, H-3), 1.58 – 1.32 (m, 8H, H-4, H-5, H-6, H-7), 1.31 (s, 3H, H-1/H-9), 1.26 (s, 3H, H-9/H-1), 0.98 – 0.86 (m, 3H, H-8).
**^{13}C-NMR**	(75 MHz, $CDCl_3$) δ (ppm) = 64.6 (1C, C-3), 58.2 (1C, C-2), 31.7 (1C, C-6), 28.8 (1C, C-4), 26.2 (1C, C-5), 24.9 (1C, C-1/C-9), 22.6 (1C, C-7), 18.7 (1C, C-9/C-1), 14.0 (1C, C-8).
**GC-MS**	[STAND40] τ_R = 8.0 min, m/z (%) = 142.0 (3) [M]$^+$, 126.1 (4) [M-O]$^+$, 99.1 (12), 85.1 (31), 71.1 (32), 59,1 (100).
**FT-IR**	(ATR): $\tilde{\nu}$ [cm^{-1}] = 2957 (s), 2924 (s), 2858 (m), 1460 (m), 1377 (s), 1248 (w), 1120 (m), 1016 (w), 881 (m), 837 (w), 793 (w), 681 (m).

The analytical data are in agreement with the literature.[11]

*Epoxidation of 1-Phenylcyclohexene (**7j**)*

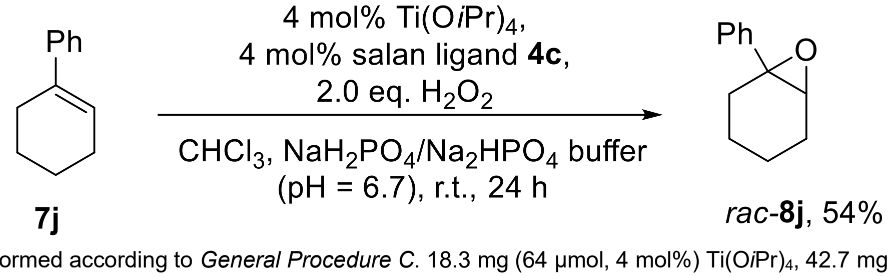

The reaction was performed according to *General Procedure C*. 18.3 mg (64 µmol, 4 mol%) Ti(O*i*Pr)$_4$, 42.7 mg (64.0 µmol, 4 mol%) of the salan ligand **4c**, 254 mg (1.60 mmol, 1.0 eq.) 1-phenylcyclohexene (**7j**) and 182 µL (50 w%, 3.20 mmol, 2.0 eq.) H_2O_2 were used. 350 µL of NaH_2PO_4/Na_2HPO_4 buffer (67 mM, pH = 6.7) were added. The crude product was purified by column chromatography (pentane:Et_2O = 20:1). 150 mg (0.86 mmol, 54%) of the epoxide *rac*-**8j** (relative configuration: *cis*) were obtained as a colorless oil.

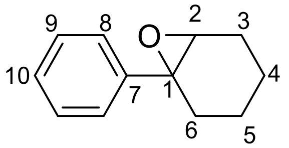

rac-**8j**	M[$C_{12}H_{14}O$]: 174.24 g/mol.
**R$_f$**	0.39 (pentane:Et_2O = 30:1).
**^{1}H-NMR**	(500 MHz, $CDCl_3$) δ (ppm) = 7.39 – 7.36 (m, 2H, H-8), 7.35 – 7.31 (m, 2H, H-9), 7.27 – 7.23 (m, 1H, H-10), 3.08 – 3.06 (m, 1H, H-2), 2.32 – 2.24 (m, 1H, H-6a), 2.16 – 2.08 (m, 1H, H-6b), 2.06 –

	1.93 (m, 2H, H-3), 1.66 – 1.52 (m, 2H, H-4/5), 1.52 – 1.41 (m, 1H, H-5a/4a), 1.38 – 1.27 (m, 1H, H-5b/4b).
**^{13}C-NMR**	(75 MHz, $CDCl_3$) δ (ppm) = 142.5 (1C, C-7), 128.3 (2C, C-9), 127.2 (1C, C-10), 125.3 (2C, C-8), 61.9 (1C, C-2), 60.2 (1C, C-1), 28.9 (1C, C-6), 24.7 (1C, C-3), 20.1 (1C, C-4/5), 19.8 (1C, C-5/4).
**GC-MS**	[injector: 180 °C (split ratio 50:1), oven: 50 °C (5 min), 20 °C/min, 280 °C (10 min)] τ_R = 10.0 min m/z (%) = 173.1 (52) $[M-H]^+$, 159.1 (15), 145.1 (26) $[M-CHO]^+$, 129.1 (56), 115.1 (100), 105.2 (41), 91.1 (74), 77.1 (78) $[Ph]^+$, 63.1 (27), 51.1 (46),
**FT-IR**	(ATR): $\tilde{\nu}$ [cm^{-1}] = 2936 (m), 2916 (m), 2859 (w), 1495 (m), 1446 (m), 1418 (w), 1296 (w), 1247 (w), 1172 (w), 1132 (w), 1078 (w), 1030 (w), 993 (m), 974 (s), 928 (w), 906 (m), 871 (m), 852 (s), 823 (m), 773 (s), 748 (vs), 696 (vs), 681 (m), 635 (m), 581 (m).

The analytical data are in agreement with the literature.[8]

*Epoxidation of α-Vinylbenzyl Alcohol (rac-**7k**)*

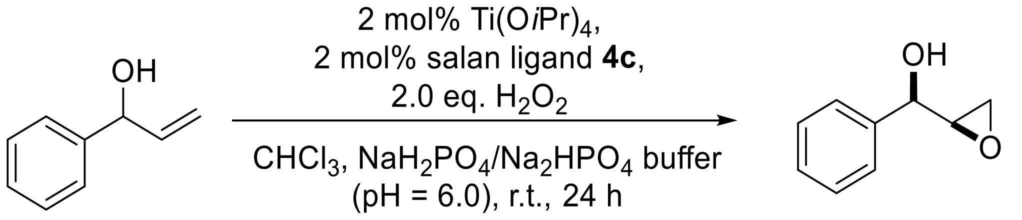

rac-**7k** *rac*-**8k**, 91%, 4.7:1 *dr (syn:anti)*

The reaction was performed according to *General Procedure C*. 9.1 mg (32 µmol, 2 mol%) $Ti(OiPr)_4$, 21.0 mg (32.0 µmol, 2 mol%) of the salan ligand **4c**, 216 mg (1.60 mmol, 1.0 eq.) of racemic α-vinylbenzylalcohol (*rac*-**7k**) and 182 µL (50 w%, 3.20 mmol, 2.0 eq.) H_2O_2 were used. 350 µL of NaH_2PO_4/Na_2HPO_4 buffer (67 mm, pH = 6.0) were added. The crude product was purified by column chromatography (*c*-Hex:EtOAc = 4:1) to give the product in a yield of 222 mg (1.46 mmol, 91%). The mixture of stereoisomeric epoxides was obtained as a colorless liquid with a ratio of the *syn*/*anti*-racemates of 4.7:1 (determined by ^{1}H-NMR).

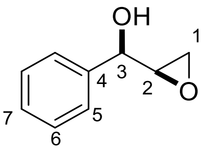

rac-**8k**	M[$C_9H_{10}O$]: 150.2 g/mol.
**R_f**	0.13 (*c*-Hex:EtOAc = 4:1).
**^{1}H-NMR**	(500 MHz, $CDCl_3$) δ (ppm) = 7.54 – 7.32 (m, 5H, H-5, H-6, H-7), 4.95 (d, ^3J = 3.0 Hz, 1H, H-3$_{anti}$), 4.57 – 4.41 (m, 1H, H-3$_{syn}$), 3.28 – 3.22 (m, 1H, H-2), 2.99 (dd, ^2J = 5.0 Hz, ^3J = 2.8 Hz, 1H, H-1a$_{anti}$), 2.91 – 2.82 (m, 2H, H-1$_{syn}$), 2.78 (dd, ^2J = 5.0 Hz, ^3J = 4.0 Hz, 1H, H-1b$_{anti}$), 2.67 (d, ^3J = 4.5 Hz, 1H, O*H*$_{syn}$), 2.48 – 2.41 (m, 1H, O*H*$_{anti}$).
**^{13}C-NMR**	(75 MHz, $CDCl_3$) δ (ppm) = 140.1 (1C, C-4$_{syn}$), 139.4 (1C, C-4$_{anti}$), 128.7 (2C, C-6$_{syn}$), 128.6 (2C, C-6$_{anti}$), 128.3 (1C, C-7$_{anti}$), 128.2 (1C, C-7$_{syn}$), 126.4 (2C, C-5$_{anti}$), 126.3 (2C, C-5$_{syn}$), 74.5 (1C, C-3$_{syn}$), 70.8 (1C, C-3$_{anti}$), 56.0 (1C, C-2$_{syn}$), 55.1 (1C, C-2$_{anti}$), 45.4 (1C, C-1$_{syn}$), 43.6 (1C, C-1$_{anti}$).
**GC-MS**	[STAND50] τ_R = 10.4 min, m/z (%) = 150.1 (6) $[M]^+$, 117.1 (8), 107.1 (100) $[M-C_2H_3O]^+$, 91.1 (44), 79.1 (92), 65.1 (16), 51.1 (37).
**FT-IR**	(ATR): $\tilde{\nu}$ [cm^{-1}] = 3435 (b), 3062 (w), 2997 (w), 1382 (w), 1254 (w), 1196 (w), 1040 (m), 1024 (m), 926 (m), 912 (m), 741 (s), 698 (s).

The analytical data are in agreement with the literature.[12]

1.8 Epoxidation of Terpenes with the Ti-Complexes of Salan 4c or Salalen 2

*Epoxidation of Myrcene (**9**)*

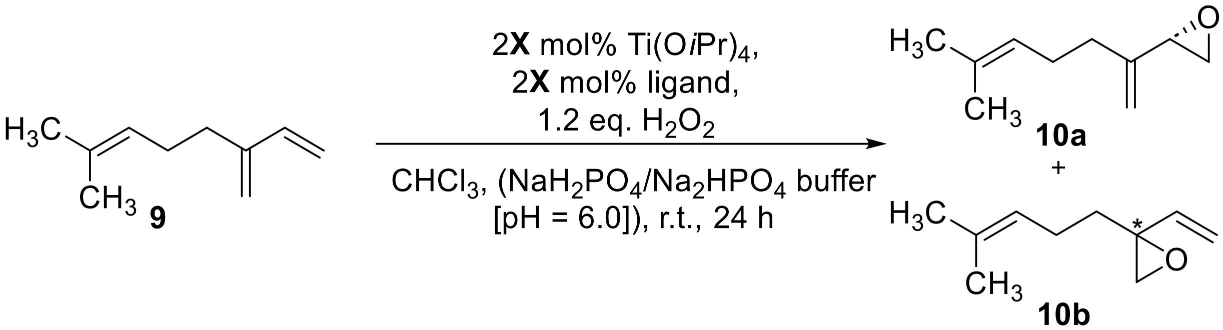

The reactions were performed according to *General Procedure C* on a 1.00 µmol scale. 68 µL (50 w%, 1.20 mmol, 1.2 eq.) H_2O_2 were added. 130 µL of NaH_2PO_4/Na_2HPO_4 buffer (67 mm, pH = 6.0) were added to the reaction using the salan **4c**. The crude product was purified by column chromatography (pentane:Et_2O = 20:1). The reaction product was isolated as a mixture of the two regioisomers **10a** and **10b** as a colourless oil. The ligand used, catalyst loading, and the epoxide yields and ratios are given in **Table S4**.

Table S4: Results of the epoxidation of myrcene (**9**) using the Ti-complexes of the salalen **2** or the salan **4c**.

Entry	Ligand	Catalyst Loading **X**[a] [mol%]	Yield [%]	Ratio **10a**:**10b**[b]	*ee* **10a** [%][c]
1	Salan **4c**	2.0	48	49:1	-
2	Salalen **2**	0.50	79	24:1	30

[a] The catalyst loading corresponds to a dimeric catalyst; [b] determined by ^{1}H-NMR; [c] determined by GC on chiral stationary phase.

10a M[$C_{10}H_{16}O$]: 152.2 g/mol.

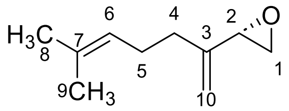

R$_f$ 0.33 (pentane:Et_2O = 20:1).

^{1}H-NMR (500 MHz, $CDCl_3$) δ (ppm) = 5.16 (s, 1H, H-10a), 5.13 – 5.09 (m, 1H, H-6), 4.98 – 4.96 (m, 1H, H-10b), 3.37 – 3.30 (m, 1H, H-2), 2.87 (dd, ^2J = 5.6 Hz, ^3J = 4.1 Hz, 1H, H-1a), 2.65 (dd, ^2J = 5.6 Hz, ^3J = 2.7 Hz, 1H, H-1b), 2.20 – 2.08 (m, 2H, H-5), 2.04 – 1.90 (m, 2H, H-4), 1.69 (s, 3H, H-8/H-9), 1.61 (s, 3H, H-9/H-8).

^{13}C-NMR (126 MHz, $CDCl_3$) δ (ppm) = 145.3 (1C, C-3), 132.0 (1C, C-7), 123.7 (1C, C-6), 112.3 (1C, C-10), 53.8 (1C, C-2), 47.9 (1C, C-1), 30.9 (1C, C-4), 26.7 (1C, C-5), 25.7 (1C, C-8/C-9), 17.7 (1C, C-9/C-8).

GC-MS [STAND40] τ_R = 9.6 min, m/z (%) = 152.3 (1) [M]$^+$, 134.1 (5), 119.2 (30), 109.1 (22), 91.1 (41), 85.1 (20), 79.1 (43), 69.1 (100), 53.1 (39).

FT-IR (ATR): $\tilde{\nu}$ [cm^{-1}] = 2968 (w), 2916 (w), 2857 (w), 1643 (w), 1446 (w), 1377 (w), 1246 (w), 895 (s), 856 (w), 827 (w), 814 (w).

The analytical data are in agreement with the literature.[13]

*Epoxidation of (S)-Citronellene (**11**)*

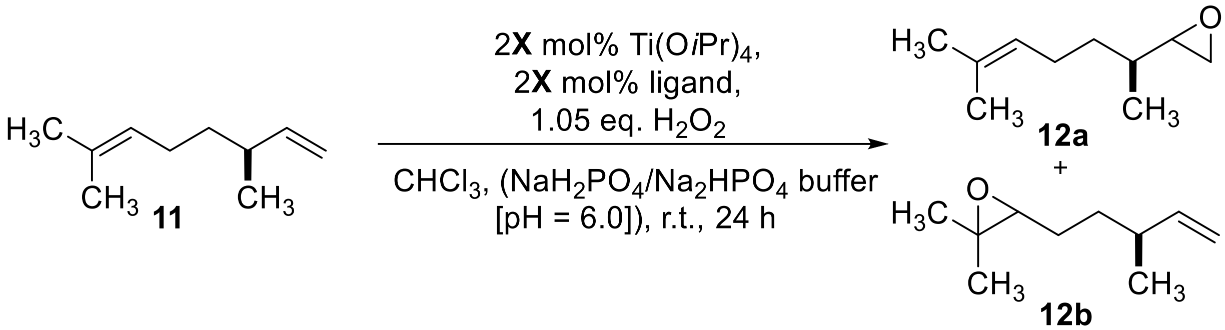

The reactions were performed according to *General Procedure C* on a 1.60 µmol scale. 95 µL (50 w%, 1.05 mmol, 1.05 eq.) H_2O_2 were added. 200 µL of NaH_2PO_4/Na_2HPO_4 buffer (67 mM, pH = 6.0) were added to the reaction using the salan **4c**. The crude product was purified by column chromatography (pentane:Et_2O = 20:1). The reaction product was isolated as a mixture of the regioisomers of **12a** and **12b** as a colourless oil. The ligand used, catalyst loading, and the results are given in **Table S5**.

Table S5: Results of the epoxidation of (*S*)-citronellene (**11**) using the Ti-complexes of the salalen **2** or the salan **4c**.

Entry	Ligand	Catalyst Loading **X** [mol%][a]	Yield [%]	Ratio **12a:12b**[a]	d.r. 12**a** (*syn*:*anti*) [b]
1	Salan **4c**	1.5	51	6:1	3:2
2	Salalen **2**	0.50	54	3:1	1:25
3	Salalen ***ent*-2**	0.50	50	6:1	50:1

[a] The catalyst loading corresponds to a dimeric catalyst; [b] determined by ^{1}H-NMR.

anti-**12a** M[$C_{10}H_{16}O$]: 152.2 g/mol.

R$_f$ 0.36 (pentane:Et_2O = 20:1).

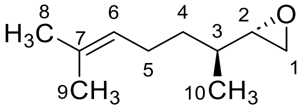

anti-**12a**

^{1}H-NMR (500 MHz, $CDCl_3$) δ (ppm) = 5.14 – 5.08 (m, 1H, H-6), 2.77 – 2.65 (m, 2H, H-1a, H-2), 2.48 (dd, ^2J = 4.9 Hz, ^3J = 2.9 Hz, 1H, H-1b), 2.14 – 1.98 (m, 2H, H-5), 1.68 (s, 3H, H-8/H-9), 1.61 (s, 3H, H-9/H-8), 1.60 – 1.48 (m, 1H, H-4a), 1.38 – 1.31 (m, 2H, H-3, H-4b), 0.93 (d, ^3J = 6.4 Hz, 3H, H-10).

^{13}C-NMR (75 MHz, $CDCl_3$) δ (ppm) = 131.5 (1C, C-7), 124.4 (1C, C-6), 57.0 (1C, C-2), 45.6 (1C, C-1), 35.6 (1C, C-3), 34.6 (1C, C-4), 25.7 (1C, C-8/C-9), 25.4 (1C, C-5), 17.6 (1C, C-9/C-8), 15.5 (1C, C-10).

GC-MS [STAND40] τ_R = 9.5 min, m/z (%) = 154.2 (1) [M]$^+$, 139.1 (6) [M-CH_3]$^+$, 121.1 (13), 105.1 (7), 95.0 (20), 82.1 (92), 67.1 (100), 55.1 (54).

FT-IR (ATR): $\tilde{\nu}$ [cm^{-1}] = 2963 (s), 2916 (s), 2880 (m), 2857 (m), 1456 (m), 1377 (m), 1119 (w), 931 (m), 914 (s), 881 (m), 854 (m), 835 (s).

The analytical data are in agreement with the literature.[14]

syn-**12a** M[$C_{10}H_{16}O$]: 152.2 g/mol.

R$_f$ 0.36 (pentane:Et_2O = 20:1).

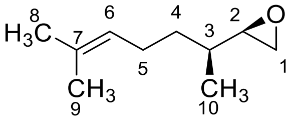

syn-**12a**

**^{1}H-NMR**	(500 MHz, $CDCl_3$) δ (ppm) = 5.10 – 5.04 (m, 1H, H-6), 2.76 (dd, ^2J = 5.0 Hz, ^3J = 4.0 Hz, 1H, H-1a), 2.71 – 2.67 (m, 1H, H-2), 2.53 (dd, ^2J = 5.0 Hz, ^3J = 2.8 Hz, 1H, H-1b), 2.09 – 1.95 (m, 2H, H-5), 1.68 (s, 3H, H-8/H-9), 1.60 (s, 3H, H-9/H-8), 1.49 – 1.24 (m, 3H, H-4, H-3), 1.03 (d, ^3J = 6.3 Hz, 3H, H-10).
**^{13}C-NMR**	(75 MHz, $CDCl_3$) δ (ppm) = 131.7 (1C, C-7), 124.2 (1C, C-6), 57.0 (1C, C-2), 46.9 (1C, C-1), 35.7 (1C, C-3), 33.6 (1C, C-4), 25.7 (1C, C-8/C-9), 25.5 (1C, C-5), 17.7 (1C, C-9/C-8), 17.0 (1C, C-10).
**GC-MS**	[STAND40] $τ_R$ = 9.5 min, m/z (%) = 154.1 (1) $[M]^+$, 139.2 (8) $[M-CH_3]$, 121.1 (12), 109.2 (10), 95.1 (25), 82.1 (100), 67.1 (100), 55.1 (57).
**FT-IR**	(ATR): $\tilde{\nu}$ [cm^{-1}] = 2965 (s), 2916 (s), 2857 (m), 1454 (m), 1377 (m), 1256 (w), 1117 (w), 914 (s), 893 (s), 856 (s), 824 (s).

The analytical data are in agreement with the literature.[14]

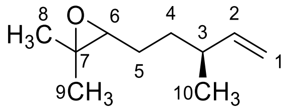

syn+anti-**12b**

**12b**	M[$C_{10}H_{16}O$]: 152.2 g/mol.
**R_f**	0.36 (pentane:Et_2O = 20:1).
**^{1}H-NMR**	(500 MHz, $CDCl_3$) δ (ppm) = 5.74 – 5.61 (m, 1H, H-2), 5.02 – 4.91 (m, 2H, H-1), 2.76 – 2.70 (m, 1H, H-6), 2.21 – 2.16 (m, 1H, H-3), 1.60 – 1.54 (m, 3H, H-4a, H-5), 1.37 – 1.32 (m, 1H, H-4b), 1.31 (s, 3H, H-8/H-9), 1.26 (s, 3H, H-9/H-8), 1.02 (d, ^3J = 6.7 Hz, 3H, H-10).
**^{13}C-NMR**	(126 MHz, $CDCl_3$) δ (ppm) = 144.2 (0.5C, C-2), 144.1 (0.5C, C-2), 113.1 (0.5C, C-1), 112.9 (0.5C, C-1), 64.5 (0.5C, C-6), 64.4 (0.5C, C-6), 58.3 (0.5C, C-7), 58.2 (0.5C, C-7), 37.8 (0.5C, C-3), 37.6 (0.5C, C-3), 33.3 (0.5C, C-4), 33.1 (0.5C, C-4), 26.8 (0.5C, C-5), 26.6 (0.5C, C-5), 24.9 (1C, C-8/C-9), 20.3 (0.5C, C-10), 20.1 (0.5C, C-10), 18.7 (0.5C, C-9/C-8), 18.7 (0.5C, C-9/C-8).
**GC-MS**	[STAND40] $τ_R$ = 8.5 min, m/z (%) = 136.0 (3) $[M-H_2O]$, 106.9 (7), 95.0 (17), 81.0 (61), 67.0 (39), 55.1 (100).

The NMR-data were extracted from the 2D-NMR experiments of the mixture of *anti*-**12a** and *syn*- and *anti*-**12b**. Two diastereomeres were observed. Because of this, two sets of signals are given in the ^{13}C-NMR. The analytical data are in agreement with the literature.[15]

*Epoxidation of (R)-Linalool (**13**)*

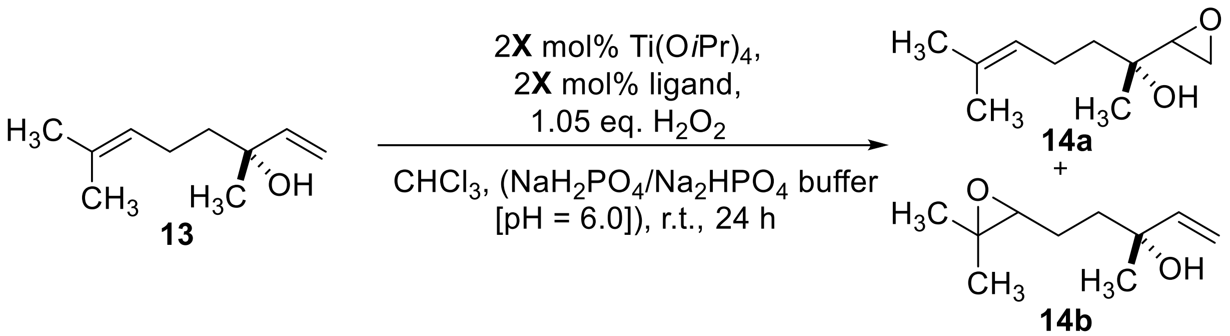

The reactions were performed according to *General Procedure C* on a 1.60 µmol scale. 109 µL (50 w%, 1.20 mmol, 1.2 eq.) H_2O_2 were added. 200 µL of NaH_2PO_4/Na_2HPO_4 buffer (67 mM, pH = 6.0) were added to the reaction using the salan **4c**. The crude product was purified by column chromatography (pentane:Et_2O = 4:1). The reaction product was isolated as a mixture of the regioisomers of **14a** and **14b** as a colourless liquid. The ligand used, catalyst loading, and the results are given in **Table S6**.

Table S6: Results of the epoxidation of (*R*)-linalool (**13**) using the Ti-complexes of the salalen **2** or the salan **4c**.

Entry	Ligand	Catalyst Loading **X** [mol%][a]	Yield [%][b]	Ratio **14a**:**14b**[c]	d.r. **14a** (*syn*:*anti*)[c]
1	Salan **4c**	2	23	3:1	9:1
2	Salalen **2**	1.5	42	19:1	24:1
3	Salalen *ent*-**2**	1.5	22	3:1	1:4

[a] The catalyst loading corresponds to a dimeric catalyst; [b] the yields are given after column chromatography; however, the products 14a and 14b appear to be not stable on silica. Higher yields of approx.. 80% could be obtained by bulb-to-bulb distillation. However, a separation from the starting material and diepoxide was not possible; [c]determined by ^{1}H-NMR.

syn-**14a** M[$C_{10}H_{18}O_2$]: 170.2 g/mol.

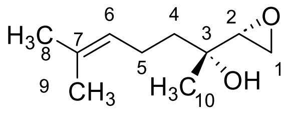

syn-**14a**

R$_f$ 0.17 (pentane:Et_2O = 9:1).

^{1}H-NMR (500 MHz, $CDCl_3$) δ (ppm) = 5.15 – 5.10 (m, 1H, H-6), 2.97 (dd, ^3J = 4.1 Hz, ^3J = 2.9 Hz, 1H, H-2), 2.77 (dd, ^2J = 5.1 Hz , ^3J = 2.9 Hz, 1H, H-1a), 2.70 (dd, ^2J = 5.1, ^3J = 4.1 Hz, 1H, H-1b), 2.16 – 2.08 (m, 2H, H-5), 1.84 (s, 1H, O*H*), 1.69 (s, 3H, H-8/H-9), 1.65 – 1.59 (m, 6H, H-4, H-9/H-8), 1.19 (s, 3H, H-10).

^{13}C-NMR (75 MHz, $CDCl_3$) δ (ppm) = 132.0 (1C, C-7), 124.0 (1C, C-6), 69.3 (1C, C-3), 57.6 (1C, C-2), 43.3 (1C, C-1), 41.3 (1C, C-4), 25.7 (1C, C-8/C-9), 22.8 (1C, C-10), 22.2 (1C, C-5), 17.7 (1C, C-9/C-8).

GC-MS [STAND40] τ_R = 10.3 min, m/z (%) = 152.1 (8) [M-H_2O], 137.1 (6), 119.2 (12), 109.2 (41), 91.0 (31), 82.1 (56), 69.2 (98), 67.2 (100), 55.1 (72).

FT-IR (ATR): $\tilde{\nu}$ [cm^{-1}] = 3449 (b), 2972 (m), 2926 (m), 2858 (w), 1450 (m), 1377 (m), 1341 (w), 1260 (w), 1163 (w), 1115 (m), 1092 (m), 1072 (m), 1017 (w), 1001 (w), 943 (w), 914 (s), 872 (s), 837 (m), 810 (m), 721 (w).

The analytical data are in agreement with the literature.[16]

anti-**14a** M[$C_{10}H_{18}O_2$]: 170.2 g/mol.

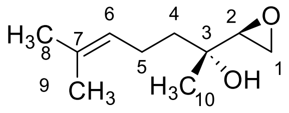

anti-**14a**

R$_f$ 0.17 (pentane:Et_2O = 9:1).

^{1}H-NMR (500 MHz, $CDCl_3$) δ (ppm) = 5.14 – 5.08 (m, 1H, H-6), 2.92 (dd, ^3J = 4.0 Hz, ^3J = 2.8 Hz, 1H, H-2), 2.85 (dd, ^2J = 5.1 Hz, ^3J = 2.8 Hz, 1H, H-1a), 2.74 (dd, ^2J = 5.1 Hz, ^3J = 4.0 Hz, 1H, H-1b), 2.17 – 2.04 (m, 3H, H-5), 1.69 (s, 4H, H-8/H-9, O*H*), 1.62 (s, 3H, H-9/H-8), 1.60 – 1.53 (m, 2H, H-4), 1.31 (s, 3H, H-10).

^{13}C-NMR (75 MHz, $CDCl_3$) δ (ppm) = 144.3 (1C, C-7), 124.1 (1C, C-6), 69.2 (1C, C-3), 57.8 (1C, C-2), 44.3 (1C, C-1), 38.6 (1C, C-4), 26.2 (1C, C-10), 25.7 (1C, C-8/C-9), 22.0 (1C, C-5), 17.7 (1C C-9/C-8).

FT-IR (ATR): $\tilde{\nu}$ [cm^{-1}] = 3460 (b), 2970 (m), 2926 (w), 3860 (w), 1454 (w), 1375 (m), 1328 (w), 1260 (w), 1132 (m), 1094 (w), 1030 (w), 988 (w), 918 (s), 870 (s), 837 (w), 814 (w), 625 (w).

The analytical data are in agreement with the literature.[17] The compound decomposes on the GC-MS.

2 NMR-Spectra

*5-Bromo-1-pentene Oxide (rac-**8a**)*

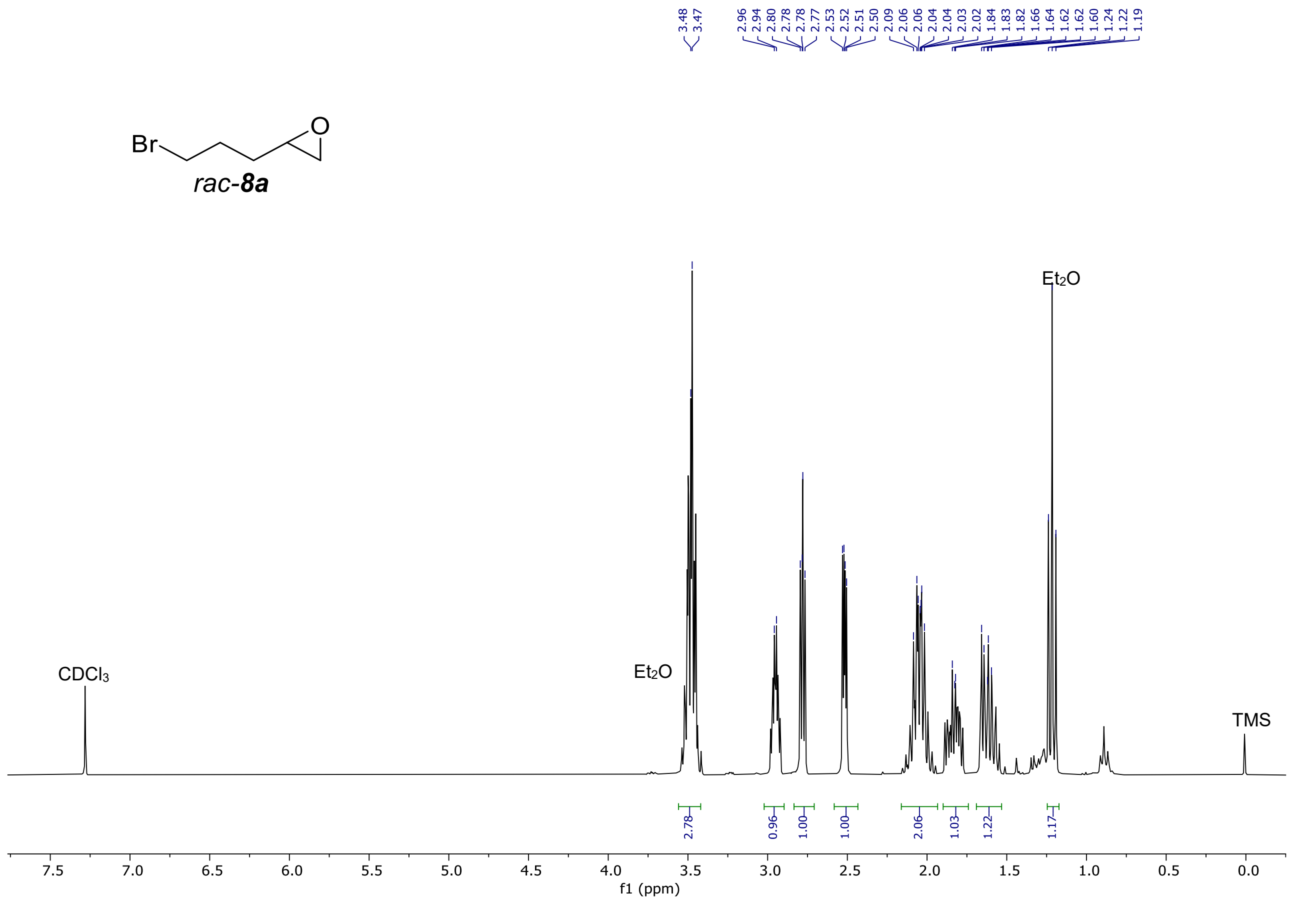

Figure S10: ^{1}H-NMR of 5-bromo-1-pentene oxide (*rac*-**8a**) isolated from the epoxidation reaction (300 MHz, $CDCl_3$, see experiment on page 20).

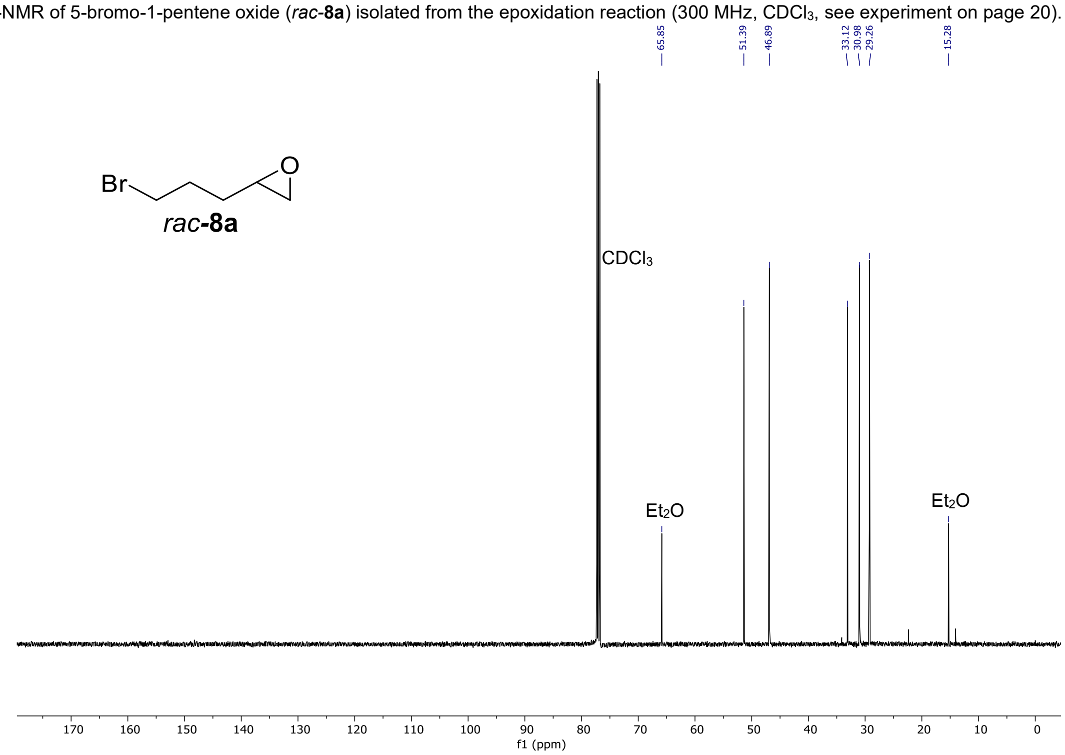

Figure S11: ^{13}C-NMR of 5-bromo-1-pentene oxide (*rac*-**8a**) isolated from the epoxidation reaction (125 MHz, $CDCl_3$, see experiment on page 20).

*1-Decene Oxide (rac-**8b**)*

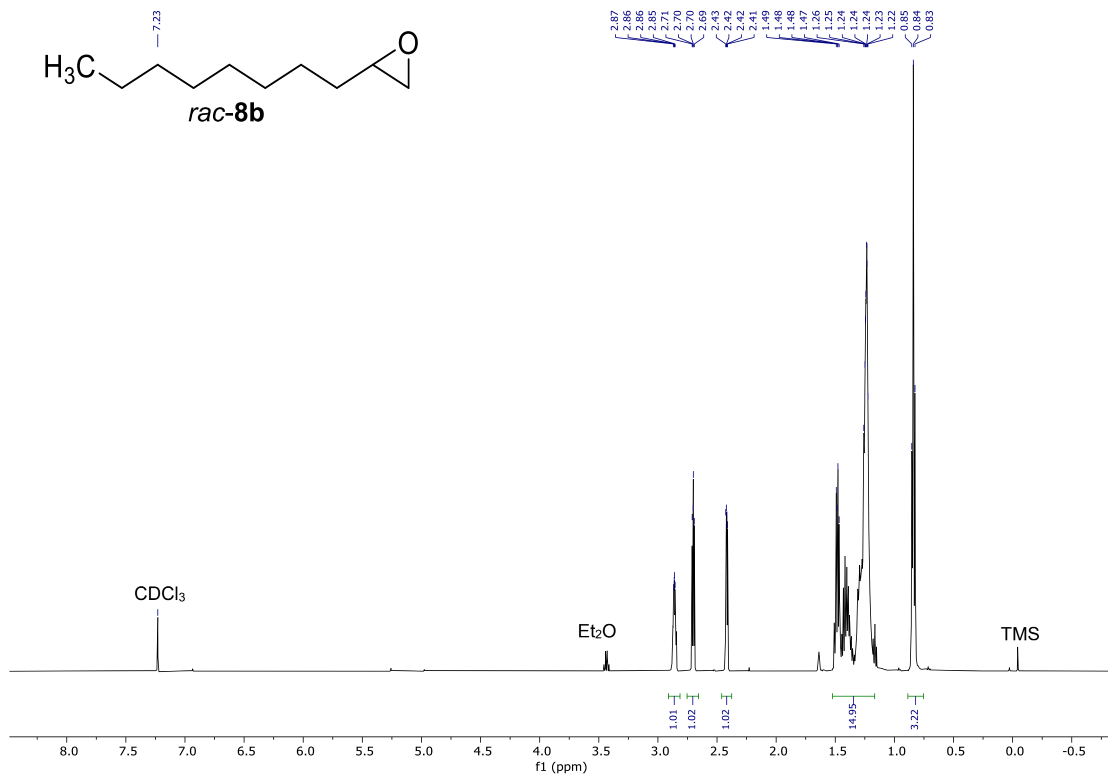

Figure S12: ^{1}H-NMR of 1-decene oxide (*rac*-**8b**) isolated from the epoxidation reaction (500 MHz, $CDCl_3$, see experiment on page 21).

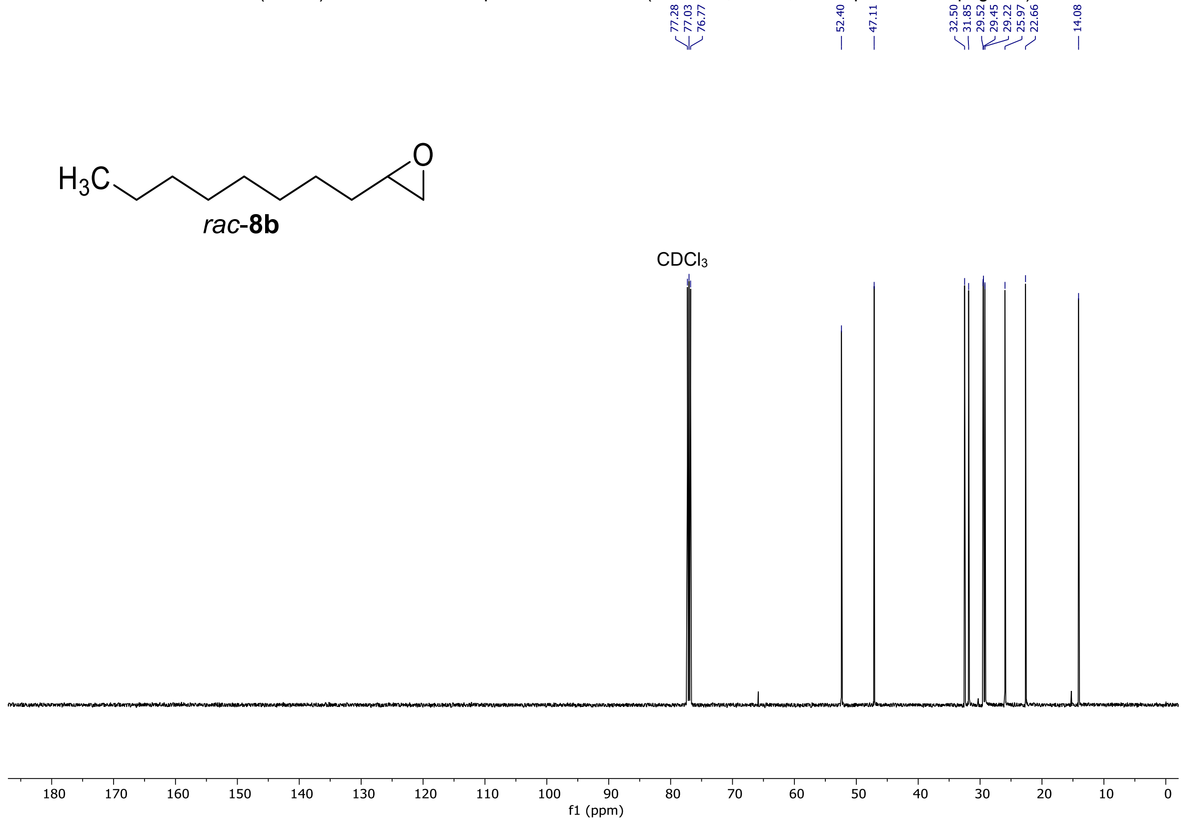

Figure S13: ^{13}C-NMR of 1-decene oxide (*rac*-**8b**) isolated from the epoxidation reaction (125 MHz, $CDCl_3$, see experiment on page 21).

*2-Cyclohexyloxirane (rac-**8c**)*

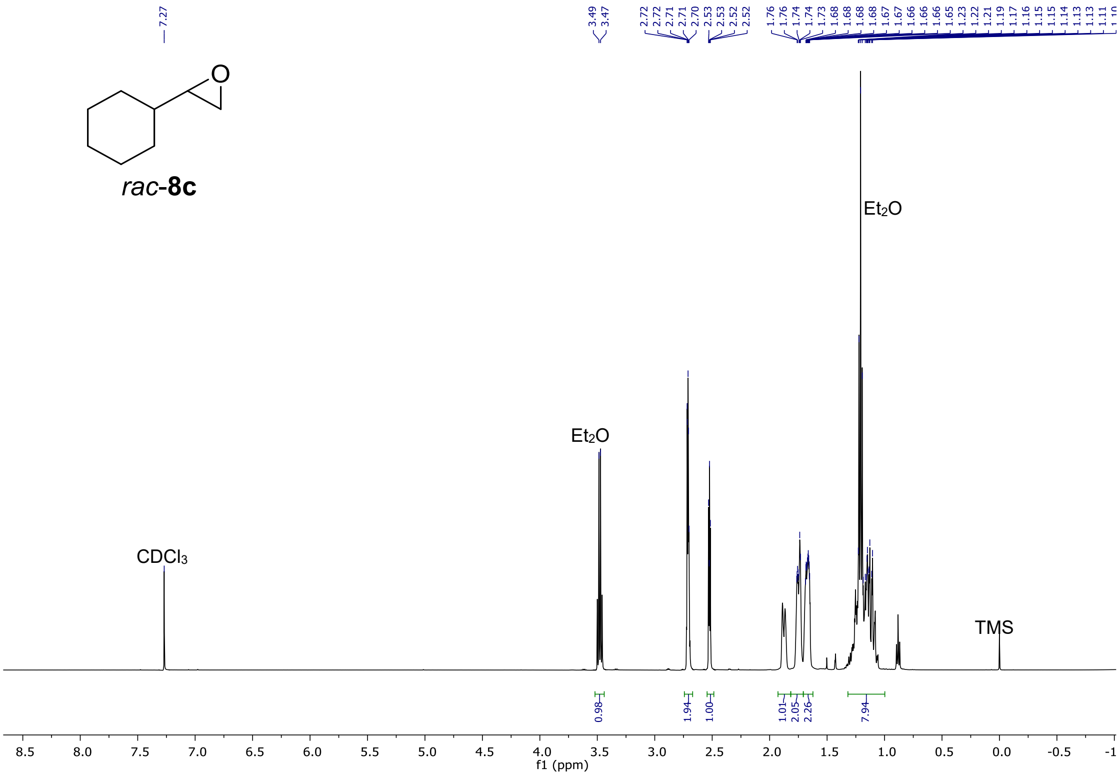

Figure S14: ^{1}H-NMR of 2-cyclohexyloxirane (*rac*-**8c**) isolated from the epoxidation reaction (500 MHz, $CDCl_3$, see experiment on page 22).

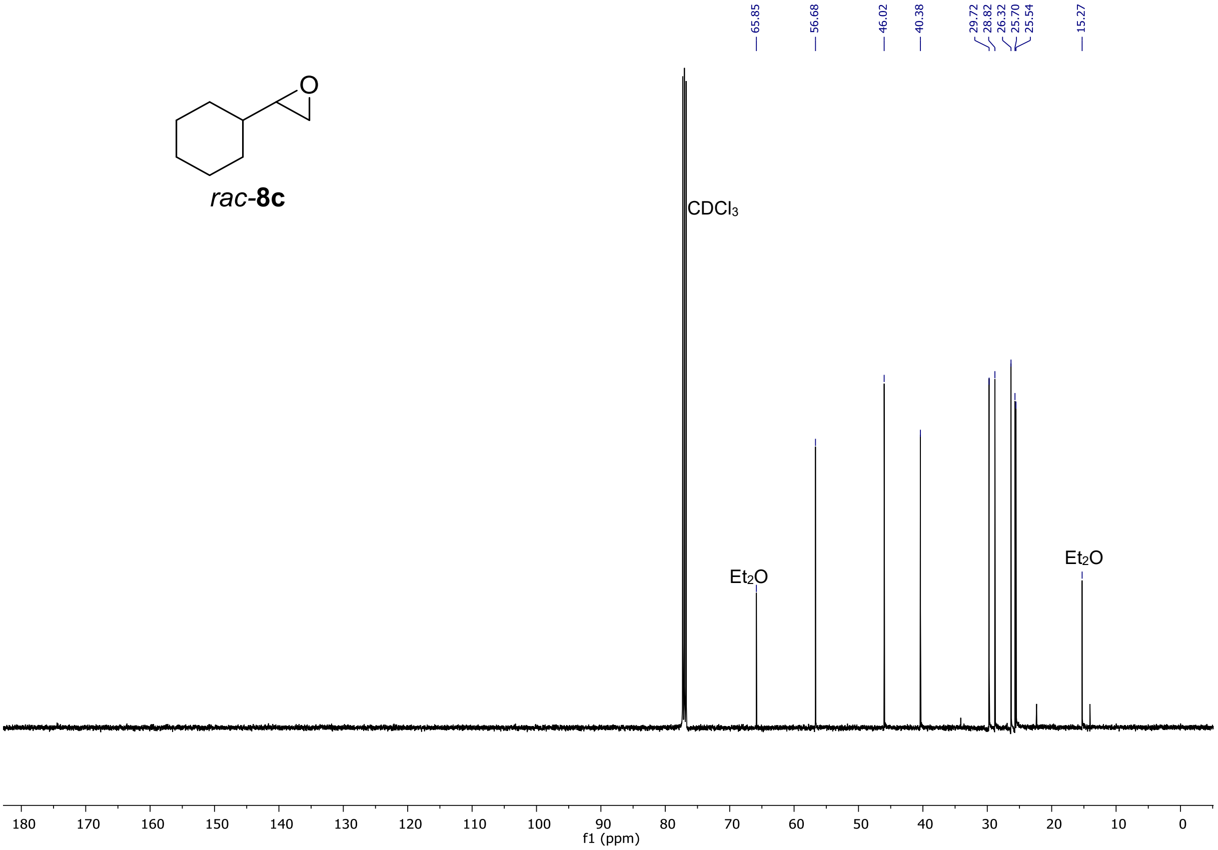

Figure S15: ^{13}C-NMR of 2-cyclohexyloxirane (*rac*-**8c**) isolated from the epoxidation reaction (75 MHz, $CDCl_3$, see experiment on page 22).

*Styrene Oxide (rac-**8d**)*

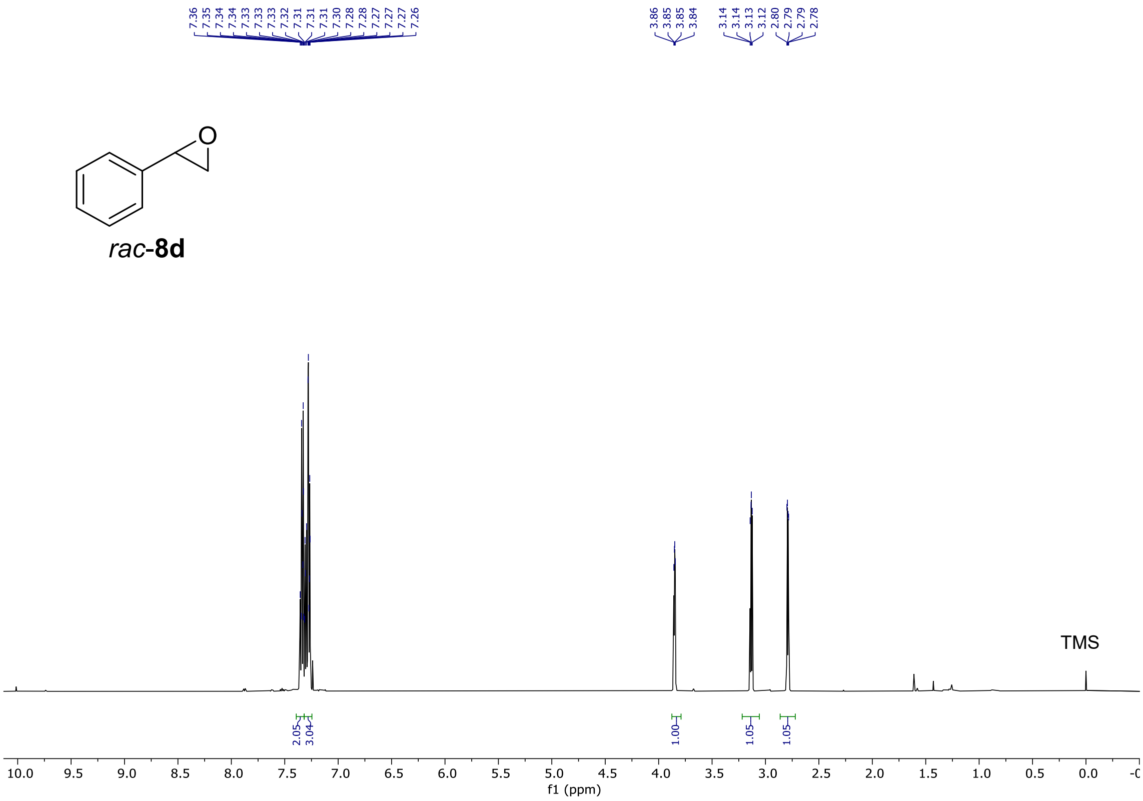

Figure S16: ^{1}H-NMR of styrene oxide (*rac*-**8d**) isolated from the epoxidation reaction (500 MHz, $CDCl_3$, see experiment on page 22).

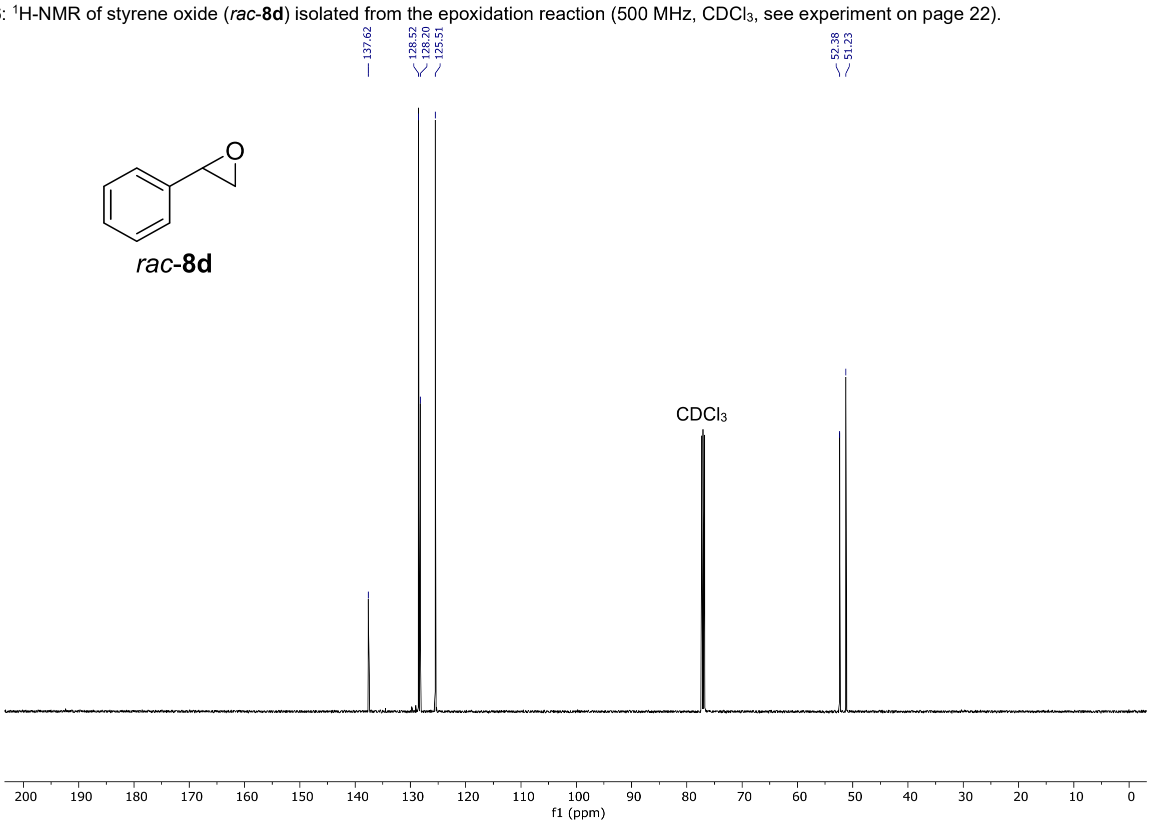

Figure S17: ^{13}C-NMR of styrene oxide (*rac*-**8d**) isolated from the epoxidation reaction (125 MHz, $CDCl_3$, see experiment on page 22).

*Cis-2-Octene Oxide (rac-**8e**)*

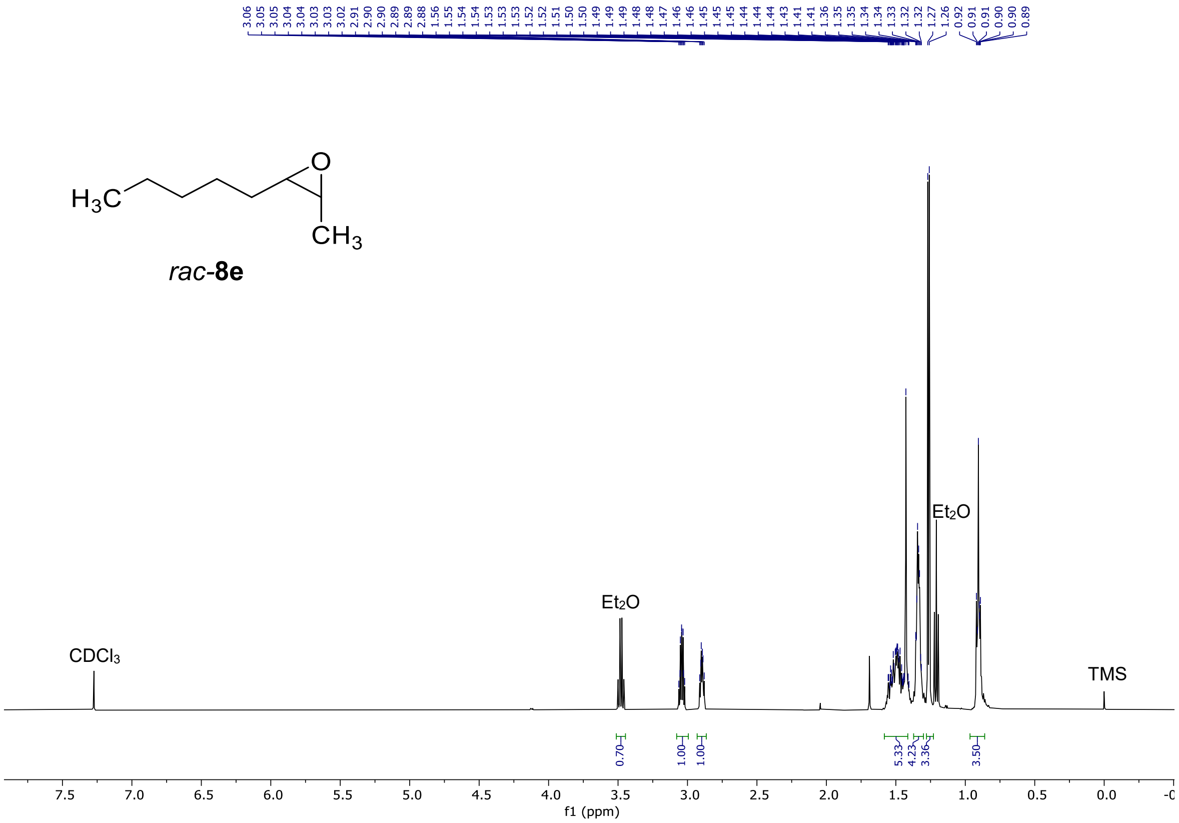

Figure S18: ^{1}H-NMR of *cis*-2-octene oxide (*rac*-**8e**) isolated from the epoxidation reaction (500 MHz, $CDCl_3$, see experiment on page 23).

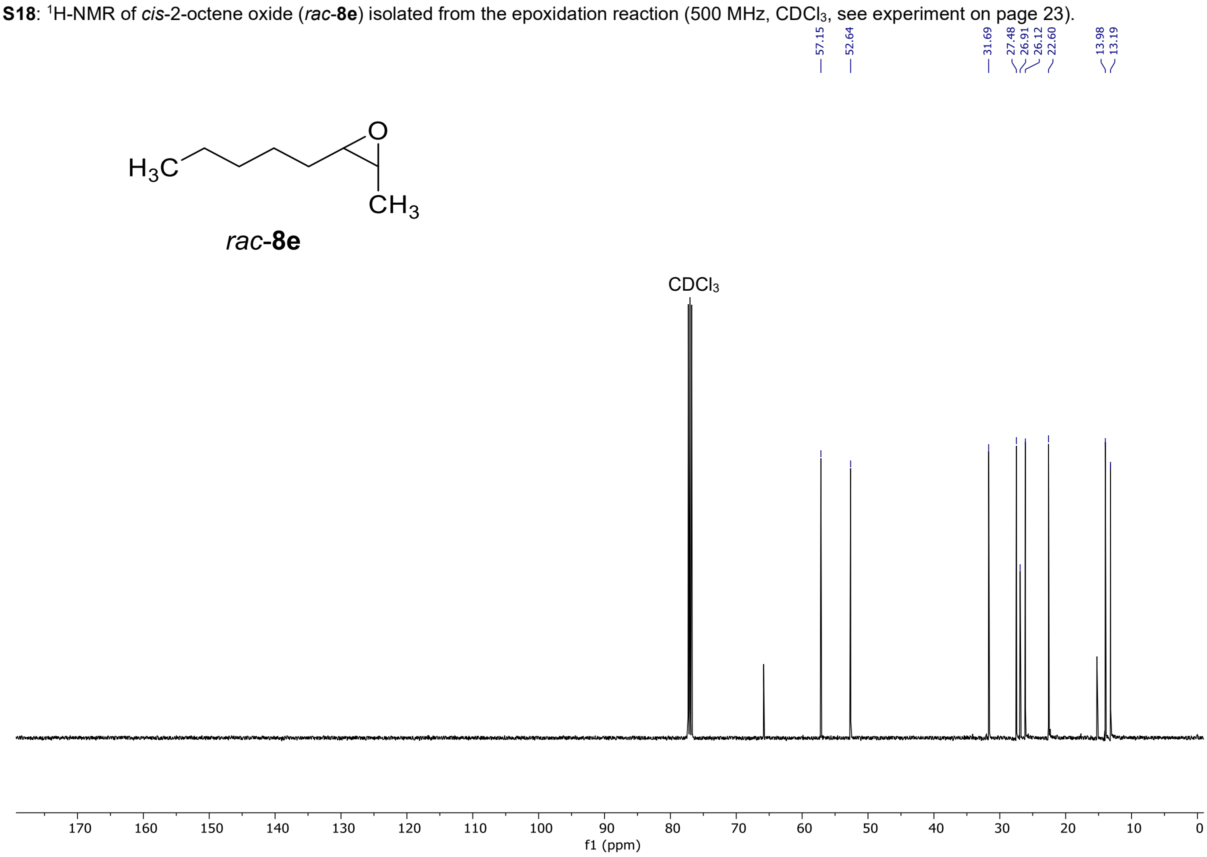

Figure S19: ^{13}C-NMR of *cis*-2-octene oxide (*rac*-**8e**) isolated from the epoxidation reaction (125 MHz, $CDCl_3$, see experiment on page 23).

*1,2-Dihydronaphthalene Oxide (rac-**8f**)*

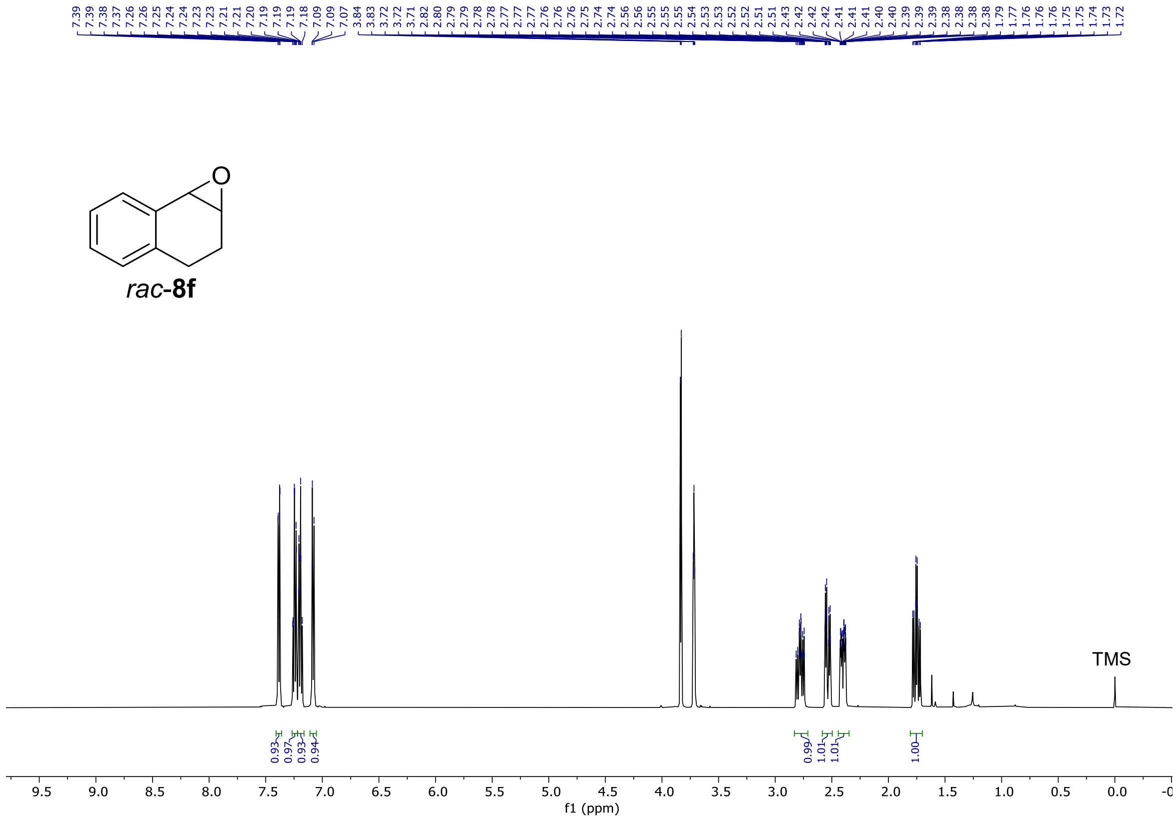

Figure S20: ^{1}H-NMR of 1,2-dihydronaphthalene oxide (*rac*-**8f**) isolated from the epoxidation reaction (500 MHz, $CDCl_3$, see experiment on page 24).

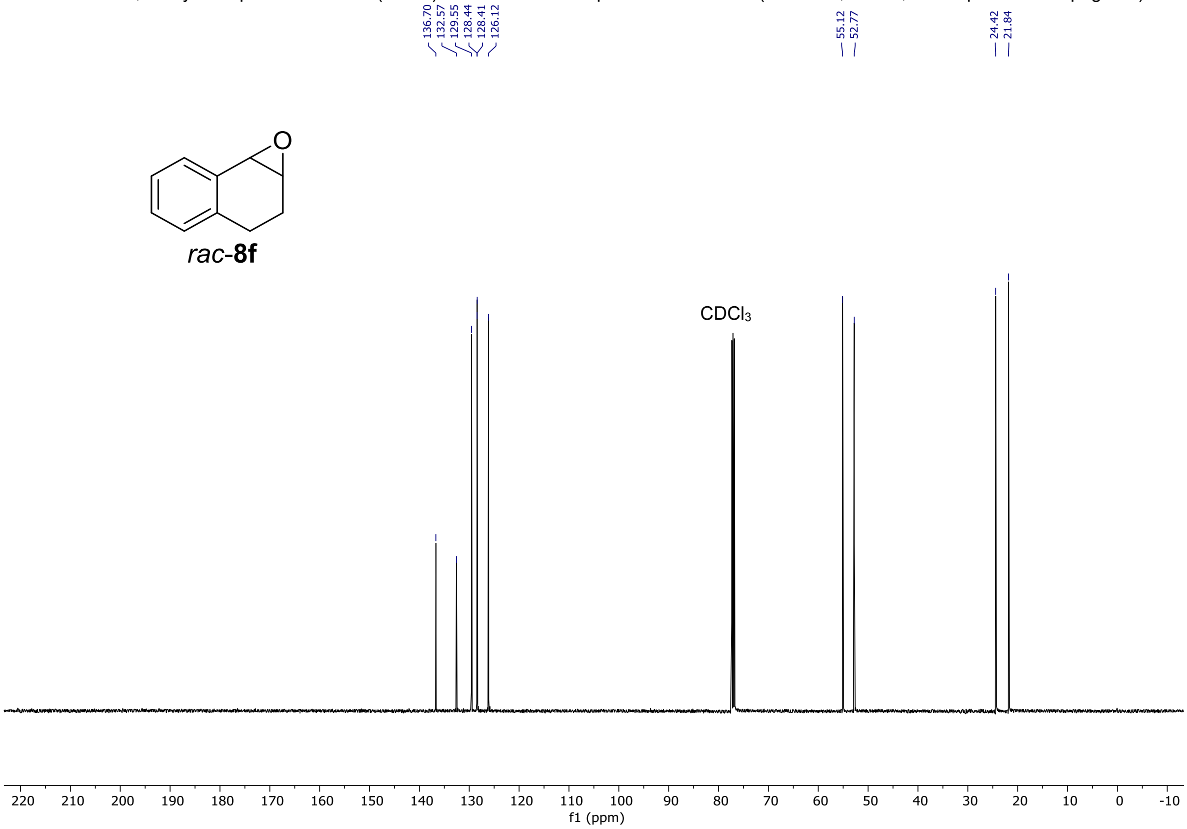

Figure S21: ^{13}C-NMR of 1,2-dihydronaphthalene oxide (*rac*-**8f**) isolated from the epoxidation reaction (125 MHz, $CDCl_3$, see experiment on page 24).

*Trans-2-Octene Oxide (rac-**8g**)*

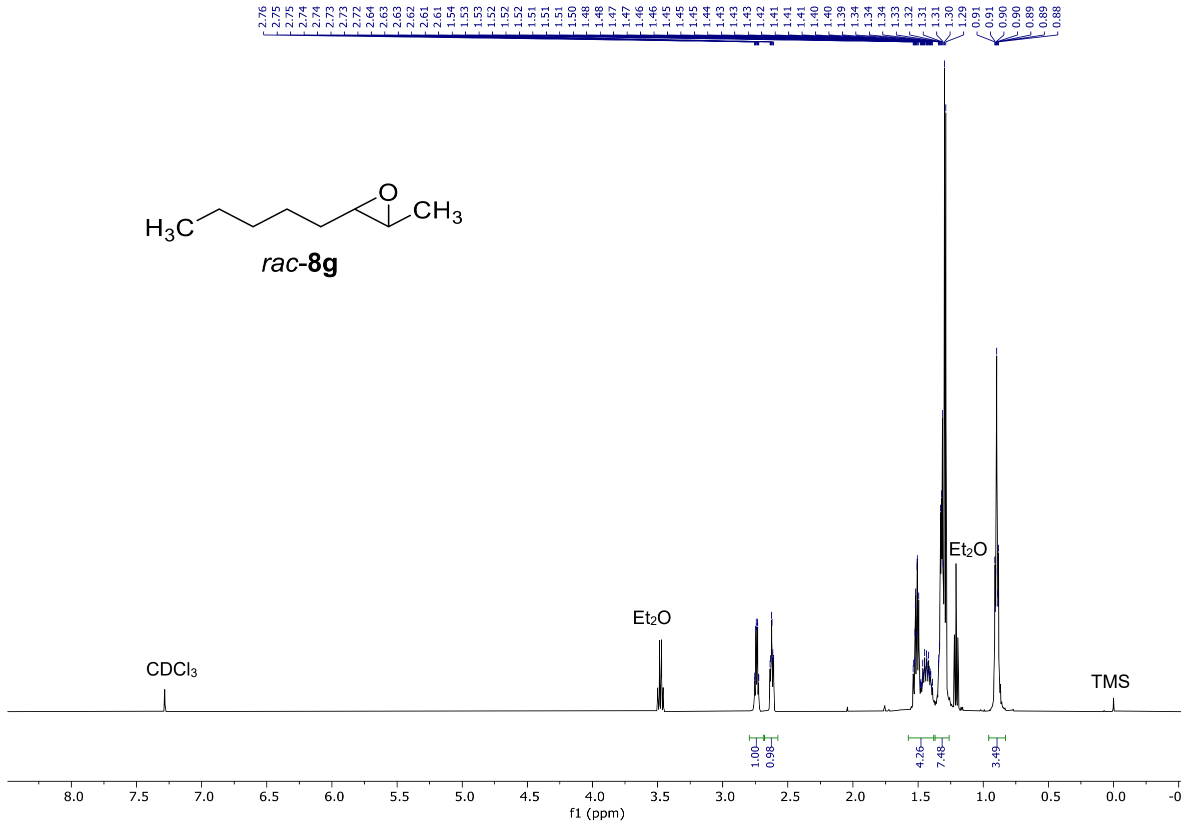

Figure S22: ^{1}H-NMR of *trans*-2-octene oxide (*rac*-**8g**) isolated from the epoxidation reaction (500 MHz, $CDCl_3$, see experiment on page 24).

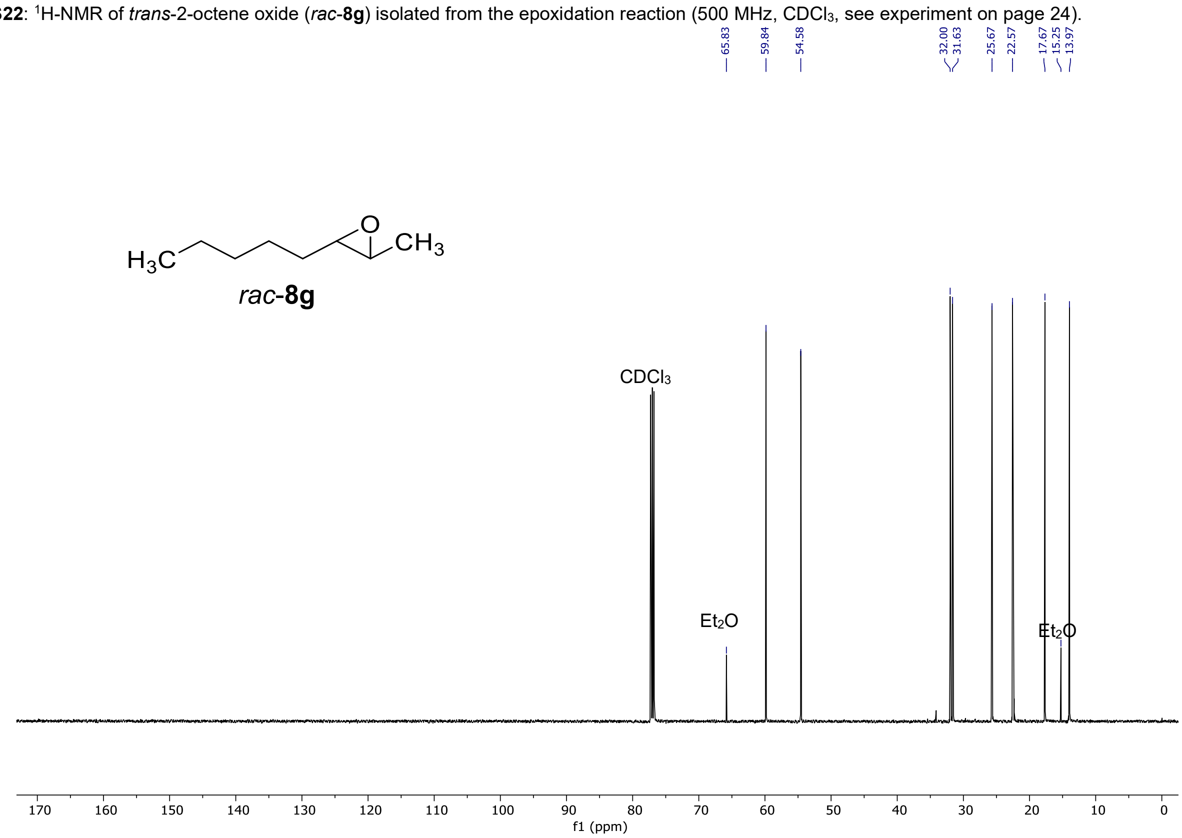

Figure S23: ^{13}C-NMR *trans*-2-octene oxide (*rac*-**8g**) isolated from the epoxidation reaction (125 MHz, $CDCl_3$, see experiment on page 24).

*2-Methyl-2-cyclohexyloxirane (rac-**8h**)*

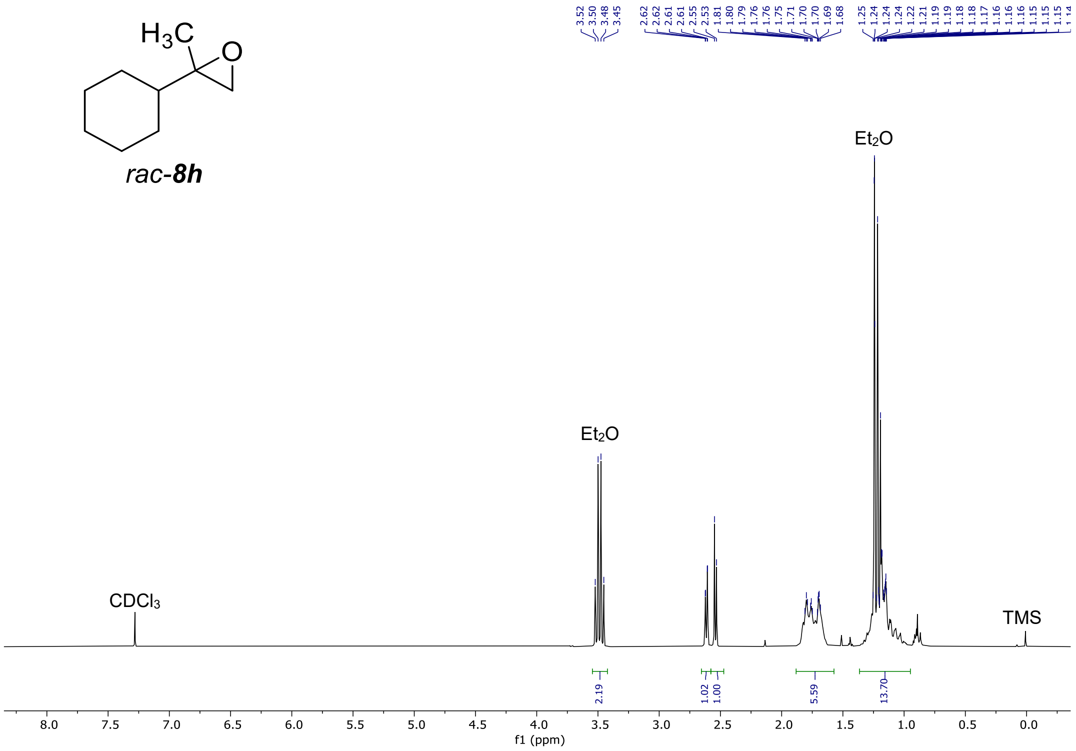

Figure S24: ^{1}H-NMR of 2-methyl-2-cyclohexyloxirane (*rac*-**8h**) isolated from the epoxidation reaction (500 MHz, $CDCl_3$, see experiment on page 25).

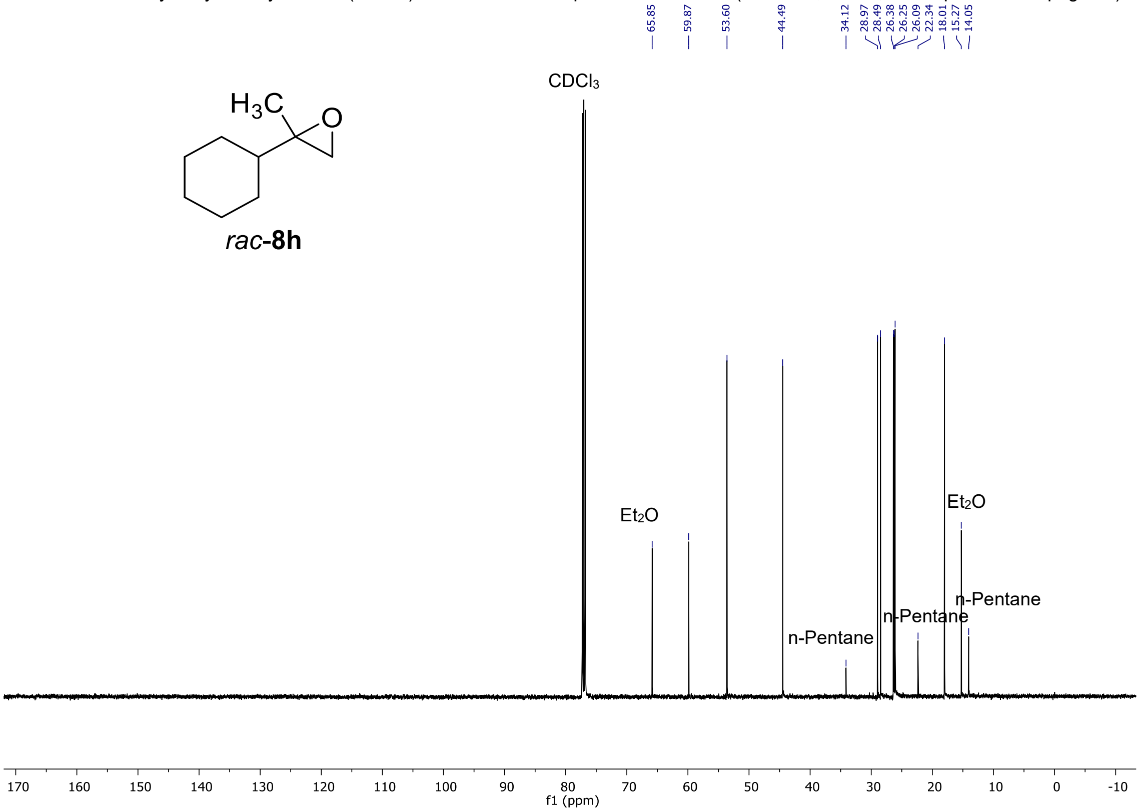

Figure S25: ^{13}C-NMR of 2-methyl-2-cyclohexyloxirane oxide (*rac*-**8h**) isolated from the epoxidation reaction (75 MHz, $CDCl_3$, see experiment on page 25).

*2-Methyl-2-Octene Oxide (rac-**8i**)*

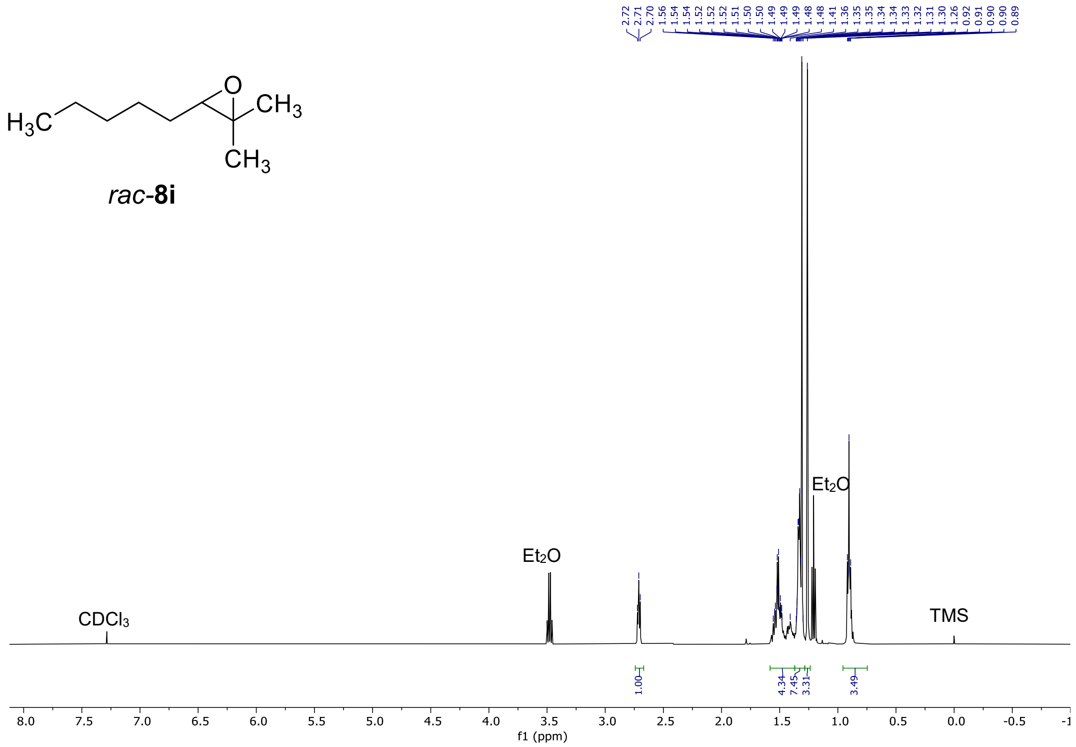

Figure S26: ^{1}H-NMR of 2-methyl-2-octene oxide (*rac*-**8i**) isolated from the epoxidation reaction (500 MHz, $CDCl_3$, see experiment on page 26).

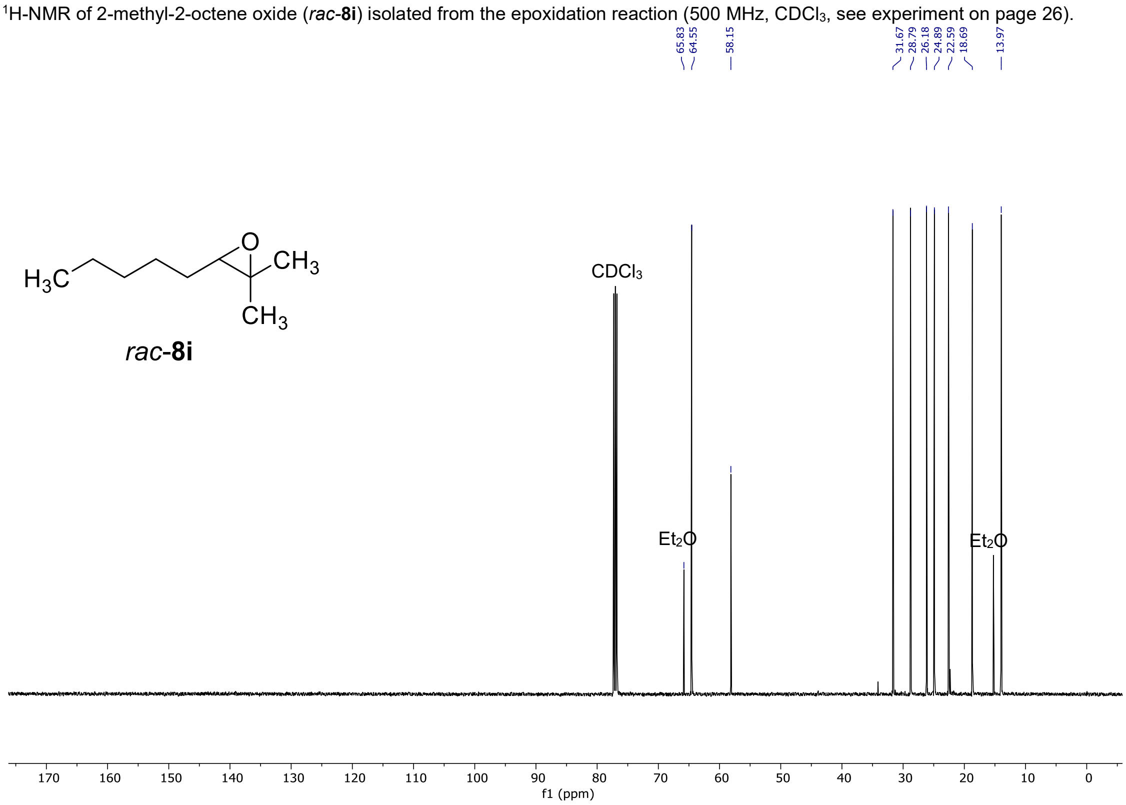

Figure S27: ^{13}C-NMR of 2-methyl-2-octene oxide (*rac*-**8i**) isolated from the epoxidation reaction (125 MHz, $CDCl_3$, see experiment on page 26).

*1-Phenylcyclohexene Oxide (rac-**8j**)*

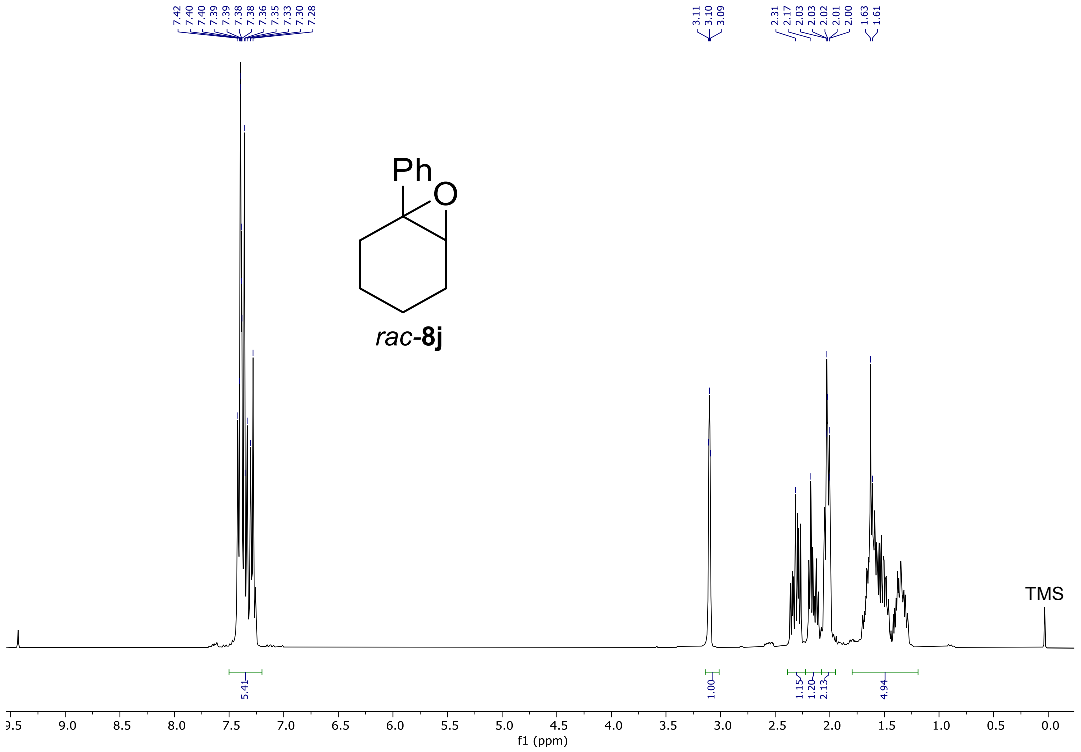

Figure S28: ^{1}H-NMR of 1-phenylcyclohexene oxide (*rac*-**8j**) isolated from the epoxidation reaction (500 MHz, $CDCl_3$, see experiment on page 26).

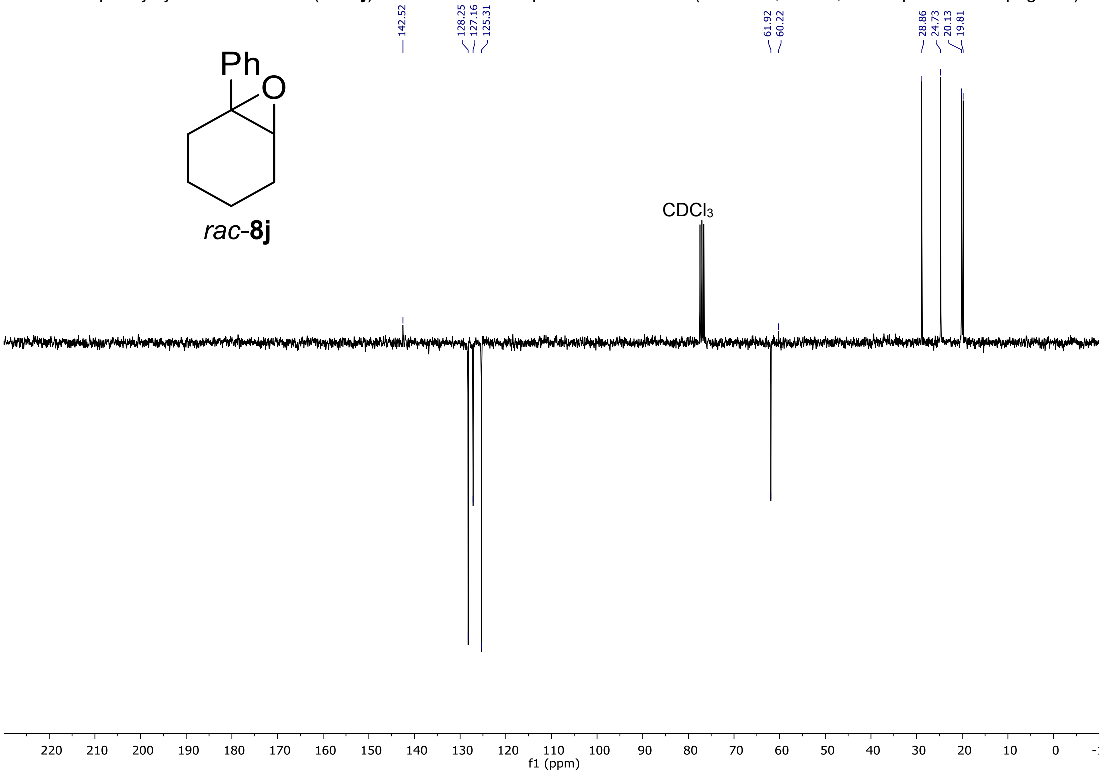

Figure S29: ^{13}C-NMR of 1-phenylcyclohexene oxide (*rac*-**8j**) isolated from the epoxidation reaction (125 MHz, $CDCl_3$, see experiment on page 26).

*1-(Oxiran-2-yl)-phenyl methanol (rac-**8k**, syn/anti-mixture)*

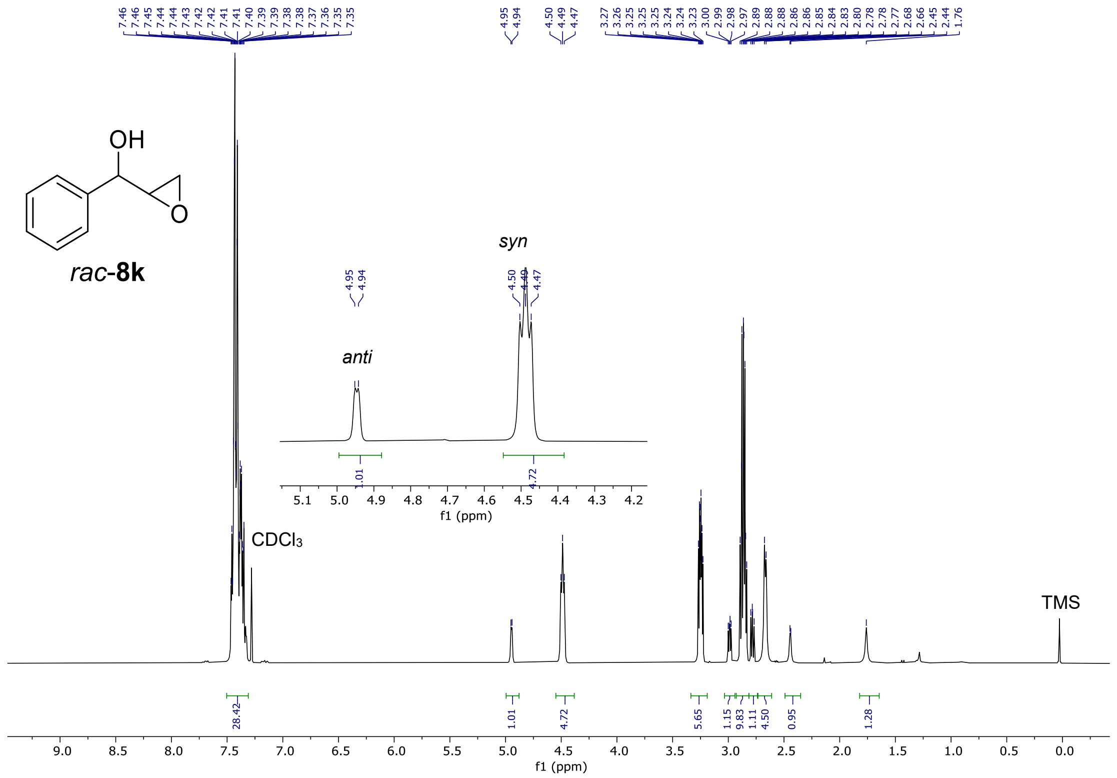

Figure S30: ^{1}H-NMR of 1-(oxiran-2-yl)-phenyl methanol (*rac*-**8k**, *syn/anti*-mixture) isolated from the epoxidation reaction (500 MHz, $CDCl_3$, see experiment on page 27).

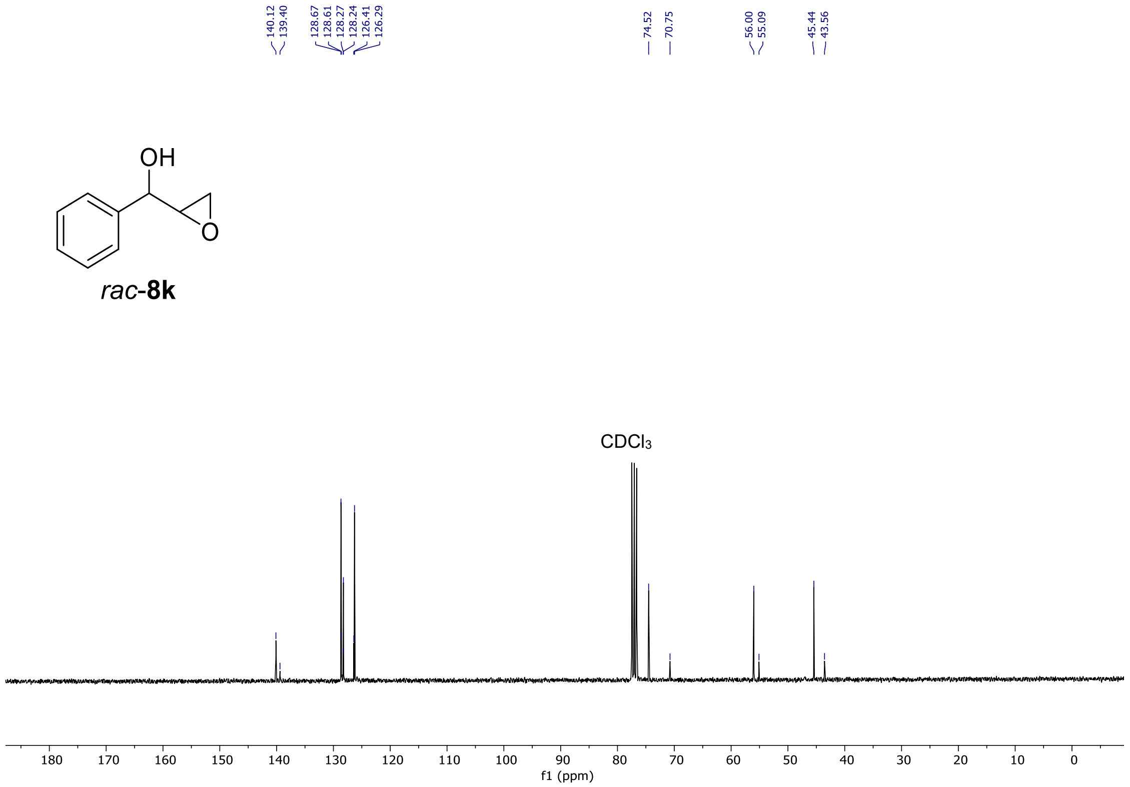

Figure S31: ^{13}C-NMR of 1-(oxiran-2-yl)-phenyl methanol (*rac*-**8k**, *syn/anti*-mixture) isolated from the epoxidation reaction (125 MHz, $CDCl_3$, see experiment on page 27).

*Myrcene Oxides rac-**10a** and rac-**10b***

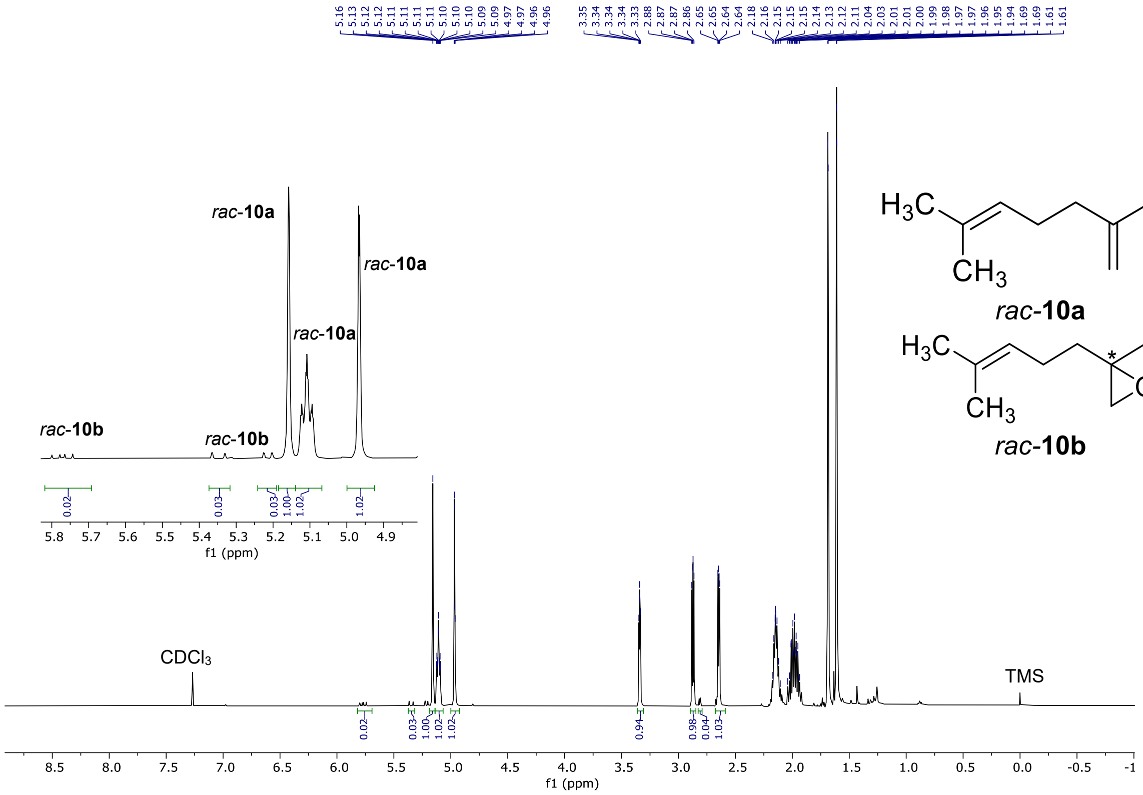

Figure S32: ^{1}H-NMR of myrcene oxides *rac*-**10a** and *rac*-**10b** in a 49:1 mixture isolated from the epoxidation reaction using salan **4c** (500 MHz, $CDCl_3$, see experiment on page 28).

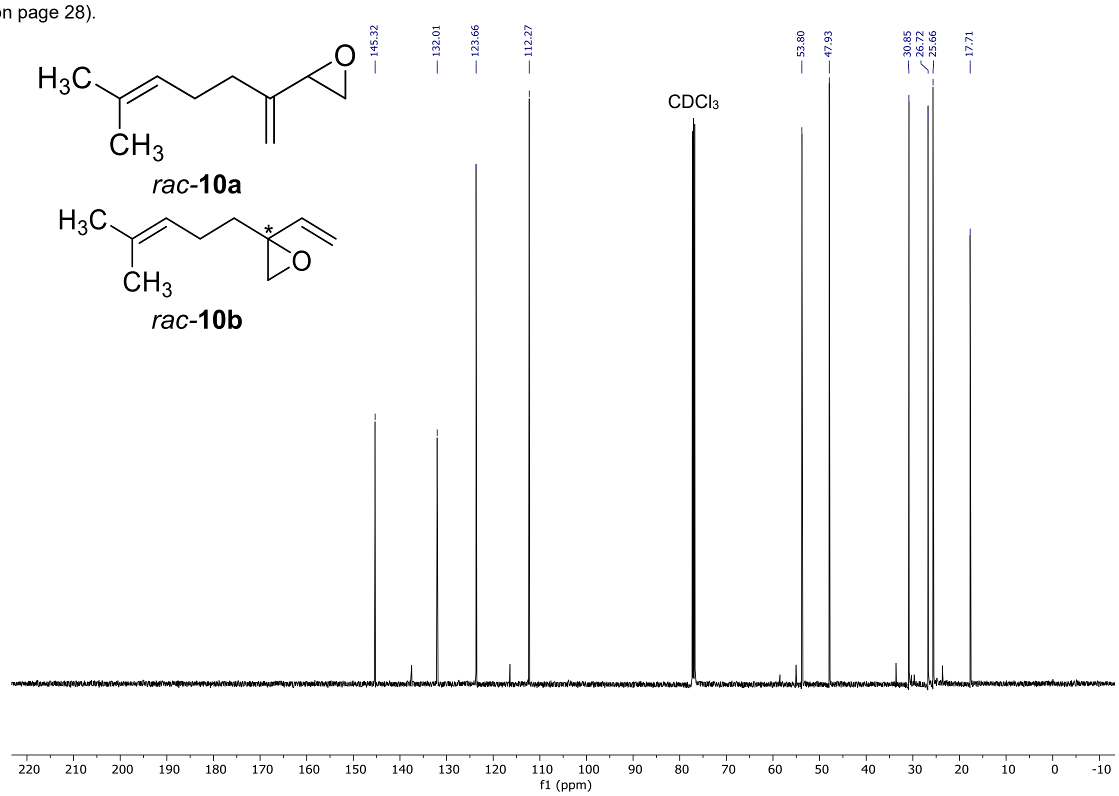

Figure S33: ^{13}C-NMR of myrcene oxides *rac*-**10a** and *rac*-**10b** in a 49.1 mixture isolated from the epoxidation reaction using salan **4c** (125 MHz, $CDCl_3$, see experiment on page 28).

*(S)-Citronellene Oxides **12a** and **12b***

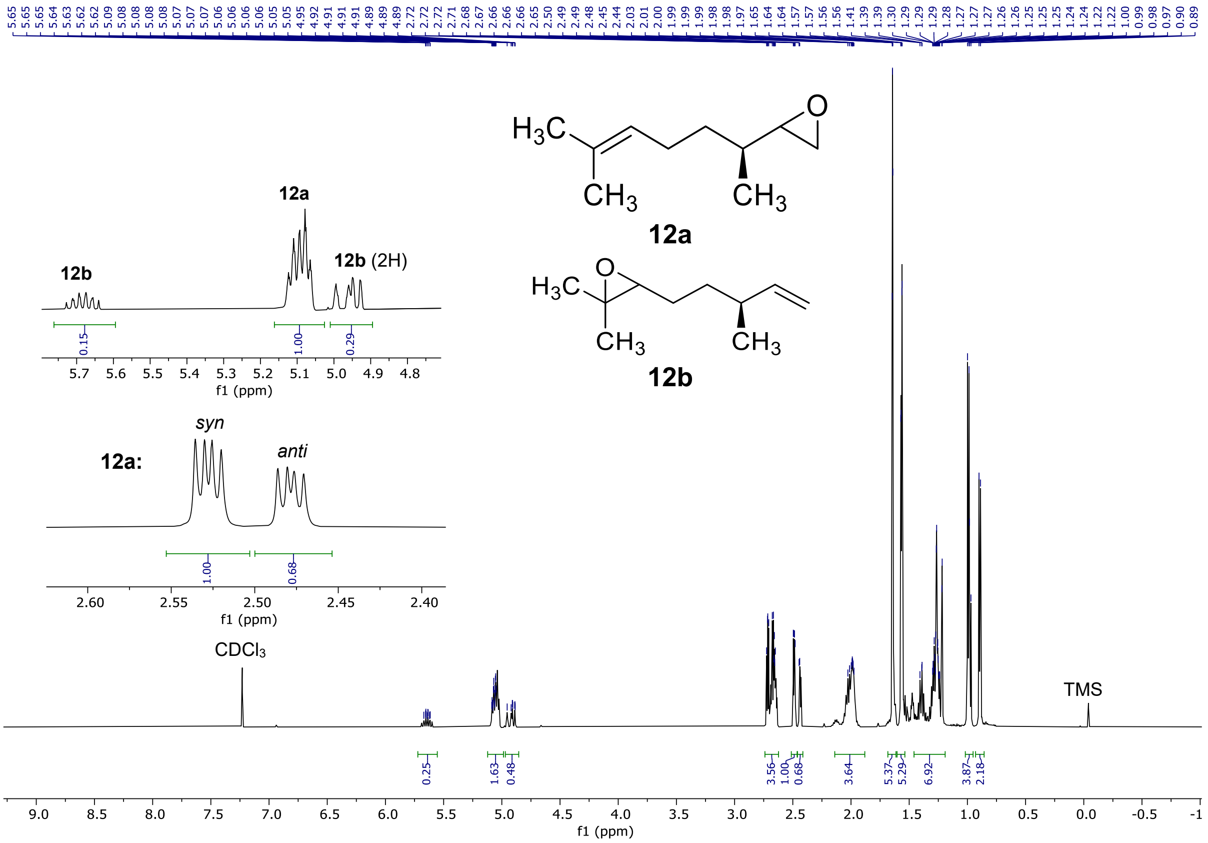

Figure S34: ^{1}H-NMR of (*S*)-citronellene oxides **12a** and **12b** in a 6:1 mixture (3:2 *syn:anti* for **12a**) isolated from the epoxidation reaction using salan **4c** (500 MHz, $CDCl_3$, see experiment on page 29). The integration in the excerpts was done separately from the whole spectrum to show the ratios more clearly.

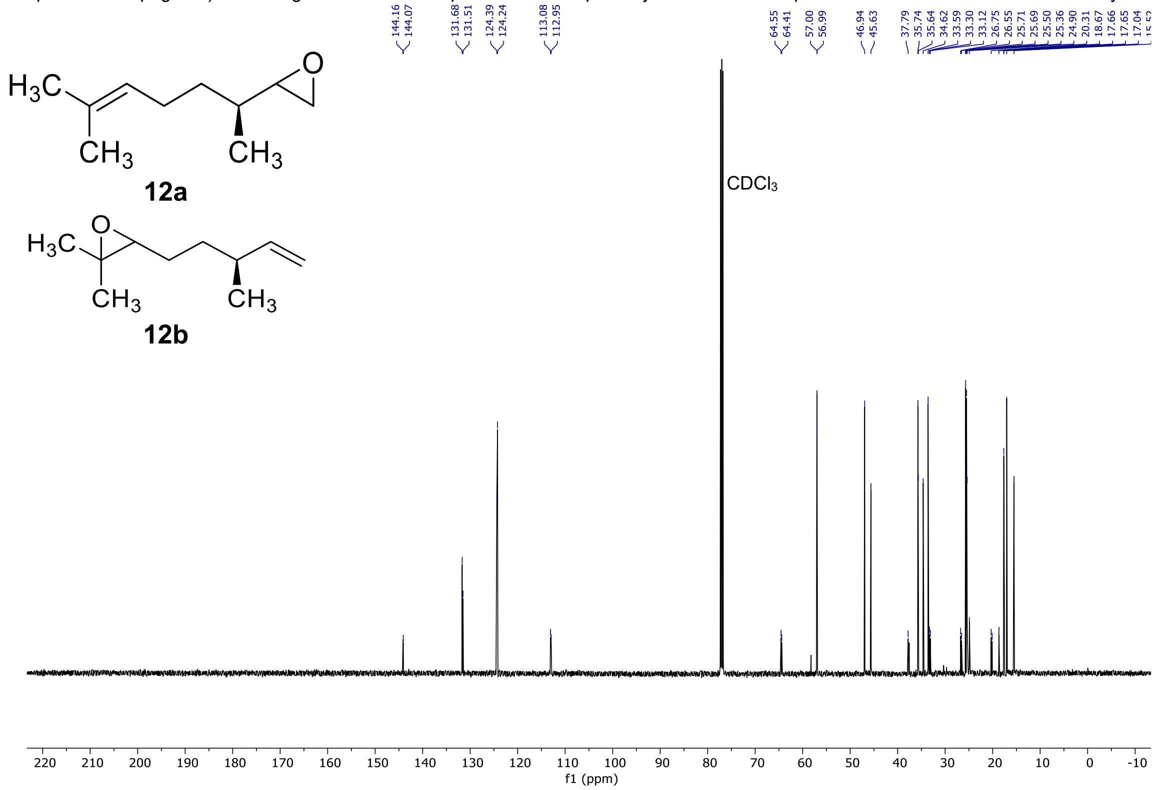

Figure S35: ^{13}C-NMR of (*S*)-citronellene oxides **12a** and **12b** in a 6:1 mixture (3:2 *syn:anti* for **12a**) isolated from the epoxidation reaction using salan **4c** (7125 MHz, $CDCl_3$, see experiment on page 29).

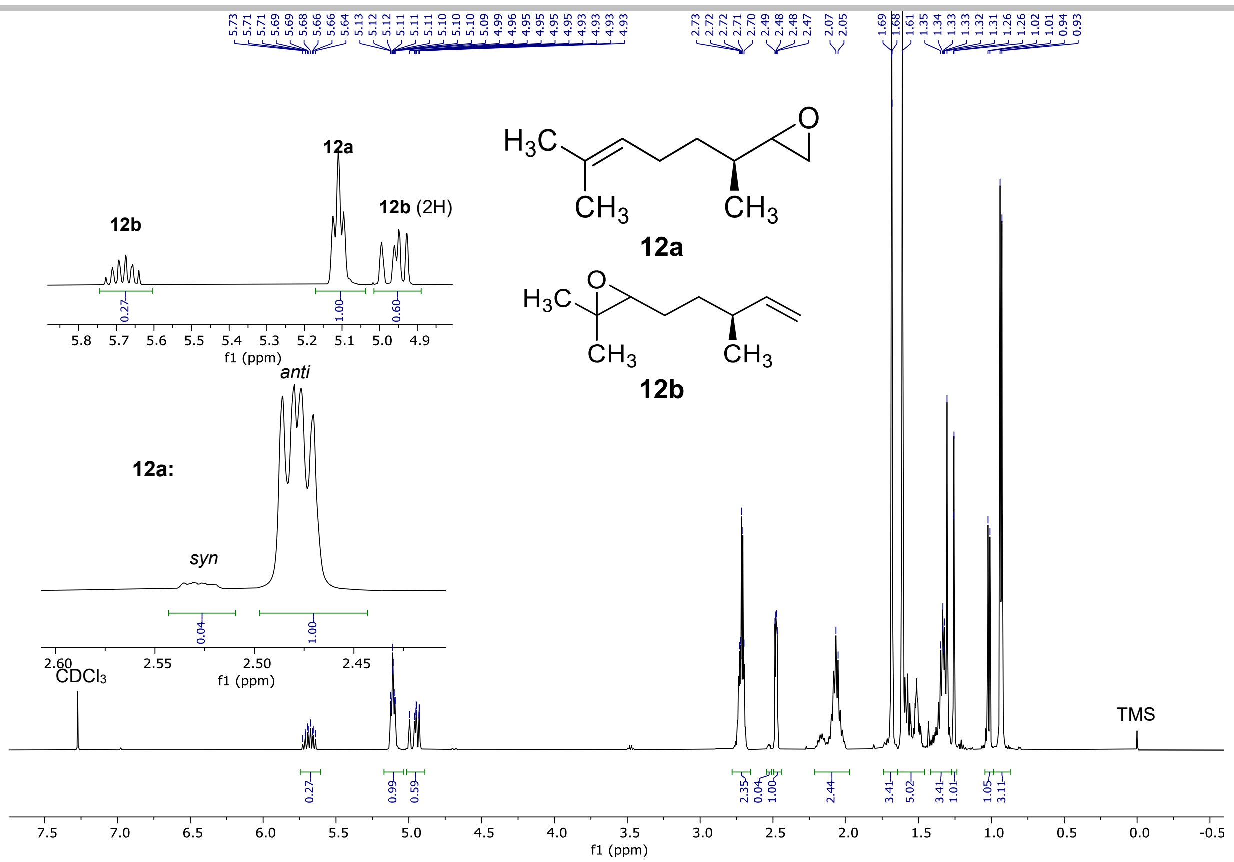

Figure S36: ^{1}H-NMR of (*S*)-citronellene oxides **12a** and **12b** in a 3:1 mixture (1:25 *syn:anti* for **12a**) isolated from the epoxidation reaction using salalen **2** (500 MHz, $CDCl_3$, see experiment on page 29). The integration in the excerpts was done separately from the whole spectrum to show the ratios more clearly.

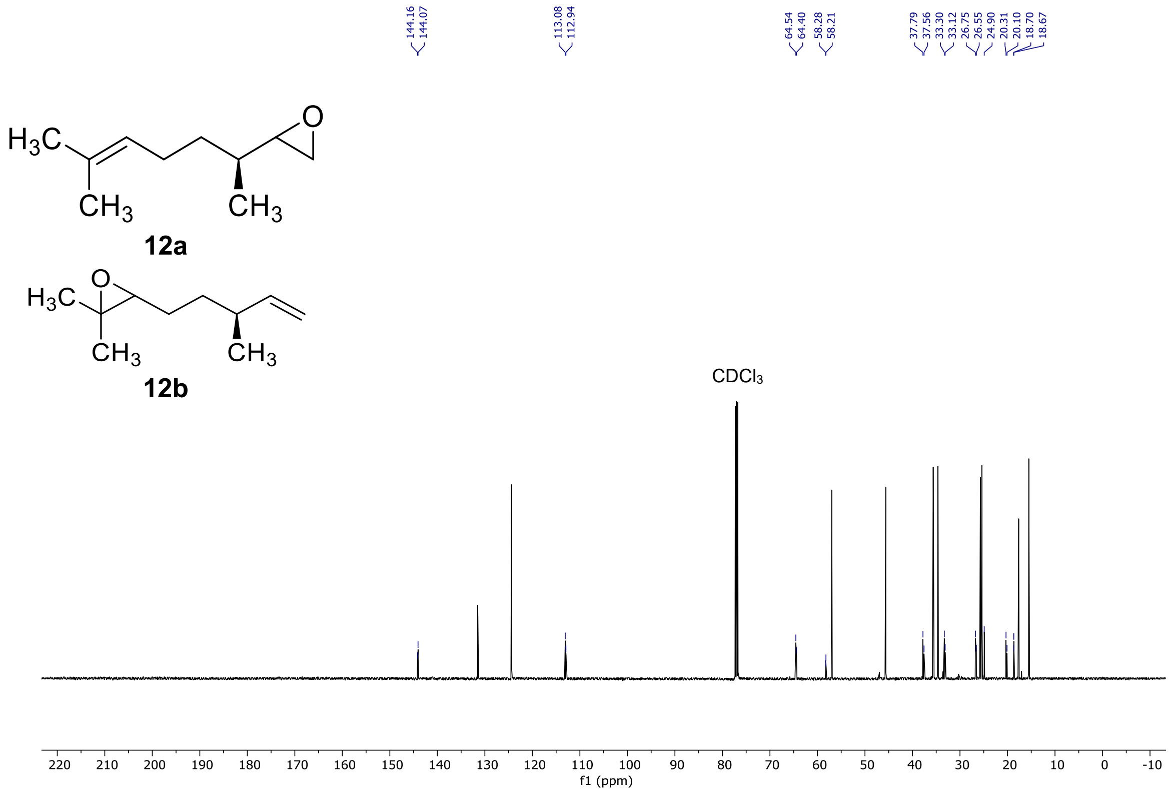

Figure S37: ^{13}C-NMR of (*S*)-citronellene oxides **12a** and **12b** in a 3:1 mixture (1:25 *syn:anti* for **12a**) isolated from the epoxidation reaction using salalen **2** (125 MHz, $CDCl_3$, see experiment on page 29).

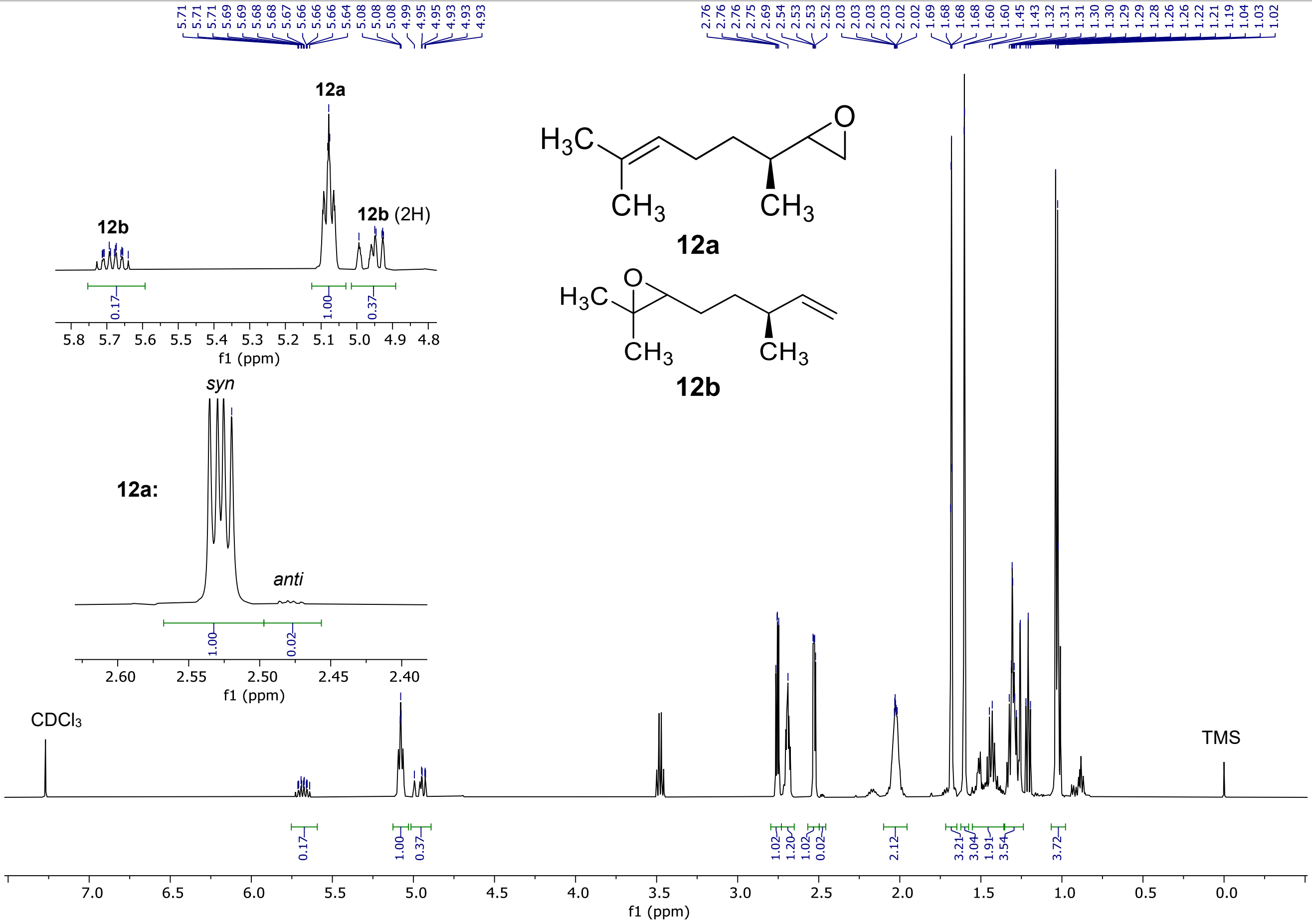

Figure S38: ^{1}H-NMR of (*S*)-citronellene oxides **12a** and **12b** in a 6:1 mixture (50:1 *syn:anti* for **12a**) isolated from the epoxidation reaction using salalen *ent*-**2** (500 MHz, $CDCl_3$, see experiment on page 29). The integration in the excerpts was done separately from the whole spectrum to show the ratios more clearly.

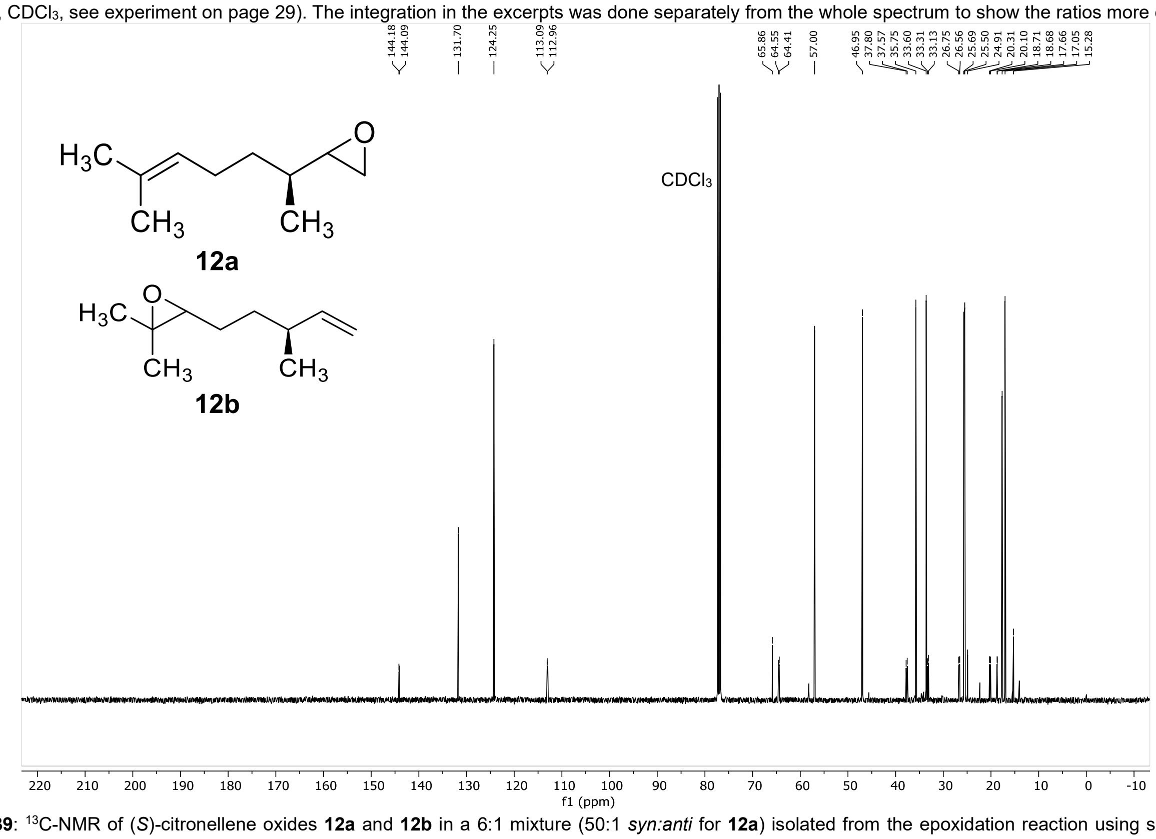

Figure S39: ^{13}C-NMR of (*S*)-citronellene oxides **12a** and **12b** in a 6:1 mixture (50:1 *syn:anti* for **12a**) isolated from the epoxidation reaction using salalen *ent*-**2** (500 MHz, $CDCl_3$, see experiment on page 29).

*(R)-Linalool Oxides **14a** and **14b***

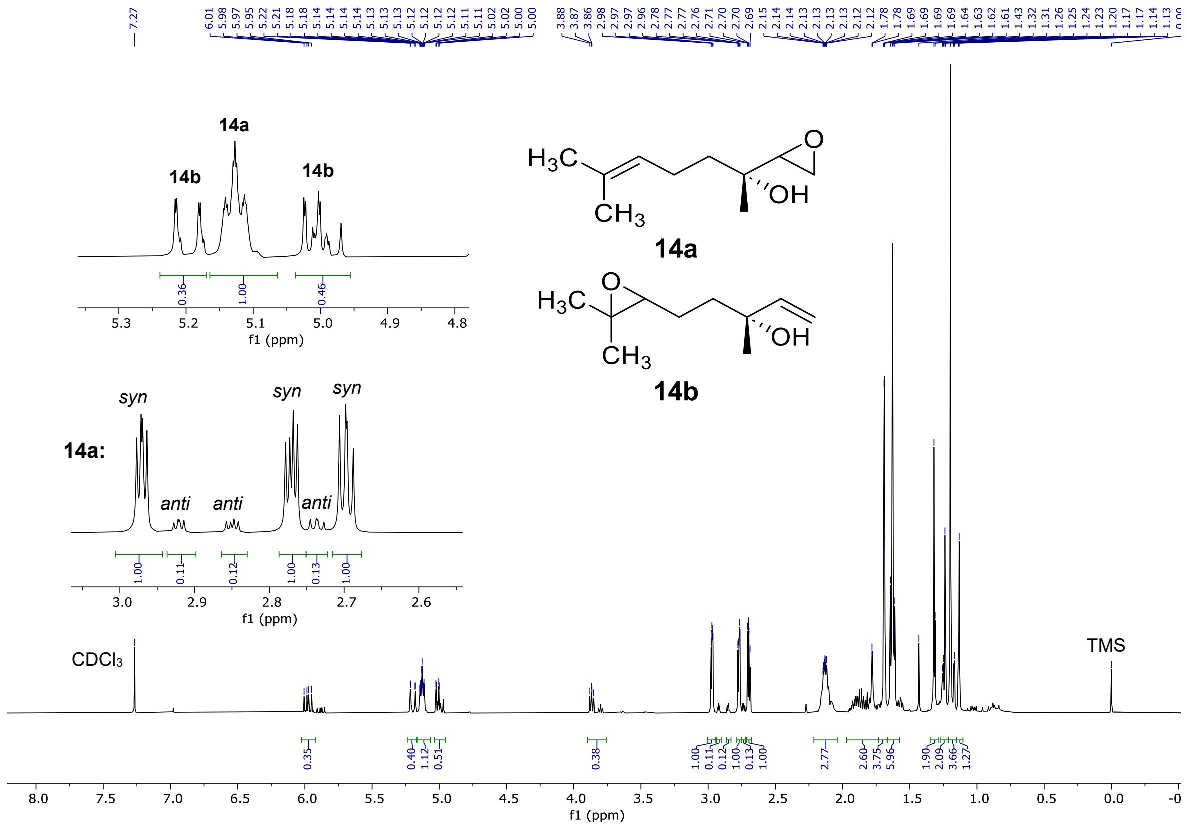

Figure S40: ^{1}H-NMR of (*R*)-linalool oxides **14a** and **14b** in a 3:1 mixture (9:1 *syn:anti* for **14a**) isolated from the epoxidation reaction using salan **4c** (500 MHz, $CDCl_3$, see experiment on page 30). The integration in the excerpts was done separately from the whole spectrum to show the ratios more clearly.

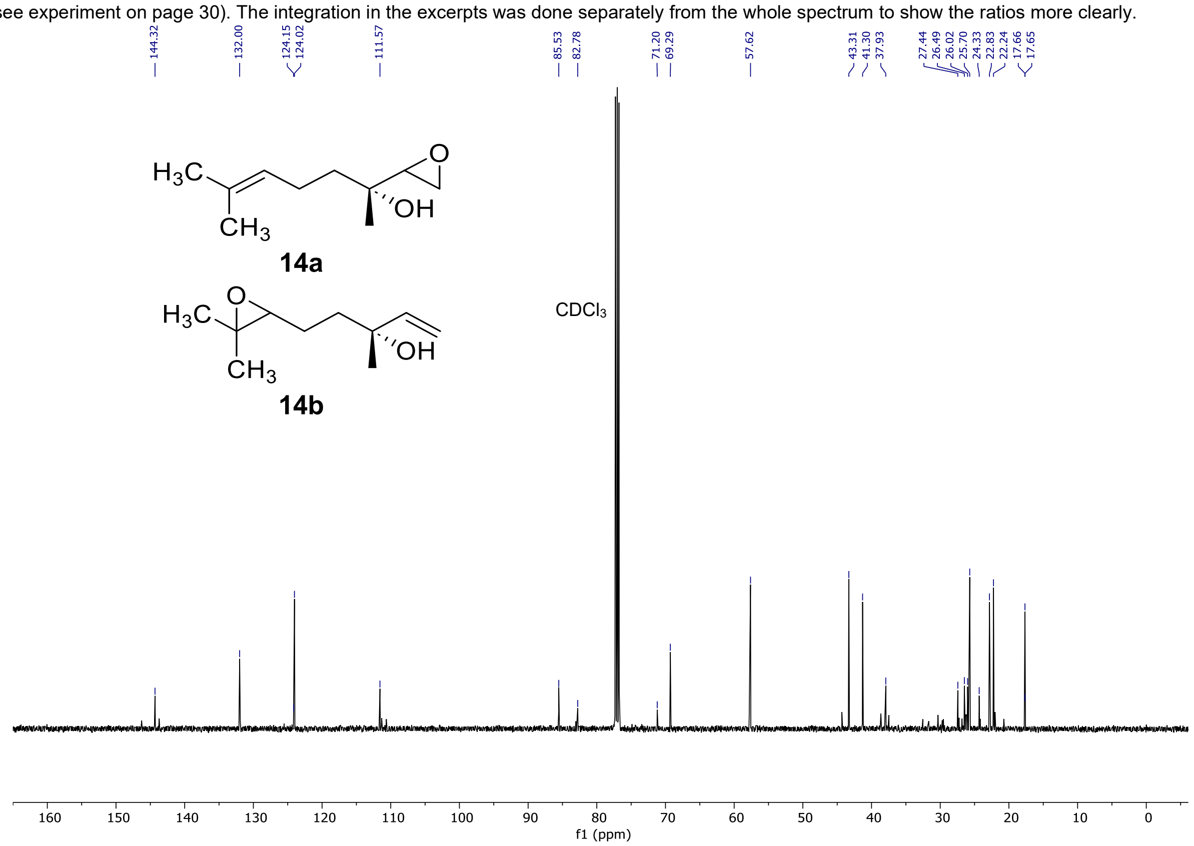

Figure S41: ^{13}C-NMR of (*R*)-linalool oxides **14a** and **14b** in a 3:1 mixture (9:1 *syn:anti* for **14a**) isolated from the epoxidation reaction using salan **4c** (125 MHz, $CDCl_3$, see experiment on page 30).

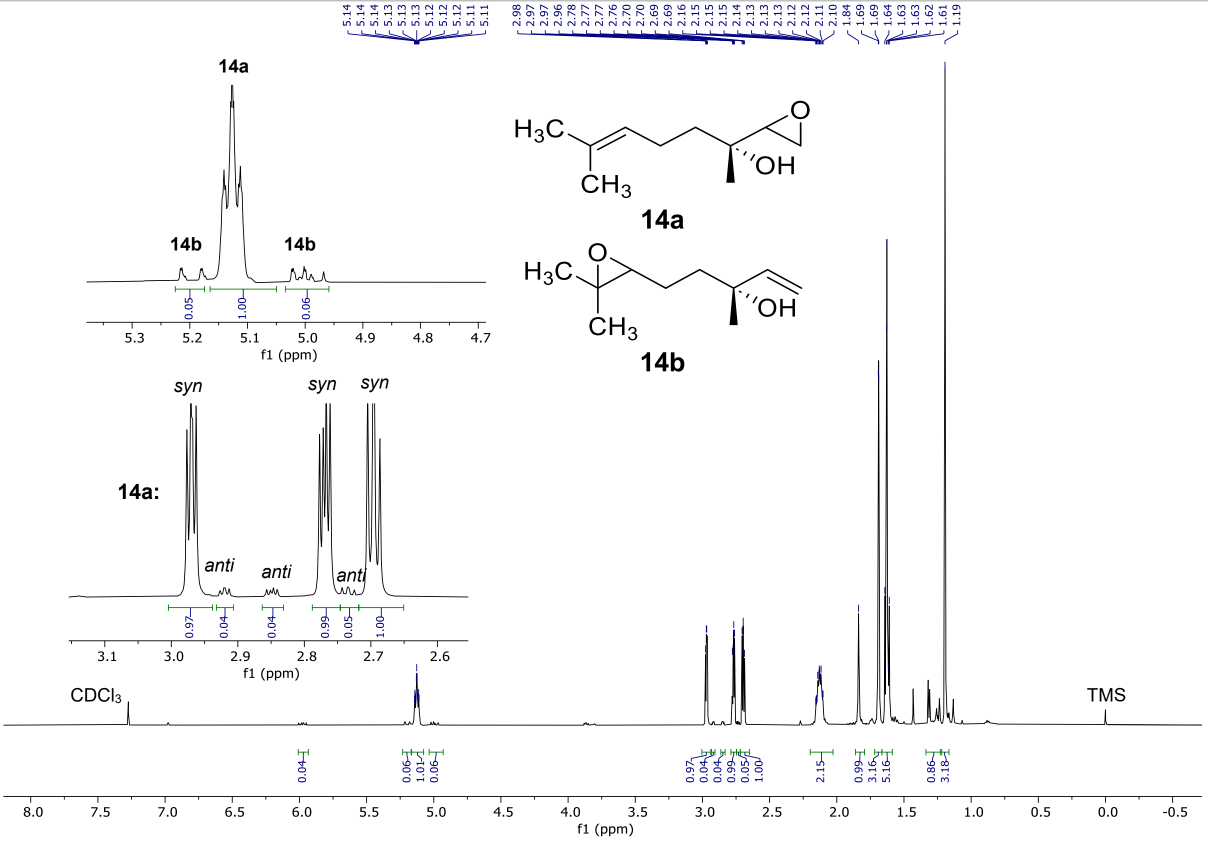

Figure S42: ^{1}H-NMR of (*R*)-linalool oxides **14a** and **14b** in a 19:1 mixture (24:1 *syn:anti* for **14a**) isolated from the epoxidation reaction using salalen **2** (500 MHz, $CDCl_3$, see experiment on page 30). The integration in the excerpts was done separately from the whole spectrum to show the ratios more clearly.

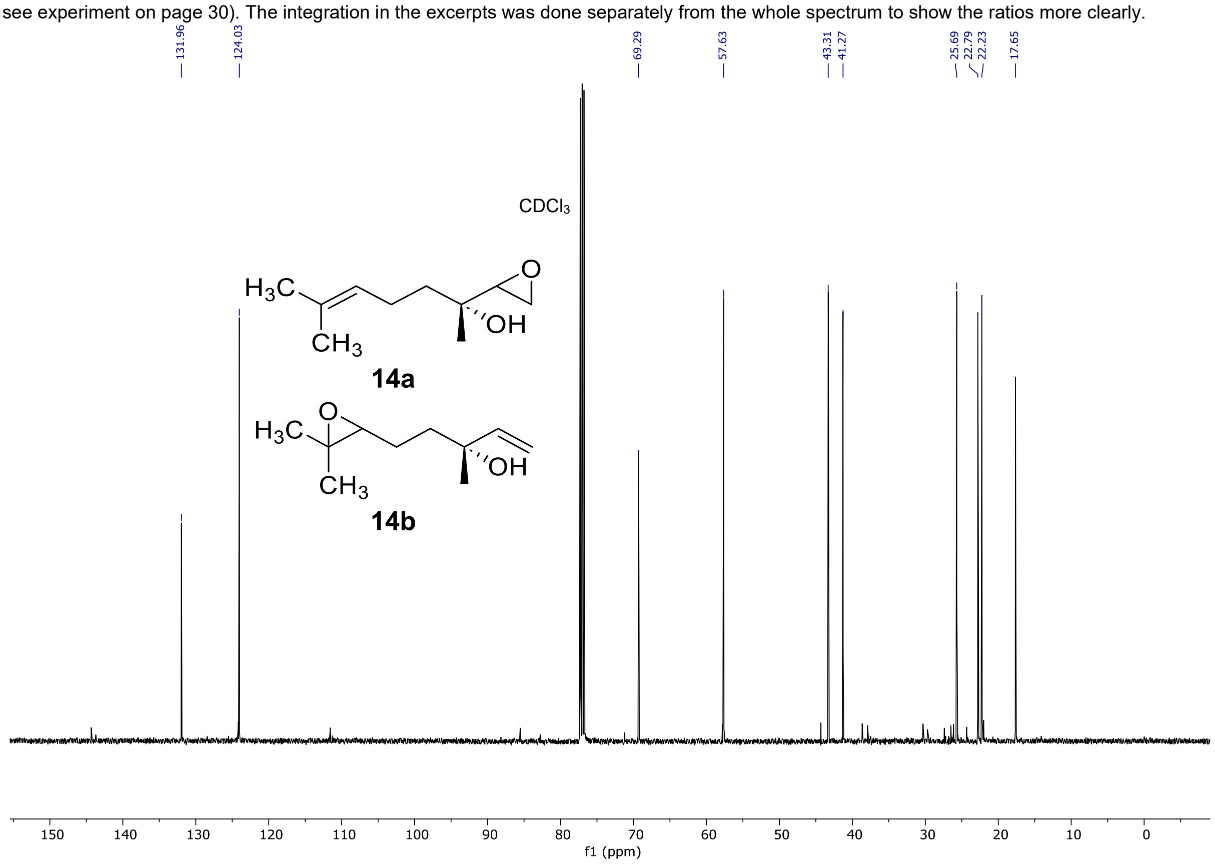

Figure S41: ^{13}C-NMR of (*R*)-linalool oxides **14a** and **14b** in a 19:1 mixture (24:1 *syn:anti* for **14a**) isolated from the epoxidation reaction using salalen **2** (125 MHz, $CDCl_3$, see experiment on page 30).

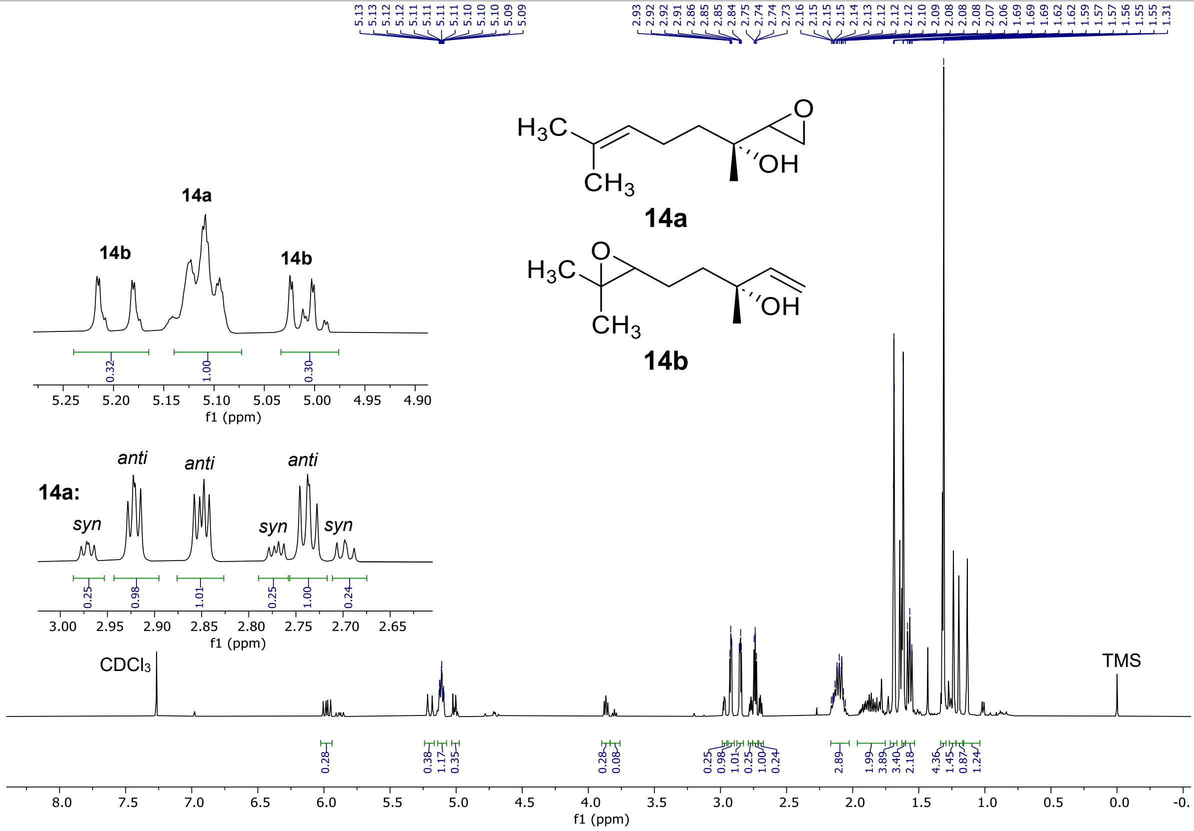

Figure S42: ^{1}H-NMR of (*R*)-linalool oxides **14a** and **14b** in a 3:1 mixture (1:4 *syn:anti* for **14a**) isolated from the epoxidation reaction using salalen *ent*-**2** (500 MHz, $CDCl_3$, see experiment on page 30). The integration in the excerpts was done separately from the whole spectrum to show the ratios more clearly.

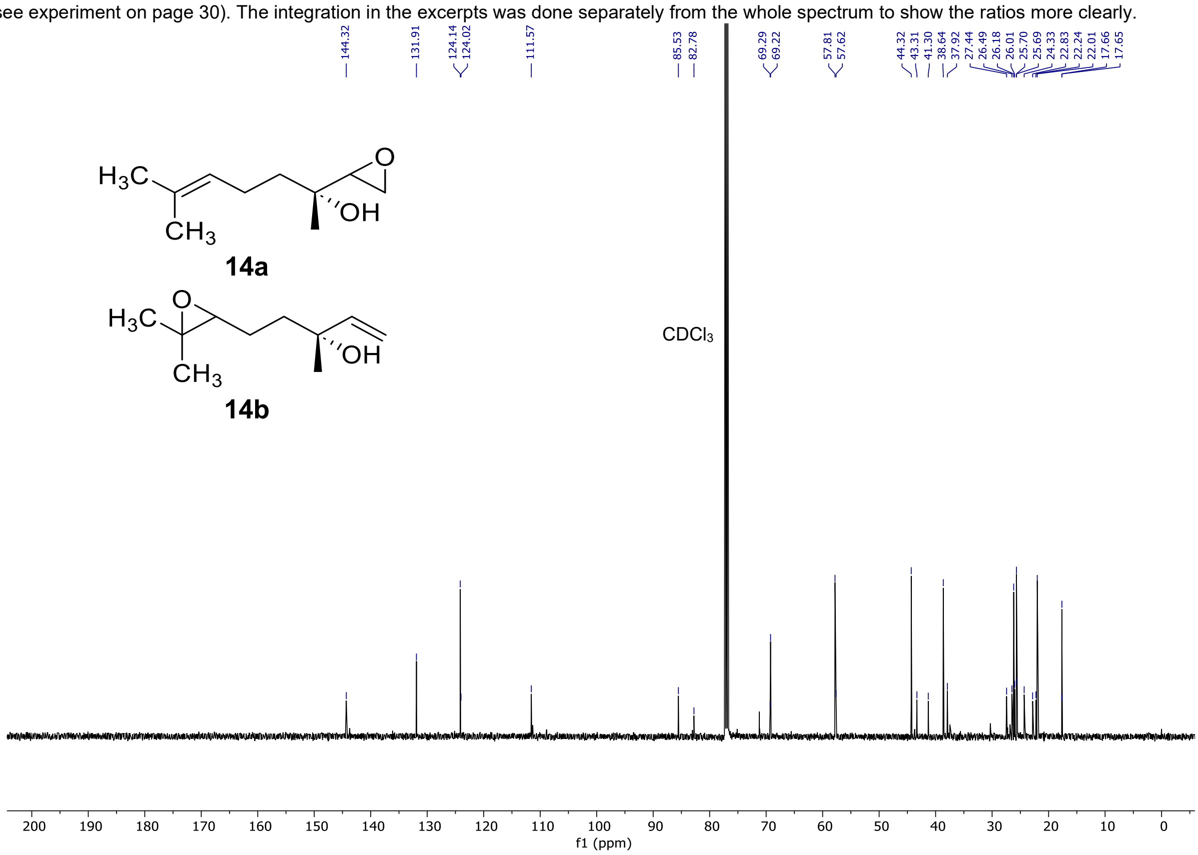

Figure S43: ^{13}C-NMR of (*R*)-linalool oxides **14a** and **14b** in a 3:1 mixture (1:4 *syn:anti* for **14a**) isolated from the epoxidation reaction using salalen *ent*-**2** (125 MHz, $CDCl_3$, see experiment on page 30).

3 GC

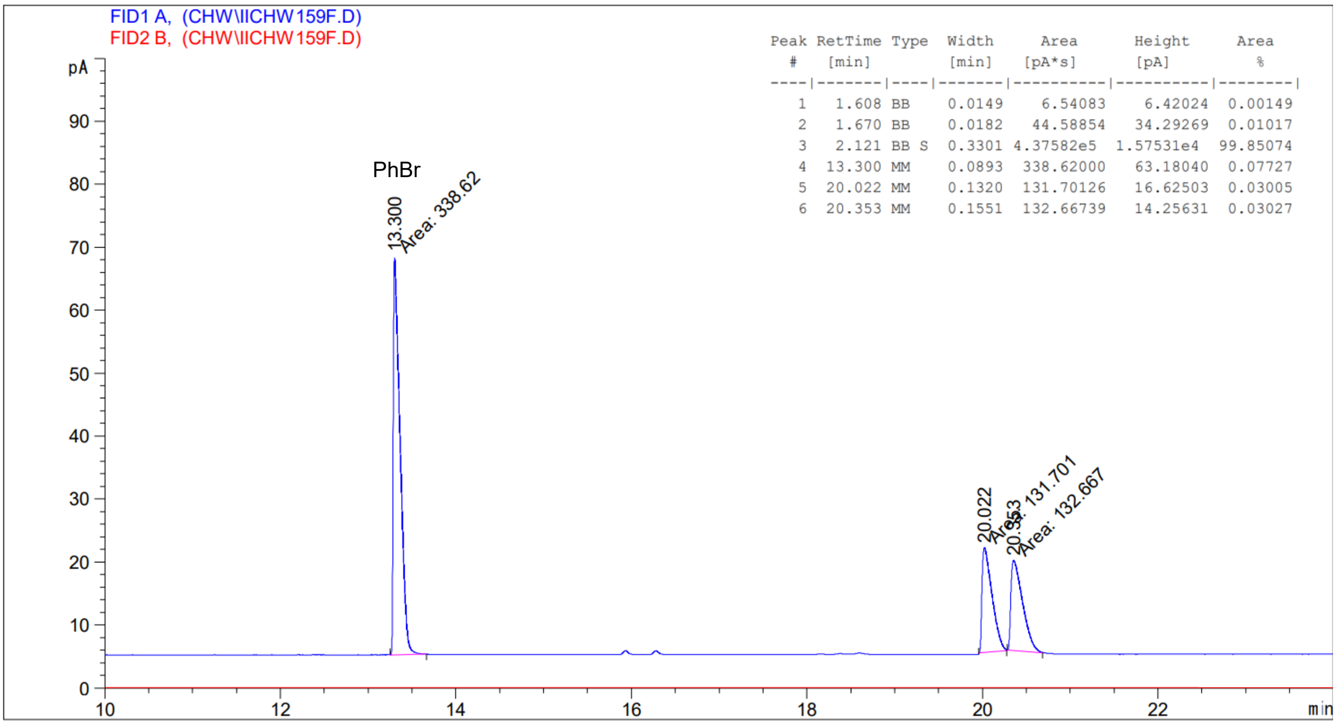

Figure S44: GC of racemic 5-bromo-1-pentene oxide *rac*-**8a** with internal standard PhBr obtained in the epoxidation using salan **4c** [see experiment page 11; GC-method: CP-Chirasil-DEX CB, 0.25 µm, 25 m x 0.25 mm, N_2; Method: 50 °C (5 min), 20 °C/min, 100 °C (10 min), 10 °C/min, 160 °C (5 min); split = 80:1; split flow = 48.0 mL/min, flow = 0.6 mL/min].

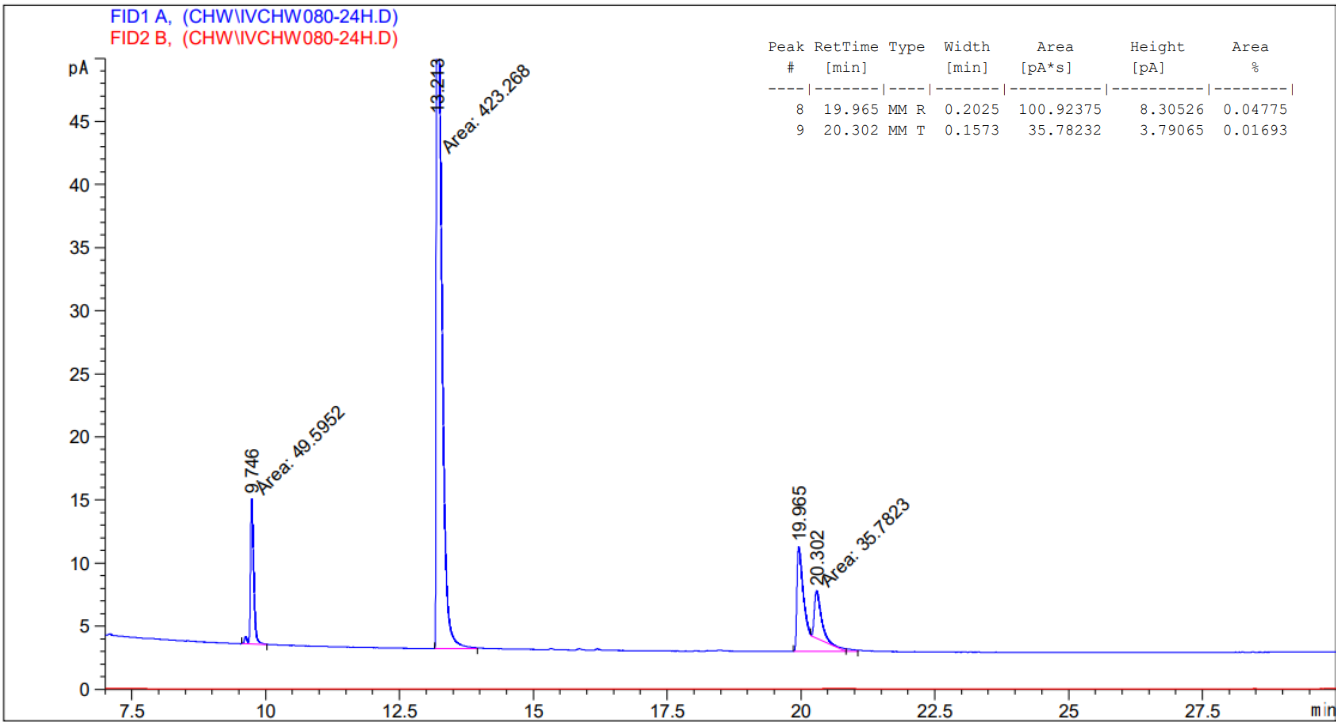

Figure S45: GC of 5-bromo-1-pentene oxide **8a** with internal standard PhBr obtained in the epoxidation using salan **4e** with a magnified excerpt of the chromatogram showing the integration of the product enantiomers [see experiment page 11, GC-method: CP-Chirasil-DEX CB, 0.25 µm, 25 m x 0.25 mm, N_2; Method: 50 °C (5 min), 20 °C/min, 100 °C (10 min), 10 °C/min, 160 °C (5 min); split = 80:1; split flow = 48.0 mL/min, flow = 0.6 mL/min)].

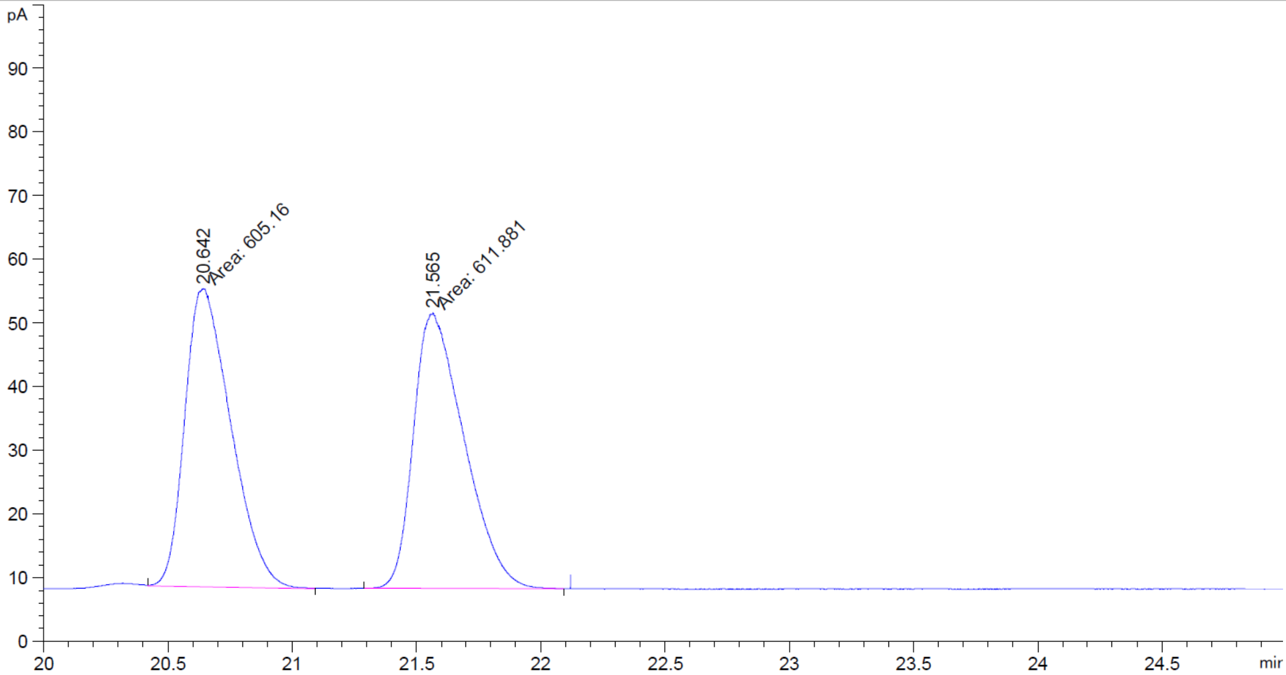

Figure S46: GC of the racemic myrcene oxide *rac*-**10a** obtained in the epoxidation using salan **4c** [see experiment on page 28, GC method: Hydrodex β-3P, 1 mL/min; 95 °C (24 min), 10 °C/min, 160 °C (5 min)].

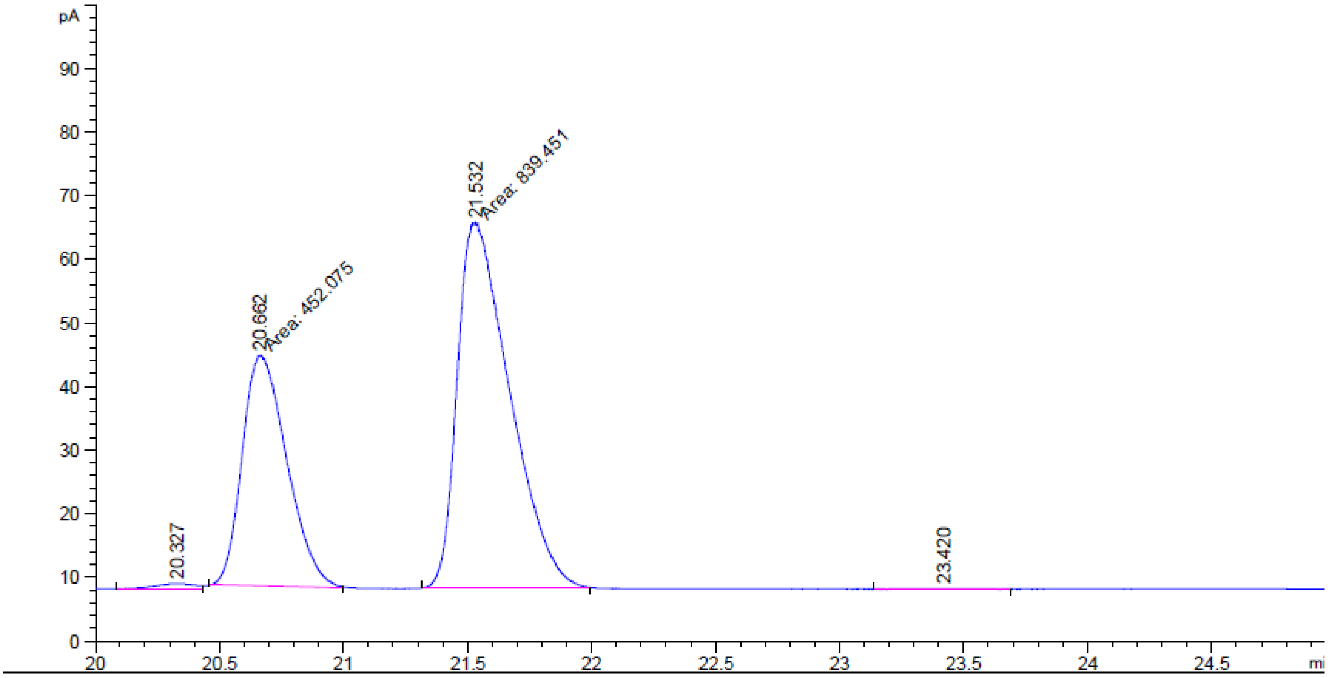

Figure S47: GC of myrcene oxide **10a** obtained in the epoxidation using salalen **2** [see experiment on page 28, GC method: Hydrodex β-3P, 1 mL/min; 95 °C (24 min), 10 °C/min, 160 °C (5 min)].

References

[1] a) H.-L. Mu, W.-P. Ye, D.-P. Song, Y.-S. Li, *Organometallics* **2010**, *29*, 6282-6290; b) A. Berkessel, T. Günther, Q. Wang, J.-M. Neudörfl, *Angew. Chem. Int. Ed.*, **2013**, *52*, *32*, 8467-8471.

[2] a) M. Lansing, H. Engler, T. M. Leuther, J.-M. Neudörfl, A. Berkessel, *ChemCatChem* **2016**, *8*, 3706-3709; b) C. Wartmann, S. Nandi, J.-M. Neudörfl, A. Berkessel, *Angew. Chem. Int. Ed*. **2023**, *62*, e202306584; *Angew. Chem.* **2023**, *135*, e202306584 .

[3] J. Chen, H. Gu, X. Zhu, W. Nam, B. Wang, *Adv. Synth. Catal.* **2020**, *362*, 2976-2983.

[4] A. K. Yudin, J. P. Chiang, H. Adolfsson, C. Copéret, *J. Org. Chem.* **2001**, *66*, 4713-4718.

[5] M. H. Shaw, R. A. Croft, W. G. Whittingham, J. F. Bower, *J. Am. Chem. Soc.* **2015**, *137*, 8054-8057.

[6] R. M. Roberts, L. W. Elrod, *J. Org. Chem.* **1981**, *46*, 3732-3735.

[7] G. Majetich, J. Shimkus, Y. Li, *Tetrahedron Lett.* **2010**, *51*, 6830-6834.

[8] W. Dai, S. Shang, B. Chen, G. Li, L. Wang, L. Ren, S. Gao, *J. Org. Chem.* **2014**, *79*, 6688-6694.

[9] B. Kang, M. Kim, J. Lee, Y. Do, S. Chang, *J. Org. Chem.* **2006**, *71*, 6721-6727.

[10] D. J. Vyas, E. Larionov, C. Besnard, L. Guénée, C. Mazet, *J. Am. Chem. Soc.* **2013**, *135*, 6177-6183.

[11] A. Steinreiber, S. F. Mayer, R. Saf, K. Faber, *Tetrahedron: Asymmetry* **2001**, *12*, 1519-1528.

[12] a) P. K. Mandal, G. Maiti, S. C. Roy, *J. Org. Chem*. **1998**, *63*, 2829-2834; b) T. Kawakami, I. Shibata, A. Baba, H. Matsuda, *J. Org. Chem.* **1993**, *58*, 7608- 7609.

[13] U. Krings, D. Hapetta, R. G. Berger, *Biocatal. Biotrans.* **2008**, *26*, 288-295.

[14] a) J. Swatschek, L. Grothues, J. O. Bauer, C. Strohmann, M. Christmann, *J. Org. Chem.* **2014**, *79*, 976-983; b) P. Winter, J. Swatschek, M. Willot, L. Radtke, T. Olbrisch, A. Schäfer, M. Christmann, *Chem. Commun.* **2011**, *47*, 12200-12202.

[15] Y. Q. Tu, A. Hübener, H. Zhang, C. J. Moore, M. T. Fletcher, P. Hayes, K. Dettner, W. Francke, C. S. P. McErlean, W. Kitching, *Synthesis* **2000**, *2000*, 1956-1978.

[16] S. Leisering, I. Riaño, C. Depken, L. J. Gross, M. Weber, D. Lentz, R. Zimmer, C. B. W. Stark, A. Breder, M. Christmann, *Org. Lett.* **2017**, *19*, 1478-1481.

[17] M. Hashimoto, H. Harigaya, M. Yanagiya, H. Shirahama, *J. Org. Chem.* **1991**, *56*, 2299-2311.
